# Microglia–neuron crosstalk through Hex–GM2–MGL2 maintains brain homeostasis

**DOI:** 10.1038/s41586-025-09477-y

**Published:** 2025-08-06

**Authors:** Maximilian Frosch, Takashi Shimizu, Emile Wogram, Lukas Amann, Lars Gruber, Ayelén I. Groisman, Maximilian Fliegauf, Marius Schwabenland, Chintan Chhatbar, Sabrina Zechel, Hendrik Rosewich, Jutta Gärtner, Francisco J. Quintana, Joerg M. Buescher, Thomas Blank, Harald Binder, Christine Stadelmann, Johannes J. Letzkus, Carsten Hopf, Takahiro Masuda, Klaus-Peter Knobeloch, Marco Prinz

**Affiliations:** 1 Institute of Neuropathology, Faculty of Medicine, University of Freiburg, Freiburg, Germany; 2 Center for Mass Spectrometry and Optical Spectroscopy (CeMOS), Mannheim University of Applied Sciences, Mannheim, Germany; 3 Medical Faculty, Heidelberg University, Heidelberg, Germany; 4 Institute for Physiology, Faculty of Medicine, University of Freiburg, Freiburg, Germany; 5 Department of Pharmaceutical Biology and Biotechnology, Institute of Pharmaceutical Sciences, University of Freiburg, Freiburg, Germany; 6 Department of Neuropathology, University Medical Center Göttingen, Göttingen, Germany; 7 Department of Child Neurology, Developmental Neurology, General Pediatrics, Diabetology, Endocrinology, Social Pediatrics, University Hospital Tübingen, Tübingen, Germany; 8 Division of Pediatric Neurology, Department of Pediatrics and Adolescent Medicine, University Medical Center Göttingen, Georg August University, Göttingen, Germany; 9 Ann Romney Center for Neurologic Diseases, Brigham and Women’s Hospital, Harvard Medical School, Boston, MA USA; 10 Broad Institute of MIT and Harvard, Cambridge, MA USA; 11 Max Planck Institute of Immunobiology and Epigenetics, Freiburg, Germany; 12 Institute of Medical Biometry and Statistics (IMBI), Faculty of Medicine and Medical Center, University of Freiburg, Freiburg, Germany; 13 Freiburg Center for Data Analysis, Modeling and AI, University of Freiburg, Freiburg, Germany; 14 Signalling Research Centres BIOSS and CIBSS, University of Freiburg, Freiburg, Germany; 15 BrainLinks-BrainTools, Intelligent Machine-Brain Interfacing Technology (IMBIT), University of Freiburg, Freiburg, Germany; 16 Mannheim Center for Translational Neuroscience (MCTN), Medical Faculty Mannheim, Heidelberg University, Mannheim, Germany; 17 Division of Molecular Neuroimmunology, Medical Institute of Bioregulation, Kyushu University, Fukuoka, Japan; 18 Center for Brain Research and Advancements In Neuroimmunology (BRAIN), Faculty of Medicine, University of Freiburg, Freiburg, Germany

**Keywords:** Diseases of the nervous system, Gliogenesis

## Abstract

As tissue-resident macrophages of the central nervous system parenchyma, microglia perform diverse essential functions during homeostasis and perturbations^1^. They primarily interact with neurons by means of synaptic engulfment and through the rapid elimination of apoptotic cells and non-functional synapses^2^. Here, by combining unbiased lipidomics and high-resolution spatial lipid imaging, deep single-cell transcriptome analysis and novel cell-type-specific mutants, we identified a previously unknown mode of microglial interaction with neurons. During homeostasis, microglia deliver the lysosomal enzyme β-hexosaminidase to neurons for the degradation of the ganglioside GM2 that is integral to maintaining cell membrane organization and function. Absence of *Hexb*, encoding the β subunit of β-hexosaminidase, in both mice and patients with neurodegenerative Sandhoff disease leads to a massive accumulation of GM2 derivatives in a characteristic spatiotemporal manner^3^. In mice, neuronal GM2 gangliosides subsequently engage the macrophage galactose-type lectin 2 receptor on microglia through *N*-acetylgalactosamine residues, leading to lethal neurodegeneration. Notably, replacement of microglia with peripherally derived microglia-like cells is able to break this degenerative cycle and fully restore central nervous system homeostasis. Our results reveal a mode of bidirectional microglia–neuron communication centred around GM2 ganglioside turnover, identify a microgliopathy and offer therapeutic avenues for these maladies.

## Abstract (web summary)

A mode of bidirectional microglia–neuron crosstalk through Hex–GM2–MGL2 and centred around GM2 ganglioside turnover is essential for homeostatic regulation of central nervous system membrane components.

## Main

Tissue-resident macrophages in the central nervous system (CNS) exist in different anatomical locations where they perform distinct context-dependent functions^1^. Outside the parenchyma, CNS-associated macrophages (CAMs) are found in the brain interfaces as leptomeningeal macrophages, dural macrophages or perivascular macrophages, or in the choroid plexus^4–6^. There, CAMs are thought to control border integrity, for example, during stroke or autoimmune inflammation^7–9^. By contrast, parenchymal microglia support the function of neighbouring cells such as oligodendrocytes^10^ or neurons by producing trophic factors such as brain-derived neurotrophic factor^11^ and insulin-like growth factor 1 (IGF-1)^12^, and through the rapid elimination of apoptotic cells and non-functional synapses^13^.

Neuronal lipids are an important class of biomolecules with a wide range of essential biological functions and high structural diversity that determines their cellular location. For instance, whereas fatty acids, triglycerides and sterol lipids are mainly localized in neuronal cell organelles, sphingolipids such as sphingomyelin and glycosphingolipids are largely found at the neuronal plasma membrane. There, gangliosides, as typical glycosphingolipids, account for 10–12% of the total lipid content and form membrane microdomains (‘lipid rafts’) with a variety of cellular functions such as signal transduction, endocytosis and membrane trafficking^14^. During development, the composition of brain gangliosides changes from predominantly simple (GM3) to complex (GM2, GM1) gangliosides, which suggests a potential role for gangliosides in brain development^15^. The involvement of microglia in the process of neuronal glycosphingolipid turnover is largely unknown.

Recent transcriptomic profiling has revealed a broad repertoire of microglia-associated genes^6,16,17^, including *Hexb*, which encodes the β subunit (HEXB) of the dimeric lysosomal enzyme β-hexosaminidase (Hex). This enzyme catalyses the hydrolysis of terminal *N*-acetyl-hexosamine residues from various glycoconjugates, including glycolipids. Two major Hex isoenzymes exist: heterodimeric Hex A, composed of one α and one β subunit, and homodimeric Hex B, composed of two β subunits^18^. Only Hex A degrades the key physiological substrate GM2 ganglioside^19^. In humans, inherited deficiency of *HEXB* causes Sandhoff disease^20^. Although the genetic cause and GM2 accumulation are well established, the specific cellular contributions to ganglioside clearance in the CNS remain unclear. We hypothesized that microglial Hex is essential for ganglioside homeostasis and its deficiency may cause CNS-wide impairment of GM2 degradation, contributing to Sandhoff disease. Accordingly, we integrated various high-dimensional transcriptomic and lipidomic techniques to study ganglioside turnover in the mouse and human CNSs. Additionally, we generated cell-type-specific mutants and, using different chimeric transfer systems, describe a critical microglia–neuronal Hex–GM2–macrophage galactose-type lectin 2 (MGL2) axis that is essential for maintaining CNS homeostasis.

## *Hexb* is a stable microglia gene

**Fig. 1:**
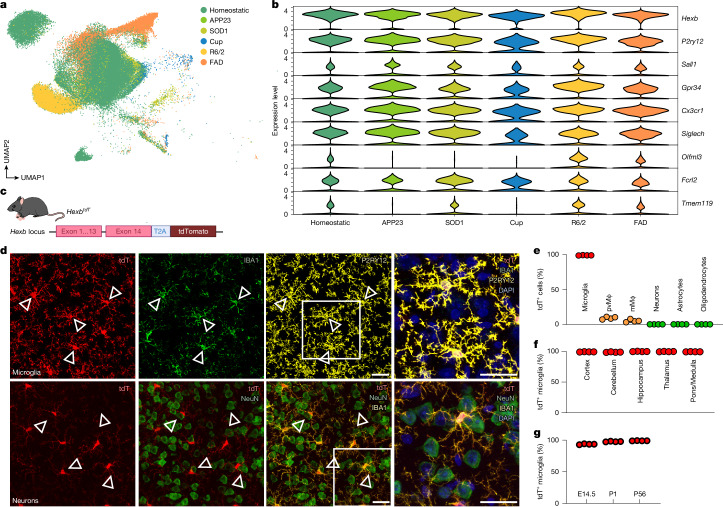
*Hexb* expression in the mouse brain is highly microglia-enriched throughout CNS conditions, brain regions and development. **a**, Uniform manifold approximation and projection (UMAP) of individual microglia from different conditions. 5xFAD and APP23 mice were used as models for Alzheimer’s disease, SOD1 mice for amyotrophic lateral sclerosis, R6/2 mice for Huntington’s disease and cuprizone-treated mice (Cup) to model demyelination. Each dot represents a single cell. Colours correspond to the condition investigated. Specific disease-associated microglia populations are detectable during demyelination and neurodegeneration. **b**, Violin plot depicting different microglial core genes and their expression during demyelination and neurodegeneration. **c**, Schematic overview of *Hexb*^*tdT*^ gene locus. A T2A–tdTomato cassette was inserted after exon 14 before the stop codon, allowing the expression of *tdT* and *Hexb* under the control of the endogenous *Hexb* gene locus. The self-cleaving peptide T2A ensures the separation of HEXB and tdT proteins. **d**, Representative immunofluorescence images of P56 *Hexb*^*tdT/tdT*^ mice showing high tdT positivity in P2RY12^+^ microglia (yellow) but not NeuN^+^ neurons (green) in the cortex. Triangles point to tdT^+^ microglia. **e**, Quantification of tdT^+^ CNS cells. Each symbol represents one individual mouse (*n* = 4), mean + s.e.m. is shown. At least 1,000 cells per individual were counted. mMϕ, leptomeningeal macrophage; pvMϕ, perivascular macrophage. **f**, Quantification of tdT^+^ microglia (IBA1^+^P2RY12^+^) in different brain regions at 56 days of age. Symbols represent individual mice (*n* = 4), mean + s.e.m. is shown. At least 1,000 cells per individual were counted. **g**, Quantification of tdT^+^ cortical microglia (IBA1^+^, green) at different ages (embryonic day 14.5 (E14.5), P1 and P56). Symbols represent individual mice (*n* = 4), mean + s.e.m. is shown. At least 1,000 cells per individual were counted. Scale bars, 25 μm. Illustration in **c** was created using BioRender. Frosch, M. (2025) https://BioRender.com/v74v524. Source data

Recent studies have sought to identify microglial genes, which robustly separates them from CAMs or other brain-resident cells, aiming to target these cells specifically^21,22^. Key genes include *P2ry12*, *Tmem119*, *Sall1* and *Hexb*. To identify microglial genes that are robustly expressed during pathology, we performed single-nucleus 3′ mRNA sequencing (snRNA-seq) of microglia in five models of neurodegeneration or demyelination. As expected, several context-dependent microglial clusters emerged (Fig. 1a). Among core microglial genes, *Hexb*, *P2ry12* and *Cx3cr1* showed consistently high expression, whereas *Tmem119*, *Sall1*, *Gpr34*, *Siglech*, *Olfml3* and *Fcrl2* were either low or variable (Fig. 1b).

**Extended Data Fig. 1:**
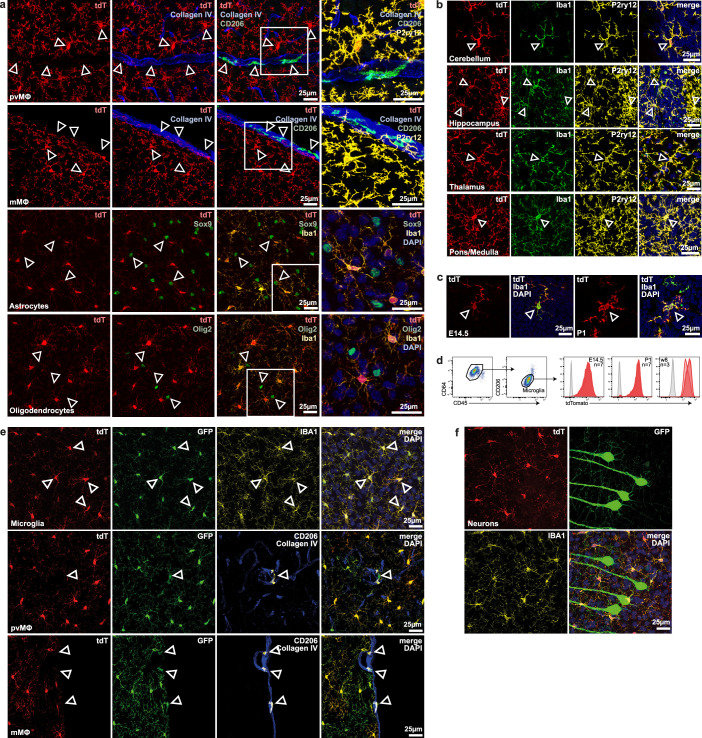
*Hexb* expression in the mouse brain is highly microglia-restricted throughout brain regions and development. A) Representative immunofluorescence images of P56 *Hexb*^*tdT/tdT*^ mice showing no tdT positivity in CD206^+^ perivascular and leptomeningeal macrophages (green), SOX9^+^ astrocytes (green) or OLIG2^+^ oligodendrocytes (green) in the cortex. Triangles point to tdT^+^ microglia. pvMΦ: perivascular macrophage, mMΦ: leptomeningeal macrophage. B) Typical immunofluorescence images of tdT^+^ microglia (IBA1^+^P2RY12^+^) in different brain regions at 56 days of age. C) Immunofluorescence images of tdT^+^ cortical microglia (IBA1^+^, green) at different ages (embyronic (E) day 14.5, postnatal (P) days 1 and 56. D) Expression of tdT by microglia in *Hexb*^*tdT/tdT*^ mice measured by flow cytometry. Histograms show the expression levels of tdT at different ages. *Hexb*^*+/+*^ mice are used as controls. Each histogram displays all individual data derived from the indicated number of mice. E) Immunofluorescence images of P56 *Hexb*^*tdT/tdT*^:*Cx3cr1*^*GFP/+*^ mice showing tdT and GFP overlap in IBA1^+^ microglia but not CD206^+^ perivascular or meningeal macrophages. Triangles point to tdT^+^ microglia or tdT^*−*^ CAMs. F) Immunofluorescence images of P56 *Hexb*^*tdT/tdT*^:*Thy1*^*GFP/+*^ mice showing tdT expression only in IBA1^+^ microglia but not GFP^+^ neurons.

To examine *Hexb* regulation in health, we took advantage of our *Hexb*^*tdT*^ reporter line^23^ (Fig. 1c). Of note, virtually all cortical microglia expressed tdTomato (99.06 ± 0.10%), unlike perivascular macrophages (9.10 ± 0.89%), leptomeningeal macrophages (5.04 ± 0.99%), neurons (0.01 ± 0.01%), astrocytes (0.03 ± 0.03%) and oligodendrocytes (0%) (Fig. 1d,e and Extended Data Fig. 1a). Moreover, microglia were tdTomato^+^ across brain regions and kept *Hexb* expression over development (Fig. 1f,g and Extended Data Fig. 1b–d). Crossbreeding of *Hexb*^*tdT*^ mice with *Cx3cr1*^*GFP*^ or *Thy1*^*GFP*^ confirmed no overlap of *Hexb*-expressing microglia with GFP^+^ CAMs or neurons, respectively (Extended Data Fig. 1e,f). Previous reports of low *Hexb* mRNA in mouse neurons^24^ were confirmed in adult wild-type mice (data not shown). In sum, *Hexb* is a highly stable microglial gene throughout the mouse CNS during development, homeostasis and disease.

## Early and robust microglial activation

**Fig. 2:**
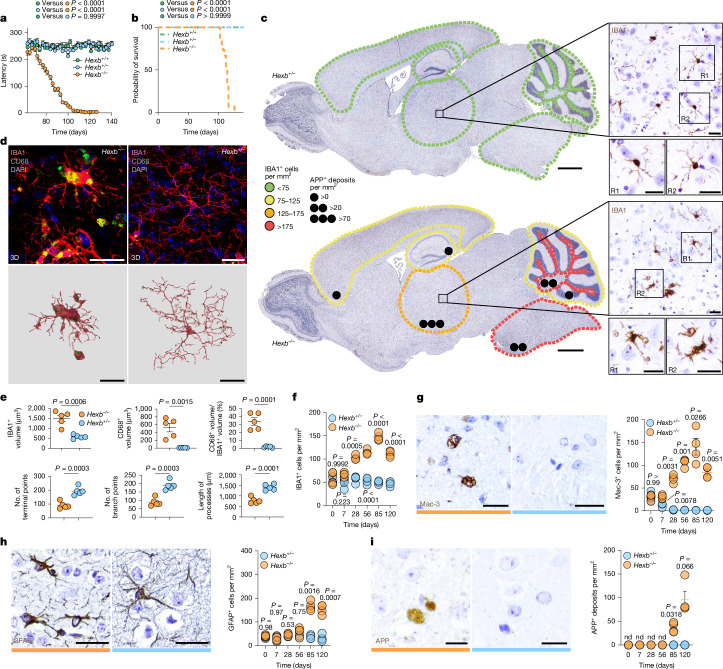
Widespread and pronounced early-onset lysosomal activation of microglia is a key feature of *Hexb-*mediated pathology. **a**, Latency to fall in the rotarod assay for *Hexb*^*−/−*^ (*n* = 15), *Hexb*^*+/−*^ (*n* = 15) and *Hexb*^*+/+*^ (*n* = 15) mice. **b**, Kaplan–Meier survival curve of *Hexb*^*−/−*^ (*n* = 15), *Hexb*^*+/−*^ (*n* = 15) and *Hexb*^*+/+*^ (*n* = 15) animals. **c**, Immunohistochemical pictures of sagittal brain sections from P120 *Hexb*^*−/−*^ and *Hexb*^*+/−*^ mice showing IBA1^+^ microglia (brown). Microglial density (colour-based) and APP^+^ deposits (black dots) are indicated (*n* = 4 per group). **d**, Top, immunofluorescence images of IBA1^+^ microglia (red), highlighting CD68^+^ lysosomes (green) from the thalamus at P120. Bottom, 3D reconstruction of IBA1^+^ microglia (red) and CD68^+^ lysosomes (green). **e**, Quantitative analysis of microglial morphologies. At least three cells per mouse were measured. **f**, Quantification of IBA1^+^ microglia in the thalamus over disease course. **g**, Representative immunohistochemical pictures of Mac-3^+^ microglia from the thalamus at P120 (left) and quantification thereof (right). At least 500 cells per mouse were measured. **h**, Immunohistochemical images from the thalamus at P120 (left) and quantification (right) of GFAP^+^ astrocytes. At least 300 cells per mouse were measured. **i**, Typical immunohistochemical pictures from the thalamus at P120 (left) and quantification (right) of APP^+^ deposits in the thalamus. At least 300 deposits per mouse were measured. nd, not detected. Data are shown as mean ± s.e.m. Statistical analyses: one-way analysis of variance (ANOVA) with Tukey’s post hoc test (**a**); log-rank test (**b**); two-tailed Student’s *t*-test (**e**); two-way ANOVA with Sidak’s test (**f**–**i**); each symbol in **e**–**i** represents an individual mouse. The colour code represents the genotype (orange, *Hexb*^*−/−*^; blue, *Hexb*^*+/−*^). Scale bars, 1 mm (**c** (main images)), 25 μm (**c** (magnified images),**d**,**g**,**h**,**i**). Source data

**Extended Data Fig. 2:**
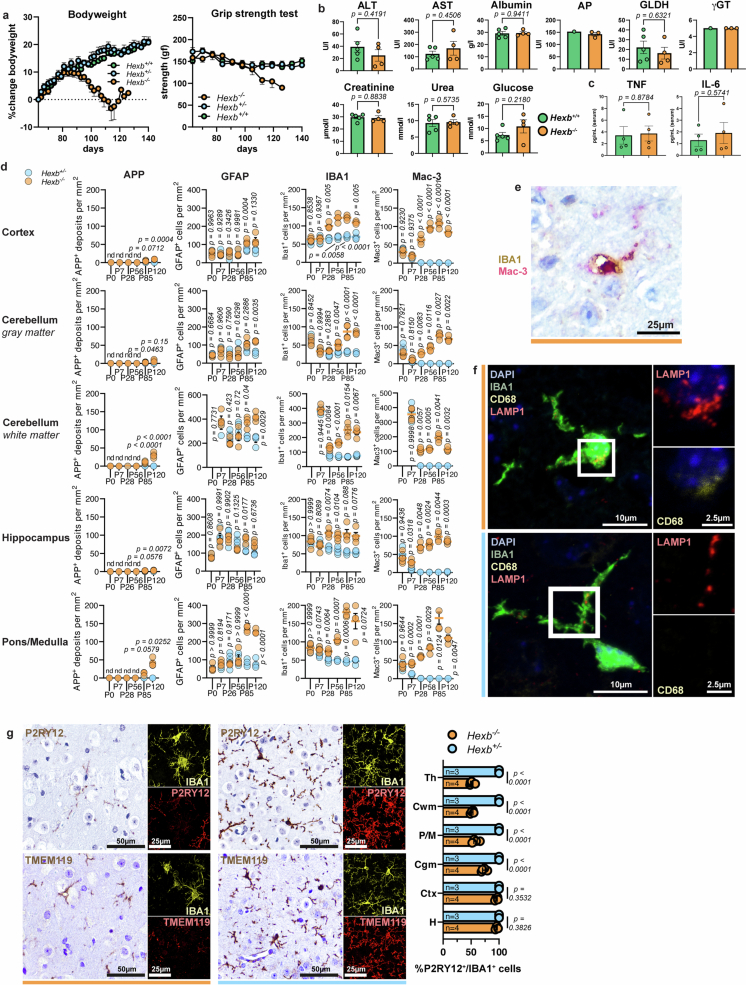
*Hexb* deficiency leads to microglial activation, astrogliosis, and axonal damage throughout the mouse brain with regional differences. A) Development of body weight over disease course (left) and strength in the Grip Strength Test (right) for *Hexb*^*−/−*^ (n = 15), *Hexb*^*+/−*^ (n = 15), and *Hexb*^*+/+*^ (n = 15) controls. B) Measurement of blood marker for liver and kidney damage in *Hexb*^*+/−*^ and *Hexb*^*−/−*^ mice. One symbol indicates one biological replicate. C) TNF and IL-6 levels in the blood of *Hexb*^*+/−*^ and *Hexb*^*−/−*^ mice measured by ELISA. Paired t-test comparison was used for statistical testing. D) Quantification of APP (axonal damage), GFAP (astrocytosis), IBA1 (microgliosis), and Mac-3 (lysosomal microglia activation) in different brain regions (cortex, cerebellum grey and white matter, hippocampus, thalamus, and pons/medulla) at different time points (P0, P7, P28, P56, P85, P120) for *Hexb*^*−/−*^ (n = 4) and *Hexb*^*+/−*^ (n = 4) controls. E) Representative immunohistochemical image of IBA1/Mac-3 double-positive cells in the thalamus of P120 *Hexb*^*−/−*^ mice. F) Representative immunofluorescence images indicating lysosomal activation in P7 *Hexb*^*−/−*^ (orange) and *Hexb*^*+/−*^ (blue) microglia. G) Left: Representative immunohistochemical images of P2RY12^+^ and TMEM119^+^ cells in the thalamus at P120Right: Quantification Each dot represents one individual mouse. Th: thalamus, Cwm: Cerebellum – white matter, P/M: pons/medulla, Cgm: Cerebellum – grey matter, Ctx: cortex, H: hippocampus. Data shown as mean ± s.e.m. Statistical analyses: Two-tailed Student’s t-test was used for statistical testing (B-C); Two-way ANOVA followed by Sidak’s multiple comparison test was used for statistical testing (D,G). Source data

Having proven that microglia expressed *Hexb* at high levels in the mouse brain, we next examined its functional role. *Hexb* knockout (*Hexb*^*−/−*^) mice—previously generated as a Sandhoff disease model^25^—developed rapidly progressing ataxia and weight loss, with preserved grip strength, and died at postnatal day (P) 114 ± 12 (Fig. 2a,b and Extended Data Fig. 2a). Notably, no peripheral inflammatory causes of an encephalopathy causing motor symptoms were found in the blood (Extended Data Fig. 2b,c), and brains lacked lymphocytic infiltrates (data not shown). By contrast, microglia showed marked changes in number and morphology (Fig. 2c–f and Extended Data Fig. 2d). Microglial cell numbers increased by P28 and peaked at P85, indicating early involvement before clinical onset. Densities varied regionally, with the highest counts in thalamus, pons/medulla and cerebellar white matter (Fig. 2c). Three-dimensional (3D) reconstruction of IBA1^+^ microglia and CD68^+^ lysosomes showed enlarged lysosomal volumes, fewer terminal/branch points and shortened processes (Fig. 2d,e). Moreover, microglia upregulated the lysosomal activation marker Mac-3 as early as P7 (Fig. 2g and Extended Data Fig. 2d–f) and downregulated TMEM119 and P2RY12, highlighting their activated phenotype. P2RY12 loss was most evident in the thalamus (51.29 ± 4.23% of IBA1^+^ microglia) (Extended Data Fig. 2g). Astrogliosis appeared only at P85, when mice were already affected clinically (Fig. 2h and Extended Data Fig. 2d), whereas APP^+^ extracellular deposits, a sign of axonal damage, emerged late, especially in the thalamus (Fig. 2c,i and Extended Data Fig. 2d). In sum, lack of *Hexb* drives early and excessive microglial activation reflected by pronounced numeric, morphological and lysosomal changes, whereas astrogliosis and axonal injury are late events.

## Molecular census of *Hexb*^*−/−*^ CNS cells

**Fig. 3:**
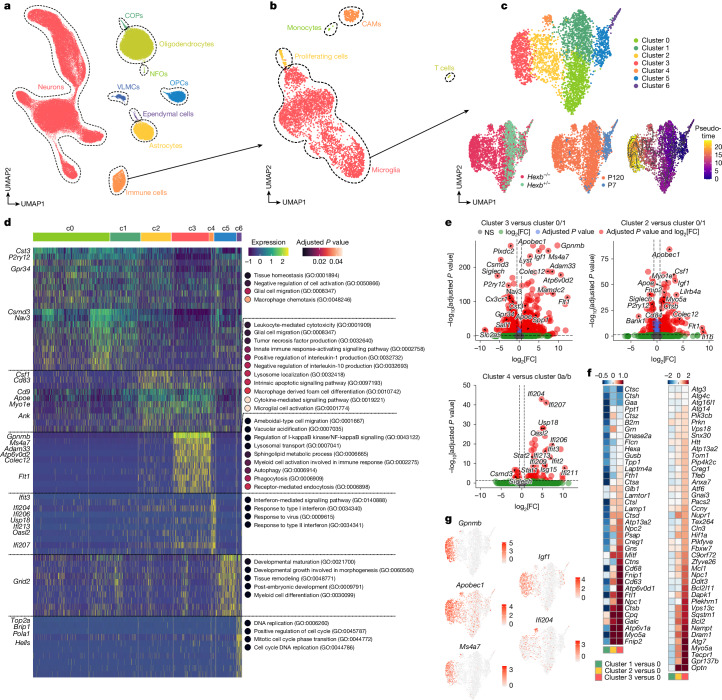
Molecular census of *Hexb-*deficient mouse brains. **a**, UMAP visualization of 103,201 individual nuclei from the thalamus of P7 and P120 *Hexb*^*−/−*^ and *Hexb*^*+/−*^ mice captured by snRNA-seq. COP, committed oligodendrocyte precursor cell; NFO, newly formed oligodendrocyte; OPC, oligodendrocyte precursor cell; VLMC, vascular leptomeningeal cell. **b**, UMAP visualization of the immune cell subset. **c**, UMAP visualization of the microglia subset, coloured by cluster identity (top), genotype (bottom left), age (bottom middle), and pseudotime (bottom right). **d**, Heat map of genes (rows) and dot plots for GO terms associated with each cluster of microglia shown in **c**. Key genes are highlighted. Dot plots show selected and enriched GO terms of the respective cluster. Colours in the heat map correspond to normalized scaled expression. Dot colour reflects the adjusted *P* value from a hypergeometric over-representation test with Benjamini–Hochberg correction applied for multiple comparisons. **e**, Volcano plots show the differentially expressed genes (DEGs) between the indicated microglial clusters shown in **c**. MAST test was used for statistical testing. FC, fold change; NS, not significant. **f**, Heat maps showing the levels (log_2_(FC)) of lysosome (left) and autophagy (right) pathway-related genes, comparing the clusters shown in **c**. **g**, Feature plots depicting most significant DEGs in disease clusters.

**Extended Data Fig. 3:**
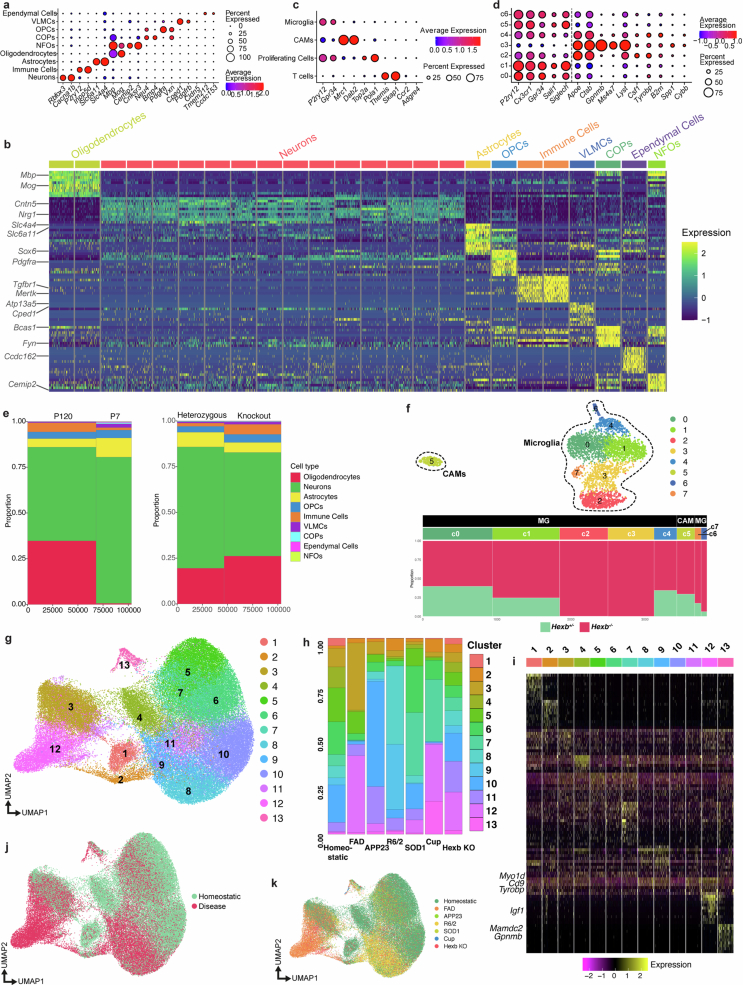
snRNA-seq reveals CNS cell-type composition and microglial heterogeneity across disease states. A) Selected marker genes associated with each cell type highlighted in Fig. 3a. B) Typical marker genes associated with each immune cell type highlighted in Fig. 3b. C) Dotplot depicting common microglial homeostatic and disease-associated genes among clusters shown in Fig. 3c. D) Heat map featuring the top cell-type-specific marker genes across the major cell types. The color bar indicates gene expression. E) Marimekko plot depicting the different cell type compositions separated by age or genotype. F) UMAP and Marimekko chart of microglia and CAMs depicting the proportions of *Hexb*^*−/−*^ and *Hexb*^*+/−*^ microglia for each cluster. G) UMAP visualization of microglia cluster from different conditions shown in Fig. 1a. Here, microglia from P120 *Hexb*^*−/−*^ microglia are integrated. H) Marimekko plot depicting the cluster proportions for the indicated conditions. I) Heat map featuring the top cluster marker genes. Shared *Hexb*^*−/−*^ disease genes are highlighted. J) UMAP visualization of microglia shown in **g**. Color code indicates their belonging to the homeostatic or disease condition. K) UMAP visualization of microglia shown in **g**. Color code indicates the respective condition.

To explore the molecular basis of microglial activation in *Hexb*^*−/−*^ mice, we performed snRNA-seq on thalamic nuclei at P7 and P120. Heterozygous mice (*Hexb*^*+/−*^) served as controls, as they showed no expression differences compared with *Hexb*^+/+^ microglia (Supplementary Fig. 1). The thalamus was chosen for transcriptomic profiling because of the pronounced microgliosis, severe axonal damage and its known involvement in Sandhoff disease in humans^26^. After quality control, we analysed 103,201 nuclei, with cell types assigned using both the Azimuth tool and reference gene sets^9^ (Fig. 3a and Extended Data Fig. 3a,b). Immune cell proportions increased notably in aged *Hexb*^*−/−*^ mice (Fig. 3b and Extended Data Fig. 3c,e). Within the immune population, we identified seven distinct microglia clusters. Clusters c0 and c1 comprised virtually all adult control microglia, thus representing ‘homeostatic’ clusters. Clusters c2–4 were overrepresented in diseased knockouts, whereas neonatal microglia (c5) or CAMs lacked disease-associated clusters, suggesting a pure microglial involvement in disease (Fig. 3c and Extended Data Fig. 3d,f). Pseudotime analysis identified a trajectory from transitional cluster c2 to terminal disease clusters c3 and c4 (Fig. 3c). These clusters showed reduced homeostatic markers (*P2ry12*, *Cx3cr1*, *Gpr34*, *Sall1* and *Siglech*) and increased activation genes^27^ (*Apoe*, *Ctsb*, *Lyst*, *Csf1*, *Tyrobp*, *B2m*, *Spp1* or *Cybb*) (Extended Data Fig. 3d). Clusters c2 and c3 shared upregulation of *Igf1*, *Apobec1* and *Flt1* (Fig. 3d,e,g). Gene ontology (GO) analysis linked c2 to cytokine-related terms, c3 to autophagy/phagocytosis pathways and c4 to interferon responses (Fig. 3d,f). Compared with other neurodegeneration models (Fig. 1a), *Hexb*-deficient microglia shared general patterns but aligned most closely with clusters 11 and 12 from 5xFAD and APP23 mice (Extended Data Fig. 3g–k). Together, loss of *Hexb* induces a progressive microglial activation trajectory towards a disease-specific, highly dysfunctional state characterized by altered immune, autophagic and phagocytic profiles.

## A spatiotemporal lipid map

**Fig. 4:**
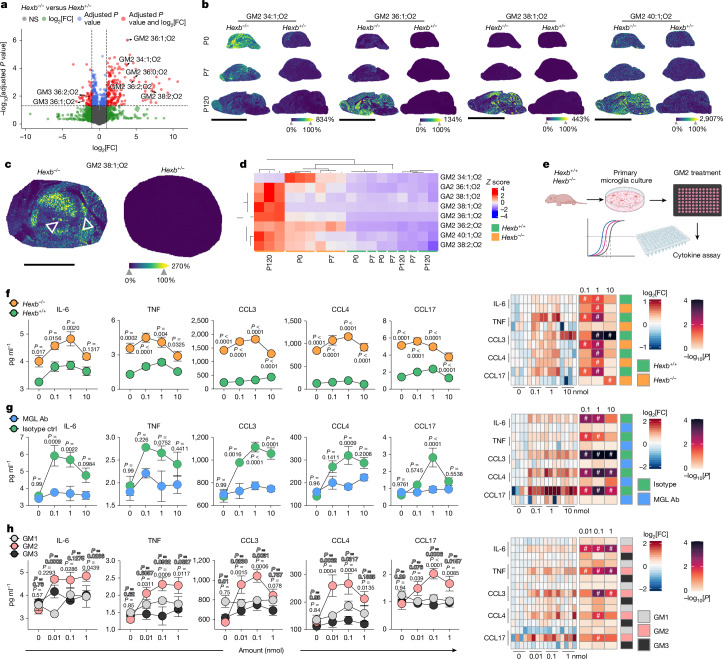
Absence of *Hexb* results in characteristic temporospatial GM2 accumulation, which induces microglial production of proinflammatory cytokines through MGL2. **a**, Volcano plot indicating the differentially regulated lipids between *Hexb*^*−/−*^ (*n* = 6) and *Hexb*^*+/−*^ (*n* = 3) mice measured by untargeted lipidomics (liquid chromatography–mass spectrometry) at P120. Two-tailed Welch’s *t*-test was used for statistical testing. **b**, Spatial MALDI MSI on *Hexb*^*−/−*^ and *Hexb*^*+/−*^ brains at P0 (upper row), P7 (middle row) and P120 (bottom row). For each indicated ganglioside, ion images representative for three biological replicates are shown. Colour scale represents a visual map of the intensities (in arbitrary units) of the ion images. **c**, MALDI MSI on the thalamus of *Hexb*^*−/−*^ and *Hexb*^*+/−*^ mice at P120. For each indicated ganglioside, ion images representative for three biological replicates are shown. Colour scale represents a visual map of the intensities (in arbitrary units) of the ion images. Triangles points to the centromedian and the parafascicular nucleus. **d**, Hierarchical clustering of selected gangliosides in the indicated CNS specimen. Colour scale indicates the *Z*-score. **e**, Experimental scheme. **f**–**h**, Left, absolute cytokine and chemokine levels in the supernatant after culturing primary microglia upon overnight ganglioside stimulation. Data are shown as mean ± s.e.m. from four independent replicates. Two-way ANOVA followed by Sidak’s multiple comparison test was used for statistical testing. Right, log_2_[FC] values (colour scale) are shown relative to the unstimulated condition within each genotype. Statistical significance was assessed using one-way ANOVA with Dunnett’s correction. –log_10_[*P*] is encoded in colour intensity (heat map), and cells marked with # indicate *P* < 0.05. **f**–**h**, A comparison of *Hexb*^*−/−*^ and *Hexb*^*+/+*^ microglia (**f**), the effect of MGL blockade (**g**) (MGL antibody versus isotype control (ctrl)) and responses to different gangliosides (**h**) (GM1, GM2, GM3). Scale bars, 9 mm (**b**), 2 mm (**c**). Illustrations in **e** were created using BioRender. Frosch, M. (2025) https://BioRender.com/kdrwxwm. Source data

**Extended Data Fig. 4:**
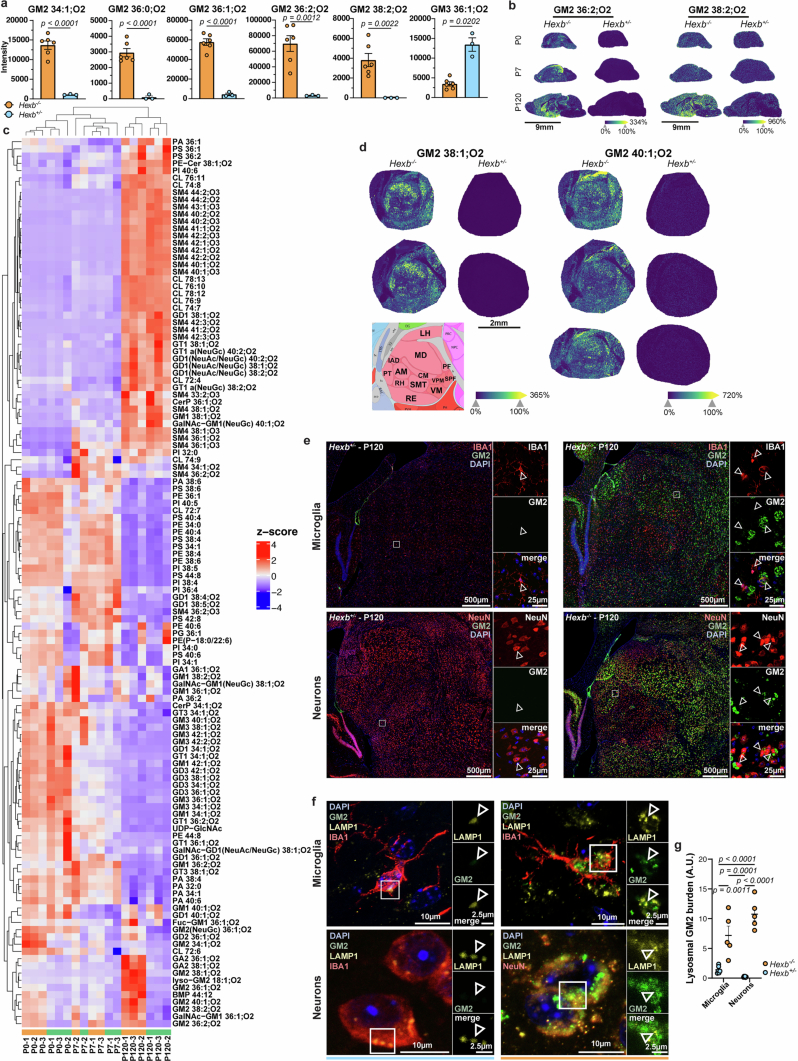
Analysis of ganglioside storage in a temporospatial manner. A) Bar graphs depicting dysregulated ganglioside deposition in brain homogenates of *Hexb*^*−/−*^ (n = 6) and *Hexb*^*+/−*^ mice (n = 3) at 120 days of age measured by untargeted lipidomics (LC-MS). B) Spatial MALDI mass spectrometry imaging (MALDI-MSI) on *Hexb*^*−/−*^ and *Hexb*^*+/−*^ brains at P0 (upper row), P7 (mid row), and P120 (bottom row). For each indicated ganglioside, ion images representative for three biological replicates are shown. Color scale represents a visual map of the intensities (in arbitrary units) of the ion images. C) Heatmap showing all differentially regulated and annotated lipids in the brain of P0, P7, and P120 *Hexb*^*−/−*^ (n = 3) and *Hexb*^*+/+*^ (n = 3) control mice. Color scale indicates the z-score. D) MALDI MSI on the thalamus of *Hexb*^*−/−*^ and *Hexb*^*+/−*^ mice at P120. For each indicated ganglioside, ion images representative for three biological replicates are shown. Color scale represents a visual map of the intensities (in arbitrary units) of the ion images. E) Representative immunofluorescence images of GM2 storage in neurons and microglia in *Hexb*^*−/−*^ mice. Triangles point to GM2^+^IBA1^+^ microglia or GM2^+^NeuN^+^ neurons. F) Representative immunofluorescence images of GM2 storage (green) in lysosomes (LAMP1^+^, yellow) in microglia (IBA1^+^, red, top panel) or neurons (NeuN^+^, red, bottom panel), respectively. Orange color indicates *Hexb*^*−/−*^ mice and blue color *Hexb*^*+/−*^ mice. G) Quantification of lysosomal GM2, shown as the integrated fluorescence intensity of GM2 signal within LAMP1^+^ lysosomal areas, normalized to the cell area defined by IBA1^+^ (microglia) and NeuN^+^ (neurons) signals, respectively. Each symbol indicates one mouse. Data shown as mean ± s.e.m. Statistical analyses: Unpaired Student’s t-test comparisons were used for statistical testing (A); two-way ANOVA followed by Tukey’s test for correcting multiple comparisons was used for statistical testing (G). Source data

To assess the impact of *Hexb* deficiency on CNS lipid composition, we performed untargeted lipidomics on cortical homogenates from *Hexb*^*−/−*^ and *Hexb*^*+/−*^ mice. Among 5,825 identified lipids, 196 were significantly upregulated and 59 downregulated in *Hexb*^*−/−*^ brains (Fig. 4a). Importantly, multiple GM2 species were markedly increased, whereas their Hex-derived products, GM3 gangliosides, were strongly decreased (Extended Data Fig. 4a). To map spatial ganglioside distribution, we applied untargeted matrix-assisted laser desorption/ionization (MALDI) mass spectrometry imaging (MSI)^28^ to brain sections at multiple time points. Several physiologically regulated lipid species varied with age (for example, cardiolipins, sulfatides (SM4), gangliosides (GT1, GR3, GD1, GD3)) but did not differ between genotypes (Extended Data Fig. 4c). By contrast, GM2 accumulation was already detectable at P0 in knockout brains (Fig. 4b,d and Extended Data Fig. 4b). Notably, GM2 molecules with shorter fatty acyl chains (for example, d34:1) predominantly accumulated in neonates, whereas GM2 molecules with longer fatty acyl chains (for example, 38:2) were enriched at P120 (Fig. 4b,d). This shift was especially pronounced in the thalamus, which showed prominent GM2 accumulation at P120 (Fig. 4c and Extended Data Fig. 4d). Within the thalamus, GM2 localized to specific nuclei—the centromedian and parafascicular, key sources of thalamostriatal projections involved in motor control^29^ (Fig. 4c). Beyond GM2, pathologically elevated lipids included GA2, asialo-GM2 and bis(monoacylglycero) phosphate (BMP) 44:12 (Supplementary Fig. 2). Immunofluorescence imaging confirmed GM2 storage in neurons and microglia, but not astrocytes and oligodendrocytes (Extended Data Fig. 4e). Both microglia and neurons exhibited substantial lysosomal ganglioside burden, with neurons tending towards higher levels (Extended Data Fig. 4f,g). Altogether, our spatial ganglioside map revealed a distinct diseases-associated lipid profile driven by *Hexb* loss with regionally enriched GM2 accumulation, especially in the thalamus.

## GM2 activates microglia by MGL2 engagement

**Extended Data Fig. 5:**
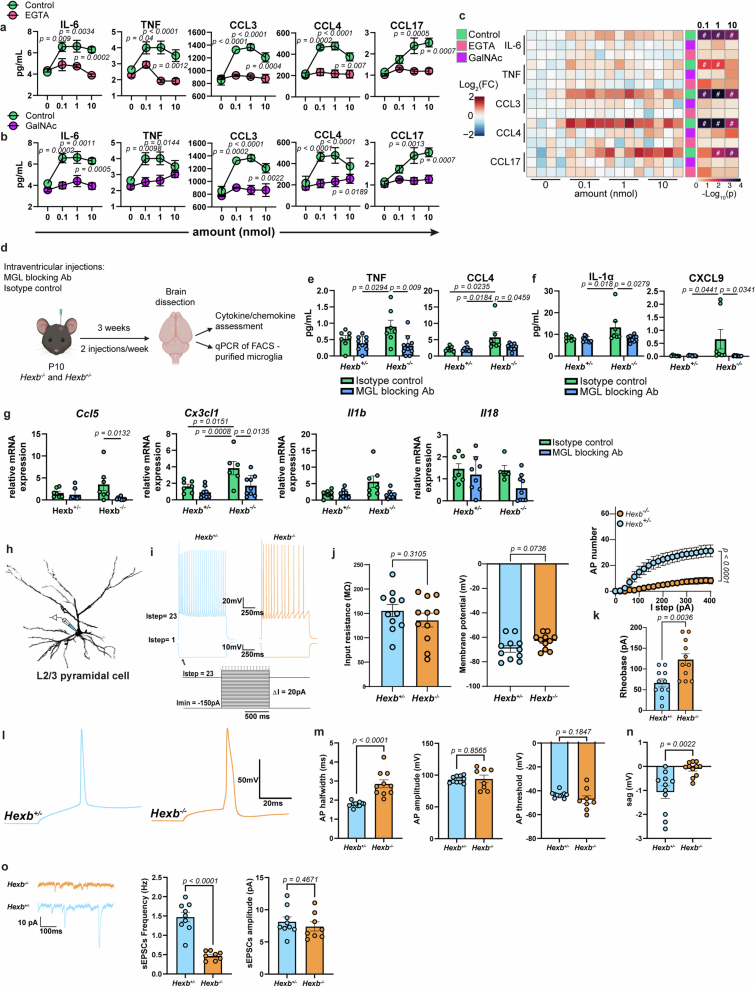
In vitro and in vivo microglial responses to gangliosides and cortical dysfunction in *Hexb*-deficient mice. A-B) Cytokines and chemokine concentrations in supernatants from primary wildtype microglia cultured with the indicated amount of GM2, with/without EGTA or GalNAc. Data from 4 technical replicates. C) Fold changes of indicated cytokines normalized to the unstimulated condition (based on A and B) D) Schematic of in vivo experimental design. E-F) Cytokines/chemokine levels in brain lysates following ICV antibody injections. G) Quantitative PCR analysis of indicated target genes. H) Experimental setup. I) Representative traces (top) and current-clamp recording protocol (bottom). Note fewer action potentials (APs) and absence of voltage sag (arrow) in *Hexb*^*−/−*^ compared to *Hexb*^*+/−*^ mice. J) No difference in basic neuronal attributes between the genotypes (*Hexb*^*+/−*^: 4 animals, 11 neurons; *Hexb*^*−/−*^:4 animals, 11 neurons). K) *Hexb*^*−/−*^ mice display reduced AP firing in response to depolarizing current injections across the entire range tested (left), along with an increase in rheobase, the amount of current required to elicit an AP (right, *Hexb*^*+/−*^-: 4 animals, 11 neurons; *Hexb*^*−/−*^: 4 animals, 11 neurons). L) Representative traces of individual APs. M) *Hexb*^*−/−*^ mice show increased AP halfwidth, whereas AP amplitude and threshold are similar between the genotypes (*Hexb*^*+/−*^: 4 animals, 10 neurons; *Hexb*^*−/−*^:4 animals, 10/8/8 neurons respectively) N) Voltage sag during hyperpolarization (arrow in panel b) is reduced in *Hexb*^*−/−*^ mice (*Hexb*^*+/−*^: 4 animals, 11 neurons; *Hexb*^*−/−*^:4 animals, 11 neurons). O) Voltage-clamp recordings of spontaneous excitatory postsynaptic currents (sEPSCs) show. robust reduction of sEPSC frequency in *Hexb*^*−/−*^ mice. sEPSC amplitude is similar in both genotypes (right, *Hexb*^*+/−*^-: 4 animals, 9 neurons; *Hexb*^*−/−*^:4 animals, 8 neurons). Data shown as mean ± s.e.m. Statistical analyses: Two-way ANOVA followed by Sidak’s multiple comparison test (A-B,E-G); One-way ANOVA followed by Dunnett’s test (C); two-tailed Student’s t-test (I-K, M-O); two tailed Mann-Whitney (K). For I-O, dots correspond to individual neurons. Illustrations in **d** and **h** were created using BioRender (https://biorender.com). Source data

Having identified GM2 as the most dysregulated lipid in *Hexb*^*−/−*^ brains and its spatial overlap with microgliosis, we examined its direct effects on microglia. Primary microglia from *Hexb*^*−*/*−*^ and *Hexb*^+/+^ control mice were plated into GM2-coated 96-well plates, and cytokine and chemokine secretion was measured (Fig. 4e). GM2 induced a dose-dependent release of IL-6, TNF, CCL3, CCL4 and CCL17, with higher levels in *Hexb*^*−/−*^ cells, probably owing to pre-activation from chronic ganglioside exposure (Fig. 4f and Supplementary Fig. 3). To dissect the mechanism, we focused on MGL2, a C-type lectin receptor expressed on dendritic cells, macrophages and microglia that specifically binds to terminal *N*-acetylgalactosamine (GalNAc) present on GM2 (ref. ^30^). Although MGL2 is known for endocytosis^31^, we tested whether it also mediates GM2-driven microglial activation. Indeed, microglia pretreated with MGL2-blocking antibody no longer responded to GM2 with cytokine release, whereas isotype-treated cells did (Fig. 4g and Supplementary Fig. 3). To rule out non-specific antibody effects, we used EGTA to chelate extracellular calcium, essential for C-type lectin function, or with GalNAc to competitively inhibit MGL2 binding, both of which suppressed cytokine responses, confirming the specificity of MGL2–GM2 signalling (Extended Data Fig. 5a–c). Furthermore, GM1 and GM3—lacking an accessible or present GalNAc—did not induce cytokines^32^ (Fig. 4h and Supplementary Fig. 3).

To validate our findings in vivo, we administered MGL2-blocking antibody by means of intracerebroventricular injections to *Hexb*^*−/−*^ and *Hexb*^*+/−*^ mice from P10 for 3 weeks (Extended Data Fig. 5d). Injected *Hexb*^*−/−*^ brains showed a significant reduction in TNF and CCL4, along with decreased IL-1α and CXCL9 levels (Extended Data Fig. 5e,f). Fluorescence-activated cell sorting (FACS)-purified microglia had decreased *Ccl5* and *Cx3cl1* mRNA and trends towards reduced *Il1b* and *Il18* mRNA expression (Extended Data Fig. 5g). Together, these findings demonstrate that GM2 acts as a specific microglial activator through MGL2, both in vitro and in vivo.

## *Hexb* loss impairs neuronal excitability

Having defined the mechanism of GM2-induced microglial activation, we next investigated whether *Hexb* deficiency and subsequent GM2 accumulation impair neuronal signalling. Thus, we performed electrical recordings from motor cortex layer 2/3 pyramidal neurons in acute slices (Extended Data Fig. 5h,i). Although input resistance and membrane potential were unchanged (Extended Data Fig. 5j), *Hexb*^*−/−*^ neurons fired significantly fewer action potentials during depolarizing current steps, indicating reduced excitability (Extended Data Fig. 5i,k). In addition, changes in action potential halfwidth (Extended Data Fig. 5l,m) and voltage sag during hyperpolarization (Extended Data Fig. 5n) suggested dysregulation of the underlying conductances. At the network level, *Hexb*^*−/−*^ neurons showed reduced frequency of synaptic inputs, reflecting impaired glutamatergic connectivity (Extended Data Fig. 5o). Collectively, these data reveal robust deficits in neuron-autonomous and circuit function upon *Hexb* deficiency and GM2 accumulation.

## Microglial and neuronal *Hexb* drive disease

**Fig. 5:**
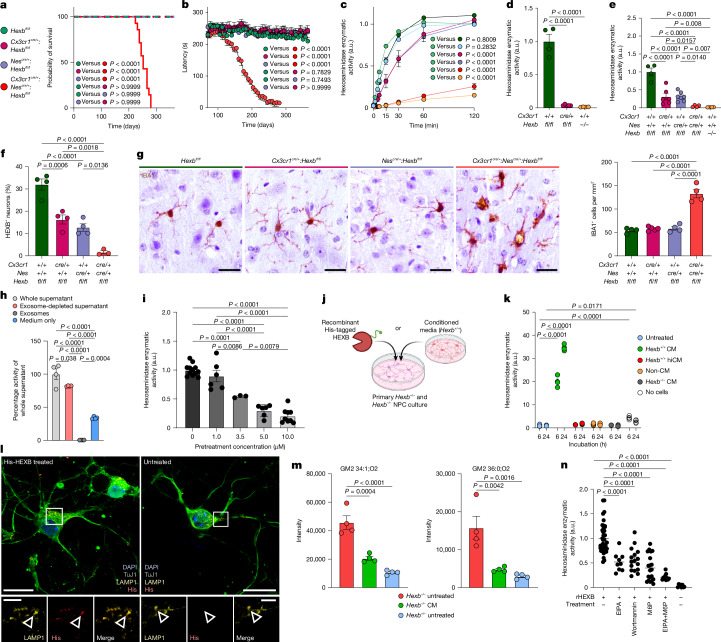
Joint microglial secretion and neuronal uptake of Hex sustains CNS homeostasis and prevents Sandhoff disease. **a**,**b**, Kaplan–Meier survival curves (**a**) and rotarod performance (**b**) of *Hexb*^*fl/fl*^ (*n* = 15), *Cx3cr1*^*cre/+*^:*Hexb*^*fl/fl*^ (*n* = 15), *Nes*^*cre/+*^:*Hexb*^*fl/fl*^ (*n* = 15) and *Cx3cr1*^*cre/+*^:*Nes*^*cre/+*^:*Hexb*^*fl/fl*^ (*n* = 14). **c**, Hex activity in whole-brain homogenates at indicated times (*n* = 4). a.u., arbitrary units. **d**,**e**, Activity in microglia (**d**) and neurons (**e**): *Hexb*^*fl/fl*^ (*n* = 4), *Cx3cr1*^*cre/+*^:*Hexb*^*fl/fl*^ (*n* = 5/6), *Nes*^*cre/+*^:*Hexb*^*fl/fl*^ (*n* = 6), *Cx3cr1*^*cre/+*^:*Nes*^*cre/+*^:*Hexb*^*fl/fl*^ (*n* = 4), *Hexb*^*−/−*^ (*n* = 4). **f**, Quantification of HEXB^+^ cortical neurons: *Hexb*^*fl/fl*^ (*n* = 4), *Cx3cr1*^*cre/+*^:*Hexb*^*fl/fl*^ (*n* = 4), *Nes*^*cre/+*^:*Hexb*^*fl/fl*^ (*n* = 4) and *Cx3cr1*^*cre/+*^:*Nes*^*cre/+*^:*Hexb*^*fl/fl*^ (*n* = 3). **g**, IBA1^+^ microglia in the thalamus at P245: *Hexb*^*fl/fl*^ (*n* = 4), *Cx3cr1*^*cre/+*^:*Hexb*^*fl/fl*^ (*n* = 5), *Nes*^*cre/+*^:*Hexb*^*fl/fl*^ (*n* = 4) and *Cx3cr1*^*cre/+*^:*Nes*^*cre/+*^:*Hexb*^*fl/fl*^ (*n* = 4). **h**, Hex activity in primary wild-type microglial supernatants after 4 h (*n* = 4). **i**, The same, after golgicide A pretreatment: 0 µM: *n* = 12; 1 µM: *n* = 6; 3.5 µM: *n* = 3; 5 µM: *n* = 6; 10 µM: *n* = 9. **j**, Experimental scheme. **k**, Activity in *Hexb*^*−/−*^ NPCs treated with conditioned media (CM), heat-inactivated CM (hiCM), unconditioned media (non-CM) or CM-only wells (*n* = 4 each). **l**, Immunocytochemistry of *Hexb*^*+/−*^ NPCs (TuJ1^+^) shows lysosomal (LAMP1^+^) localization of His-tagged Hex. **m**, GM2 levels in CM-treated NPCs (*n* = 4). **n**, Activity in *Hexb*^*−/−*^ NPCs co-treated with His-tagged Hex and endocytosis inhibitors. Recombinant HEXB (rHEXB) only: *n* = 38; EIPA: *n* = 9; Wortmannin: *n* = 16; M6P: *n* = 20; EIPA + M6P: *n* = 11; untreated: *n* = 8. Data are shown as mean ± s.e.m. Statistical analyses: log-rank test (**a**); one-way ANOVA with Tukey’s post hoc test (**b**,**d**–**i**,**k**,**m**,**n**); two-way ANOVA with Dunnett’s test (**c**); each symbol in **h**, **i**, **k**, **m** and **n** represents a technical replicate. Scale bars, 20 µm (**g**), 10 µm (**l**, top panels), 2.5 µm (**l**, bottom panels). Illustrations in **j** were created using BioRender. Frosch, M. (2025) https://BioRender.com/cedrdxp. Source data

**Extended Data Fig. 6:**
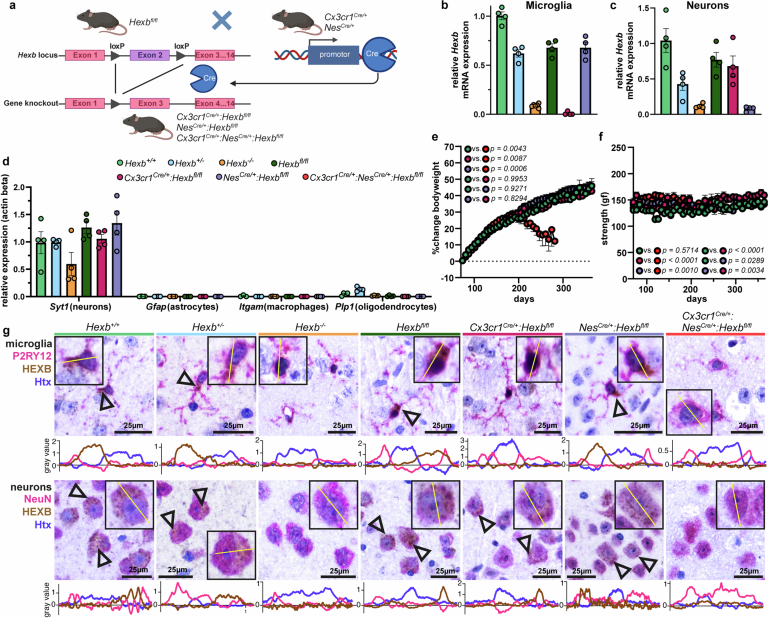
Microglial and neuronal *Hexb* jointly drive Sandhoff disease pathogenesis. A) Schematic overview of genetic targeting. *Hexb*^*fl/fl*^ were obtained by crossing the *B6.Hexb*^*tm1a(EUCOMM)Hmgu*^*/H* line with a FLP deleter. Newly generated *Hexb*^*fl/fl*^ mice with LoxP sites (black triangles) around exon 2 were crossed to *Cx3cr1*^*Cre/+*^ and *Nes*^*Cre/+*^ mice. B) B)-C) Relative *Hexb* gene expression in FACS-sorted microglia (B) and bead-purified neurons (C) among different genotypes measured by qPCR. D) Relative *Syt1* (neuronal marker gene), *Gfap* (astocytic marker gene), *Itgam* (microglia marker gene), and *Plp1* (oligondendroglial marker gene) gene expression in bead-purified neurons among different genotypes measured by qPCR. E-F) Development of bodyweight (E) and muscle strength (F) for *Hexb*^*fl/fl*^ (n = 15), *Cx3cr1*^*Cre/+*^:*Hexb*^*fl/fl*^ (n = 15), *Nes*^*Cre/+*^:*Hexb*^*fl/fl*^ (n = 15), and *Cx3cr1*^*Cre/+*^:*Nes*^*Cre/+*^:*Hexb*^*fl/fl*^ (n = 14) mice. G) Representative immunohistochemical images of brain sections from the indicated genotypes. Top row: Microglia immunostained for P2RY12 (red), HEXB (brown), and counterstained with hematoxylin (Htx, blue). Bottom row: Neurons stained for NeuN (red), HEXB (brown), and Htx (blue). Insets show higher magnification views with yellow lines indicating the paths used for intensity profile quantification below. Triangles highlight intracellular HEXB-double positive structures. Graphs display greyscale intensity profiles of the deconvoluted staining signals along the yellow lines (red = P2RY12 or NeuN, brown = HEXB, blue = Htx). Data shown as mean ± s.e.m. Statistical analyses: one-way ANOVA with Tukey’s post hoc test (E-F). Illustrations in **a** were created using BioRender (https://biorender.com). Source data

Until now, limited cell-type-specific targeting tools hindered identification of disease-driving cell types in Sandhoff disease. Although loss of neuronal Hex activity is believed to drive GM2 accumulation and pathology^33^, the high expression of *Hexb* in microglia (Fig. 1c–g) suggests a further role in disease progression. To dissect cell-specific contributions, we flanked exon 2 of the *Hexb* gene by loxP sites and generated *Hexb*^*fl/fl*^ mice (Extended Data Fig. 6a). These were crossbred with *Nes*^*cre/+*^ (targeting neuroectodermal cells^34^, including neurons) and *Cx3cr1*^*cre/+*^ (targeting myeloid cells^6,35^, including microglia). Each line showed efficient *Hexb* deletion (Extended Data Fig. 6b–d). Surprisingly, neither *Nes*^*cre/+*^:*Hexb*^*fl/fl*^ nor *Cx3cr1*^*cre/+*^:*Hexb*^*fl/fl*^ mice recapitulated the phenotype observed in constitutive *Hexb*^*−*/*−*^ mice, and survival, motor function and body weight remained normal (Fig. 5a,b and Extended Data Fig. 6e,f). In both single knockouts, total brain Hex activity was only slightly reduced (Fig. 5c), and neurons retained enzymatic activity and HEXB-positive structures, pointing to a redundant role of microglial or neuronal *Hexb* expression (Fig. 5d–f and Extended Data Fig. 6g). Notably, only double knockout *Cx3cr1*^*cre/+*^:*Nes*^*cre/+*^:*Hexb*^*fl/fl*^ mice recapitulated the disease with late-onset motor symptoms, weight loss and premature death (Fig. 5a,b and Extended Data Fig. 6e). These mice showed marked reduction of Hex activity and massive loss of HEXB^+^ neurons (Fig. 5c,e,f and Extended Data Fig. 6g). Microglia also exhibited abnormal morphology and increased cell numbers (Fig. 5g). Overall, only a combined deficiency of *Hexb* in the neuroectodermal and myeloid compartments is sufficient to induce Sandhoff disease.

## Microglia aid neuronal lysosomal function

**Extended Data Fig. 7:**
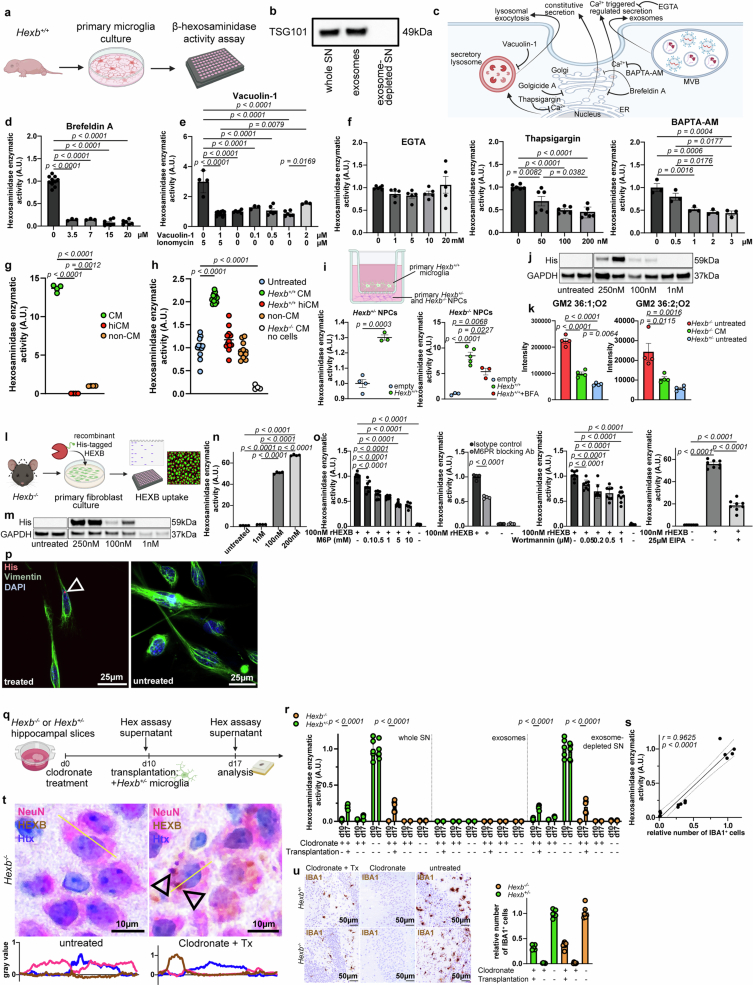
Microglial Hex secretion and uptake by other cell types. A) Experimental scheme of microglia culture and Hex activity measurement in cell culture supernatants. B) Immunoblot of culture supernatant and exosome isolates for TSG101 (exosomal marker). C) Diagram of major protein secretion pathways and their molecular inhibitors. ER: endoplasmic reticulum, EGTA: ethylene glycol-bis(β-aminoethyl ether)-N,N,N’,N’-tetraacetic acid, MVB: multivesicular body. D) D)-F) Hex activity in microglial supernatants treated with indicated concentrations of Brefeldin A (D), Vacuolin-1 and/or Ionomycin (E), or EGTA, Thapsigargin, and BAPTA-AM (F). G) Enzyme activity in conditioned media (CM), heat-inactivated CM (hiCM), and unconditioned media (non-CM). H) Hex activity in lysates of *Hexb*^*+/−*^ NPCs treated with CM, hiCM, non-CM, or CM added to wells without cells (“no cells”) for 24 h. I) Top: Experimental setup using transwell inserts. Bottom: Ηεξ activity in lysates of *Hexb*^*−/−*^ and *Hexb*^*+/−*^ NPCs after co-culture with *Hexb*^*+/+*^ microglia ± Brefeldin A (BFA) J) Immunoblots of NPC lysates after 6 h treatment with recombinant Hex-His. K) GM2 levels in NPC lysates after CM treatment. L) Experimental scheme for primary *Hexb*^*−/−*^ fibroblast culture. M) Immunoblots of fibroblast lysates after 6 h treatment with recombinant Hex-His. N-O) Enzyme activity in fibroblast lysates ± recombinant enzyme and following pretreatment with mannose-6-phosphate (M6P), M6P receptor (M6PR) antibody, wortmannin, or EIPA. P) Immunocytochemistry if His-tagged enzyme in *Hexb*^*−/−*^ fibroblasts (vimentin^+^, His^+^). Triangles point to intracellular His^+^ inclusions. Q) Schematic of chimeric organotypic hippocampal slice cultures (OHSCs). R) Hex activity in OHSC supernatants, exosomes, and exosome-depleted fractions from *Hexb*^*+/−*^ and *Hexb*^*−/−*^ slices ± clodronate treatment and microglia transplantation. S) Correlation of enzyme activity with microglia density. (Spearman r, p values from two-tailed t-test). T) Immunohistochemistry for NeuN (red) and HEXB (brown) counterstained with hematoxylin (Htx) in microglia-transplanted *Hexb*^*−/−*^ slices at d17. Graphs display greyscale intensity profiles. U) Immunohistochemistry and quantification of IBA1^+^ microglia in OHSCs at d17. Data shown as mean ± s.e.m. Statistical analyses: one-way ANOVA with Tukey’s post hoc test (D-I,K,O); two-way ANOVA with Sidak’s test (O,R). Illustrations in **a**, **c**, i, **l** and **q** were created using BioRender (https://biorender.com). Source data

As neither microglia nor neurons alone induce disease, we hypothesized a compensatory mechanism for GM2 turnover involving microglial enzyme release. To test this, we cultured *Hexb*^*+/+*^ microglia and measured Hex activity in the supernatant (Extended Data Fig. 7a). Activity was significantly higher than in medium-only controls and localized to the exosome-depleted fraction, indicating secretion of free Hex (Fig. 5h and Extended Data Fig. 7b). To determine the secretion pathway, we treated microglia with various inhibitors (Fig. 5i and Extended Data Fig. 7c–f). Among them, only golgicide A and brefeldin A significantly and dose-dependently blocked microglial Hex secretion (Fig. 5i and Extended Data Fig. 7d), suggesting release by means of the classical secretory pathway. Thapsigargin and BAPTA-AM also reduced enzyme secretion, implicating intracellular Ca^2+^ in sustained secretion, whereas vacuolin-1 and EGTA had no effect (Extended Data Fig. 7e,f).

To assess neuronal uptake, *Hexb*^*−/−*^ and *Hexb*^*+/−*^ neural progenitor cells (NPCs) were treated with His-tagged recombinant Hex or conditioned media from primary wild-type microglia (Fig. 5j and Extended Data Fig. 7g). Conditioned media induced a time-dependent increase in enzymatic activity in NPCs (Fig. 5k and Extended Data Fig. 7h), corroborated by Transwell co-culture experiments, confirming enzyme transfer is independent of direct cell contact (Extended Data Fig. 7i). Western blotting confirmed Hex uptake by NPCs (Extended Data Fig. 7j), and immunofluorescence imaging validated lysosomal localization of internalized enzyme (Fig. 5l). Notably, conditioned media-treated *Hexb*^*−/−*^ neurons stored less GM2, confirming the functional contribution of microglia-derived Hex to neuronal ganglioside degradation (Fig. 5m and Extended Data Fig. 7k).

To explore uptake mechanisms, we co-treated *Hexb*^*−/−*^ NPCs with inhibitors: 5-(*N*-ethyl-*N*-isopropyl) amiloride (EIPA) and Wortmannin (micropinocytosis), or mannose-6-phosphate (M6P receptor blockade). All significantly reduced Hex uptake (Fig. 5n), suggesting two parallel routes: macropinocytosis and M6PR-mediated endocytosis. These findings were further validated in *Hexb*^*−/−*^ fibroblasts (Extended Data Fig. 7l–p).

To test this mechanism in tissue, we depleted microglia in organotypic hippocampal *Hexb*^*−/−*^ or *Hexb*^*+/−*^ slices using clodronate, then reintroduced *Hexb*-competent microglia (Extended Data Fig. 7q). Upon depletion, the Hex activity completely dropped, and strongly correlated with IBA1^+^ microglia numbers at day 17, clearly pointing towards microglia as the main source of secreted Hex within the mouse CNS (Extended Data Fig. 7r,s,u). At day 17, we observed HEXB in neurons of chimeric *Hexb*^*−/−*^ slices, confirming microglial supply (Extended Data Fig. 7t). Together, microglia constitutively secrete Hex through the classical secretory Golgi-dependent pathway. The enzyme is endocytosed by neurons through macropinocytosis and M6P receptor-mediated endocytosis, is trafficked to lysosomes and facilitates GM2 degradation.

## *Hexb* in MLCs prevents neurodegeneration

**Fig. 6:**
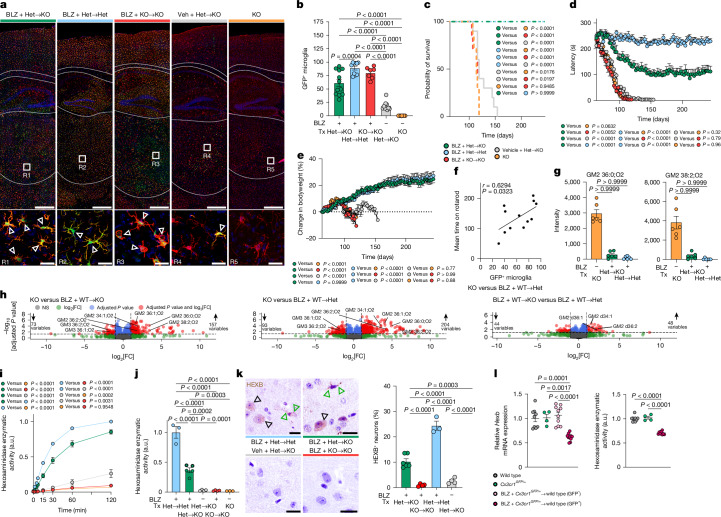
Expression of *Hexb* by bone-marrow-derived MLCs rescues lethal CNS phenotype and restores brain homeostasis. **a**, Representative immunofluorescence images at P245 showing IBA1 (red), GFP and DAPI (4,6-diamidino-2-phenylindole; blue). Triangles indicate IBA1^+^GFP^+^-replaced microglia. **b–e**, Microglia GFP expression by flow cytometry (**b**), Kaplan–Meier survival analysis (**c**), rotarod performance (**d**) and body weight monitoring (**e**). BLZ + Het → KO (dark green, *n* = 12), BLZ + Het → Het (blue, *n* = 10), BLZ + KO → KO (red, *n* = 7), vehicle + Het → KO (grey, *n* = 10) and KO (orange, *n* = 7) were analysed. Het, *Hexb*^+/−^; KO, knockout, *Hexb*^−/−^; Tx, transplantation. **f**, Correlation between motor function and microglia replacement efficiency. Spearman’s *r* and two-tailed *t*-test *P* values are indicated. **g**, Bar graphs depicting GM2 ganglioside deposition in brain homogenates at P120 (*n* = 6 per group). **h**, Volcano plots of differentially regulated lipids at P120 (*n* = 6 per group). **i**, Hex activity in whole-brain homogenates (*n* = 4 per group) measured at indicated times. **j**, Hex activity in neurons at P120 (BLZ + Het → KO (*n* = 5), BLZ + KO → KO (*n* = 4), BLZ + Het → Het (*n* = 3), vehicle + Het → KO (*n* = 4) and KO (*n* = 3)). **k**, Left, HEXB immunohistochemistry in the cortex at P120. Arrowheads mark neuronal (black) and microglial (green) HEXB^+^ cells. Right, quantification of HEXB^+^ neurons (BLZ + Het → KO (*n* = 5), BLZ + KO → KO (*n* = 4), BLZ + Het → Het (*n* = 3) and vehicle + Het → KO (*n* = 4)). **l**, Relative *Hexb* gene expression and Hex activity in microglia from wild-type mice, *Cx3cr1*^*GFP/+*^ mice and microglia-replaced mice, separated by GFP status. Data are shown as mean ± s.e.m. Statistical analyses: one-way ANOVA with Tukey’s post hoc test (**b**,**d**,**e**,**h**,**j**–**l**); log-rank test (**c**); two-tailed Welch’s *t*-test (**g**); two-way ANOVA with Tukey’s post hoc test (**i**). Scale bars, 500 µm (**a**, top panels), 25 µm (**a**, bottom panels, **k**). Source data

**Extended Data Fig. 8:**
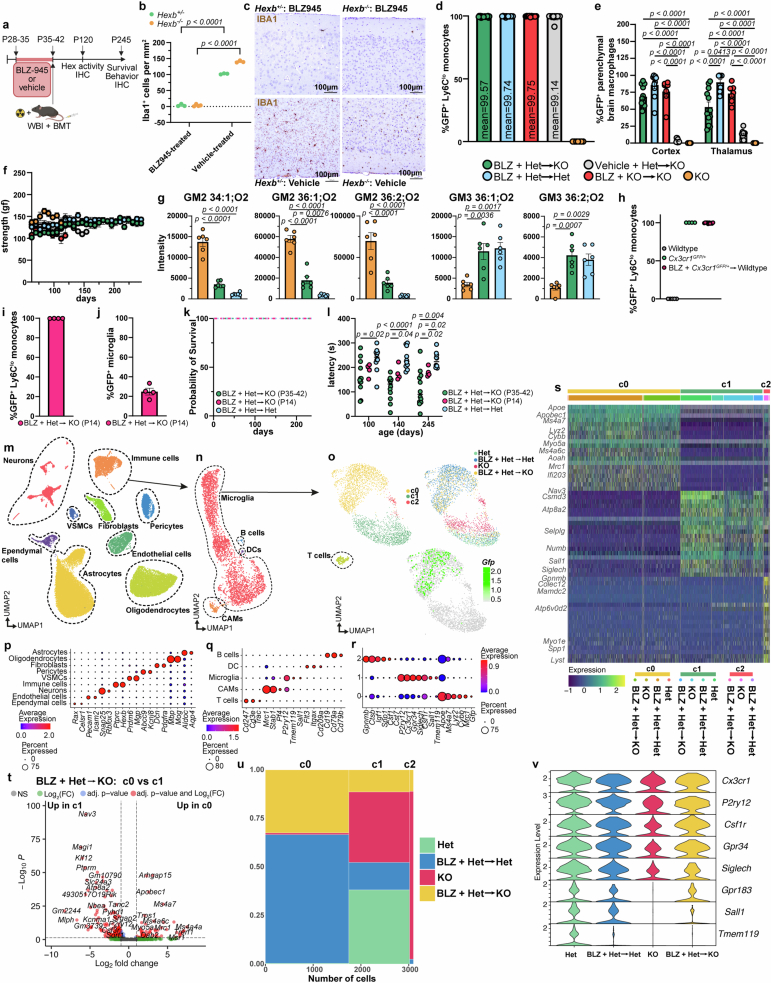
Microglial *Hexb* expression ensures CNS homeostasis. A) Experimental scheme for microglia replacement. WBI: whole body irradiation. BMT: bone marrow transplantation. B-C) Quantification and immunohistochemistry of cortical IBA1^+^ cells in BLZ945- and vehicle-treated *Hexb*^*+/−*^ and *Hexb*^*−/−*^ mice (n = 3 per group). D) FACS analysis of GFP^+^ Ly6C^lo^ blood monocytes in the transplanted and microglia replaced mice measured by FACS. E) Quantification of %GFP^+^IBA1^+^ parenchymal cells in the cortex and thalamus (replacement efficiency) from Fig. 6a. F) Grip strength assessment in transplanted mice. G) Ganglioside levels in brain homogenates of transplanted *Hexb*^*−/−*^ (n = 6) and *Hexb*^*+/−*^ animals, and untreated *Hexb*^*−/−*^ controls (n = 6). H-I) Bar graph highlighting the amount of GFP^+^Ly6C^lo^ blood monocytes related to Fig. 6l (H) and of neonatally transplanted and microglia replaced mice (I). J) Flow cytometry of GFP expression in microglia of neonatally transplanted mice. K-L) Kaplan–Meier survival curve (K) and rotarod performance (L) of neonatally transplanted mice. BLZ + Het → KO (n = 12), BLZ + Het → Het (n = 10), and neonatally BLZ + Het → KO (n = 4). M-O) UMAP of 44,957 individual nuclei from the thalamus of transplanted and untransplanted *Hexb*^*−/−*^ and *Hexb*^*+/−*^ mice captured by snRNA-seq. P-Q) Marker genes identifying cell types and immune populations. R) Dotplot of homeostatic and disease-associated microglial genes by clusters. S) Heat map of key cluster-specific microglial genes. T) Volcano plot depicting DEGs between c0 and c1 in transplanted *Hexb*^*−/−*^mice. U) Marimekko chart of microglia depicting the proportions of transplanted and untransplanted *Hexb*^*−/−*^ and *Hexb*^*+/−*^ microglia for each cluster. V) Violin plot highlighting the expression of core microglial genes in transplanted and untransplanted *Hexb*^*−/−*^ and *Hexb*^*+/−*^ microglia (c1). Data shown as mean ± s.e.m. Statistical analyses: two-way ANOVA followed by Sidak’s test (B); one-way ANOVA with Tukey’s post hoc test (E,G,L). Illustrations in **a** were created using BioRender (https://biorender.com). Source data

Having shown that microglia can restore neuronal Hex activity ex vivo, we next tested whether microglial replacement could provide therapeutic benefit in vivo. To this end, *Hexb*^*−/−*^ mice underwent microglia depletion by using the CSF1R inhibitor BLZ945, followed by transplantation with *Cx3cr1*^*GFP/+*^:*Hexb*^*+/−*^ bone marrow (Extended Data Fig. 8a–d). Recipient mice showed a high degree of myeloid cell engraftment after P245 (Fig. 6a,b and Extended Data Fig. 8e). Notably, chimeric *Hexb*^*−/−*^ mice displayed normalized survival, mitigated motor symptoms and stable body weight (Fig. 6c–e and Extended Data Fig. 8f). Brain homogenates exhibited restored Hex activity and markedly reduced GM2 accumulation (Fig. 6g,h,i and Extended Data Fig. 8g). Neurons in transplanted animals also regained Hex activity and HEXB immunoreactivity, confirming microglia as a sufficient extrinsic source (Fig. 6j,k). Importantly, without BLZ945-mediated niche depletion, transplantation alone had only negligible effects on disease outcome, indicating the necessity of an empty microglia niche (Fig. 6b–e). Indeed, behavioural improvement significantly correlated with the proportion of engrafted *Hexb*-competent microglia-like cells (MLCs) (Fig. 6f). Of note, donor-derived MLCs exhibited approximately 60% (61.49 ± 2.39%) of *Hexb* mRNA expression and 70% (70.72 ± 0.98%) of Hex enzymatic activity compared with residual endogenous microglia (Fig. 6l and Extended Data Fig. 8h). Moreover, initiating treatment neonatally further enhanced outcomes (Extended Data Fig. 8i–l), collectively underscoring the therapeutic potential of early intervention and genetically enhanced donor cells.

To analyse transcriptional changes, we performed snRNA-seq of thalamic nuclei from transplanted and untransplanted *Hexb*^*−/−*^ and *Hexb*^*+/−*^ mice (Extended Data Fig. 8m–o). Unsupervised clustering identified three major microglia clusters: c1 was enriched for homeostatic genes (*Tmem119*, *P2ry12* and *Sall1*), c2 showed elevated expression of disease-associated genes (*Spp1*, *Gpnmb*, *Ctsb* and *Cst7*) and c0 consisted primarily of donor-derived MLCs with a distinct profile (Extended Data Fig. 8p–t). Host microglia in transplanted *Hexb*^*−/−*^ brains showed reduced c2 occupancy and increased expression of homeostatic genes (Extended Data Fig. 8u,v), suggesting partial transcriptomic normalization through MLC engraftment. In sum, these data strongly suggest that donor-derived MLCs restore lysosomal function, limit GM2 buildup and help re-establish homeostatic microglial states, offering a clinically applicable strategy for Sandhoff disease treatment.

## Shared disease pattern in Sandhoff disease brains

**Fig. 7:**
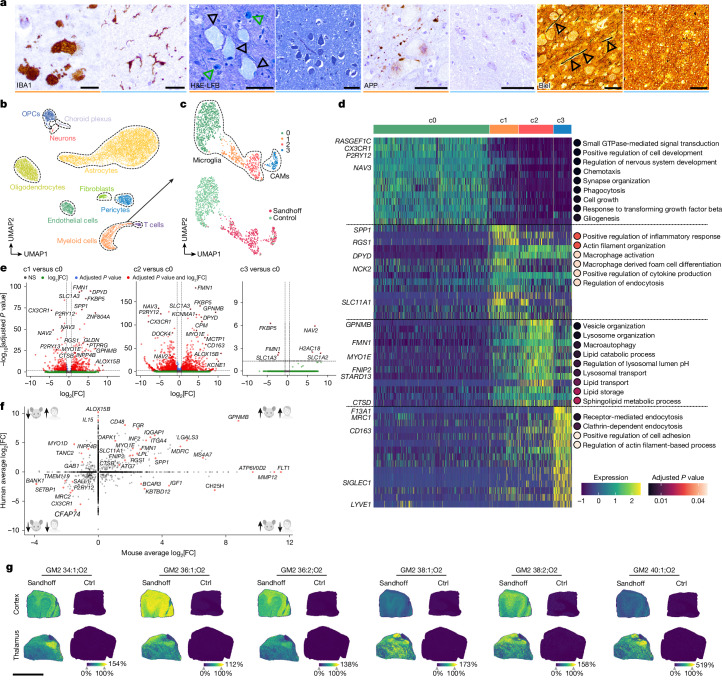
Microglial phenotypes from patients with Sandhoff disease mirror the disease hallmarks observed in *Hexb*^*−/−*^ mice. **a**, Typical immunohistochemical images of thalamic brain sections showing IBA1^+^ (brown) microglia of one postmortem patient with Sandhoff disease. Representative haematoxylin and eosin combined with luxol fast blue (H&E-LFB) stain shows lipid accumulations in thalamic neurons (black arrowheads) and microglial cells (green arrowheads). APP immunohistochemistry displays extracellular deposits in the Sandhoff disease-affected thalamus. Bielschowsky (Biel) stain highlights swollen axons (arrowheads). Orange colour indicates patient with Sandhoff disease, blue unaffected controls. **b**, UMAP visualization of 14,657 individual nuclei from the thalamus of two patients with Sandhoff disease and unaffected controls captured by snRNA-seq. **c**, UMAP visualization of the immune cell subset (without T cells). **d**, Heat map of genes (rows) and dot plots for GO terms associated with each cluster of microglia shown in **c**. Key genes are highlighted. Colours in the heat map correspond to normalized scaled expression. Dot colour indicates adjusted *P* values from over-representation tests with Benjamini–Hochberg correction. **e**, Volcano plots show the DEGs between the indicated microglial clusters. MAST test was used for statistical testing. **f**, Scatter plot depicting the DEGs in mouse and man with selected genes highlighted. Axes indicate expression changes in mouse (*x*) and human (*y*); genes with adjusted *P* < 0.05 are shown. **g**, MALDI MSI on cortex and thalamus of a patient with Sandhoff disease and control shows spatial distribution of gangliosides. Ion images reflect signal intensities (arbitrary units). Scale bars, 20 µm (**a**, IBA1 panels), 50 µm (**a**, other panels), 9 mm (**g**). Illustrations in **f** were created using BioRender. Frosch, M. (2025) https://BioRender.com/4a40uoi.

**Extended Data Fig. 9:**
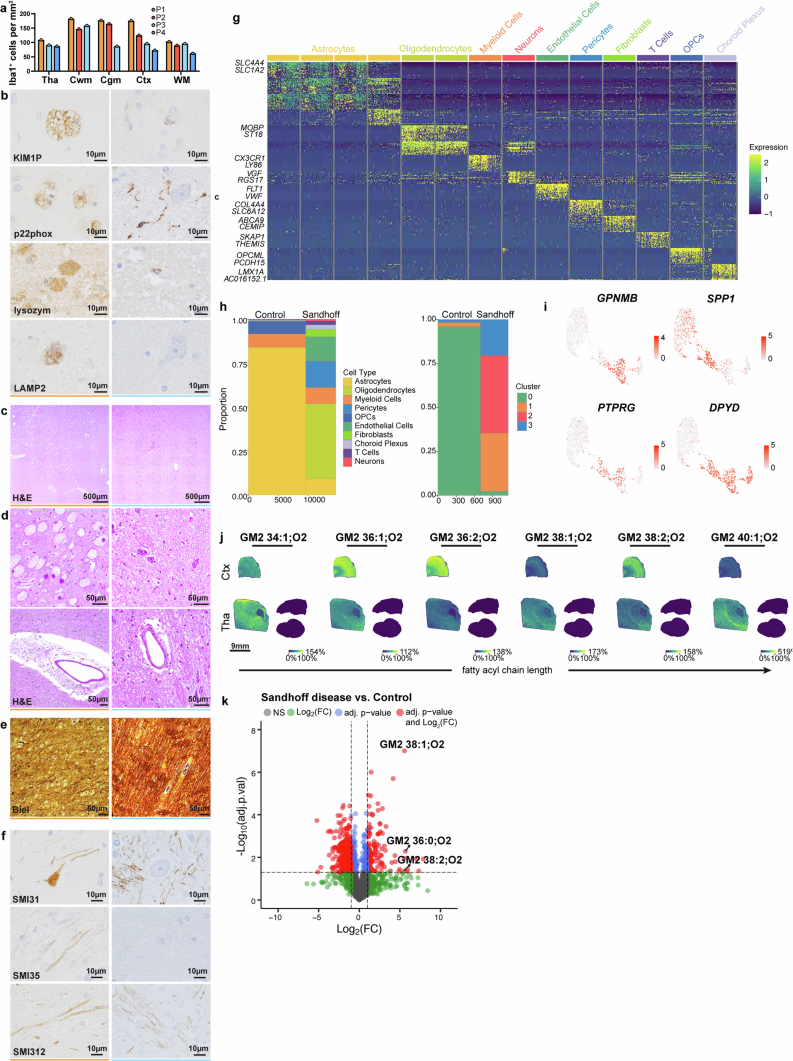
Histological, transcriptional, and lipid changes in Sandhoff disease brains. A) Quantification of IBA1^+^ microglia in postmortem brain tissue of Sandhoff disease patients and unaffected controls. Orange indicates Sandhoff disease, blue unaffected controls. Th: thalamus, Cwm: Cerebellum – white matter, Cgm: Cerebellum – grey matter, Ctx: cortex, WM: subcortical white matter. Each bar represents one patient. B) Immuohistochemistry for phagocytic and lysosomal markers: KIM1P, p22phox, lysozyme, and LAMP2. C) H&E staining thalamic sections of Sandhoff disease patients and healthy controls. D) H&E stains depicting cellular ganglioside deposition and an enlarged hypercellular perivascular space. E) Bielschowsky (Biel) stain highlighting the axonal network. F) Immunostaining for axonal- and neurofilament-associated proteins: SMI31 (phosphorylated neurofilaments), SMI35 (non-phosphorylated neurofilaments), and SMI312 (pan-axonalhighly phosphorlylated neurofilaments marker). Note the abnormal accumulation of SMI31 in perikarya of degenerationg neurons with ganglioside accumulation as well as axonal swellings. G) Heat map featuring the top cell-type-specific marker genes across the different cell types. H) Marimekko charts depicting the proportions of each cell type or cluster separated by disease condition. I) Feature Plots depicting cluster defining genes. J) MALDI MSI of cortex and thalamus from Sandhoff disease patients and unaffected controls. Ion images show spatial distribution of gangliosides; color scale indicates intensity (arbitrary units). K) Volcano plot indicating the differentially regulated lipids between Sandhoff disease patients and unaffected controls measured by untargeted lipidomics (liquid chromatography mass spectrometry (LC-MS)). Two-tailed Welch’s t-test was used for statistical testing. Source data

To compare our mouse findings with the human disease, we performed histopathological analysis of CNS tissue of patients with Sandhoff disease and age- and sex-matched controls (Supplementary Table 1). IBA1 immunohistochemistry showed large foamy microglia with retracted processes in Sandhoff disease specimens only (Fig. 7a), particularly enriched in the cerebellum (Extended Data Fig. 9a). These microglia showed elevated levels of KIM1P, p22phox, lysozyme and LAMP2, consistent with lysosomal activation as observed in mice (Extended Data Fig. 9b). Haematoxylin and eosin stainings showed excessive intracellular inclusions and hypercellular Virchow–Robin spaces (Extended Data Fig. 9c,d). Inclusions were luxol fast blue (LFB)^+^ and mostly found in neurons and microglia (Fig. 7a). As in mice, microgliosis in individuals with Sandhoff disease coincided with axonal swelling and damage in the thalamic nuclei (Fig. 7a) with diminished axonal density in the thalamic white matter (Extended Data Fig. 9e). SMI31 immunohistochemistry showed phosphorylated neurofilament accumulation in neuronal somata, whereas SMI35 and SMI312 showed axonal swellings and impaired axonal transport (Extended Data Fig. 9f).

We then characterized microglia transcriptionally by snRNA-seq on 14,657 single nuclei from thalamic specimens. Cell-type annotation identified different structural CNS cell types, including astrocytes, oligodendrocytes, immune cells, oligodendrocyte precursor cells and neurons, among others (Fig. 7b and Extended Data Fig. 9g). Subsetting and re-clustering revealed four myeloid cell clusters (Fig. 7c), c0–2 as microglia (*CX3CR1*, *P2RY12*, *NAV3*) and c3 as CAMs (*F13A1*, *MRC1*, *CD163*, *LYVE1*) (Fig. 7d). Homeostatic microglia were concentrated in c0, whereas disease-linked cells appeared in c1 and c2 (Fig. 7c and Extended Data Fig. 9h). They strongly downregulated *CX3CR1*, *P2RY12*, *TMEM119* and *SALL1* and upregulated *GPNMB*, *MS4A7*, *MYO1E*, *SPP1* and *LPL*, mirroring the mouse response (Fig. 7d–f and Extended Data Fig. 9i). GO analysis further indicated enrichment of inflammatory signalling (c1) and lysosomal/autophagy pathways (c2) (Fig. 7d). Ultimately, untargeted lipidomics and spatial MALDI MSI of patient brain tissue showed GM2 species—such as GM2 34:1;O2 to 40:1;O2—in cortex and thalamus, closely matching the mouse GM2 profile (Fig. 7g and Extended Data Fig. 9j,k). In conclusion, brains of patients with Sandhoff disease recapitulate the transcriptional, histological and lipidomic signatures seen in *Hexb*-deficient mice, confirming a shared disease mechanism.

## Discussion

**Extended Data Fig. 10:**
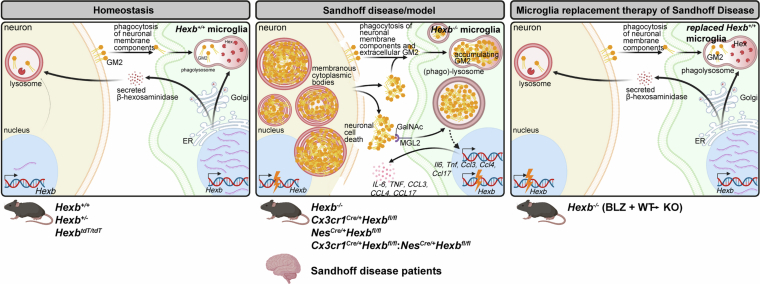
Graphical abstract of experimental findings. Left panel (Homeostasis): In the healthy brain, microglia expressing high levels of *Hexb* secrete functional Hex, which is taken up by neurons and delivered to their lysosomes. Middle panel (Sandhoff disease/model): In the absence of *Hexb*, both microglia and neurons accumulate GM2 ganglioside in their lysosomes. Neuronal cell death leads to extracellular GM2 release, which is sensed by microglia via the receptor MGL2, recognizing terminal GalNAc residues. This triggers a pro-inflammatory response, including the upregulation and secretion of TNF, IL-6, CCL3, CCL4, and CCL17. Right panel (Microglia replacement therapy): Transplantation of wild-type microglia into *Hexb*-deficient brains restores enzymatic function in neurons via microglial enzyme supply. The replaced microglia secrete functional Hex, thereby reducing neuronal and CNS-wide GM2 accumulation and promoting restoration of CNS homeostasis. Illustrations created using BioRender (https://biorender.com).

Our study describes a microglia–neuron connection that ensures normal brain homeostasis (Extended Data Fig. 10). By combining unbiased lipidomics, single-cell transcriptomics, spatial lipid imaging and new genetic models, we define a functional relationship in which the microglial lysosomal enzyme Hex regulates neuronal lipid balance. We began by monitoring the microglial core gene expression across models of neurodegeneration and demyelination. Although expression of *Tmem119*, *Siglech* and *Sall1* was reduced, *Cx3cr1* and *Hexb* remained stable. Using *Hexb*^*tdT*^ reporter mice^23^, we confirmed the microglial specificity and developmental regulation of *Hexb*. Although neurons expressed trace *Hexb* mRNA (approximately 200-fold lower), its functional significance was unclear^24^.

Global deletion of *Hexb* induced early-onset neurodegeneration, consistent with human Sandhoff disease^3,25^. However, the underlying molecular mechanisms and the cellular pathways involved remained incomplete. It has been described that a dysfunctional Hex leads to ganglioside storage within neurons and subsequent neuronal cell death with reactive microglia perpetuating a neurotoxic milieu^33^. Our histological and single-cell data show that microglial activation precedes astrocytic or neuronal changes, suggesting microglia are early responders. Along with our finding that full microglia replacement halts disease progression, this supports classifying *Hexb* deficiency as a new microgliopathy^36^.

The transcriptional profile of *Hexb*^*−/−*^ microglia partially resembled activated states seen in other neurodegeneration models, with upregulation of *Apoe*, *Ctsb*, *Csf1*, *Igf1*, *Lyst* and *Gpnmb* (refs. ^27,37^), alongside distinct markers such as *Apobec1*, *Flt1*, *Colec12*, *Adam33* or *Atp6v0d2*. *Gpnmb*, the most upregulated gene, encodes a transmembrane glycoprotein induced in lipid-laden macrophages^38,39^ and linked to anti-inflammatory^40^ and tissue repair roles^41^. Importantly, phagocytosis and autophagy pathways, both commonly disrupted in various lysosomal storage disorders^42^, were strongly altered in *Hexb*^*−/−*^ microglia, underscoring the enzyme’s key role in lysosomal function.

Another strongly upregulated gene in *Hexb*-deficient microglia was *Ms4a7*, previously considered a marker of peripheral myeloid origin^43^. However, our data suggest that *Ms4a7* reflects activation state rather than ontogeny, highlighting the limitations of using single-gene markers in distinguishing resident from peripherally derived myeloid populations in the CNS.

To understand how microglial enzyme loss causes neurodegeneration, we dissected the mechanism of Hex transfer. Microglia secrete the enzyme by means of the Golgi pathway into the extracellular space, where it is taken up by neurons to degrade GM2 gangliosides. Lipidomics confirmed GM2 as the most excessively accumulated lipid. Notably, GM2 species with longer ceramide backbones increased with disease progression, reflecting postnatal developmental shifts in ganglioside composition^44^. Still, it is unknown how this shift occurs and what functional significance it holds for homeostasis and Sandhoff disease.

GM2 accumulation in both mouse and human *Hexb*-deficient brains correlated with strong microgliosis, suggesting a causal link between these two events. Indeed, GM2—but not GM1 or GM3—induced proinflammatory cytokines in microglia, indicating a specific immune response. Interestingly, although GM1 gangliosidosis is also associated with pronounced microglia activation and cytokine release^45^, this effect does not seem to stem from direct GM1 recognition. In fact, GM1 has been shown to exert anti-inflammatory effects on microglia^46^. We identified MGL2 as the key receptor mediating GM2’s effects. Expressed in dendritic cells, macrophages and microglia, MGL2 binds terminal GalNAc residues^30^. In general, its signalling is context- and ligand-dependent^47^, with evidence for both pro- and anti-inflammatory responses^48,49^.

To pinpoint disease-driving cell types in vivo, we generated conditional knockouts targeting *Hexb* in specific compartments. Contrary to our expectations, deleting *Hexb* in microglia alone did not induce disease. Only combined depletion in neurons and microglia recapitulated the full phenotype, indicating functional redundancy. Similar compensation has been observed in other contexts—for example, *Grn* deletion in microglia alone does not trigger CNS pathology^50^. Overall, although the absence of a functional *Hexb* gene in microglia alone does not cause neurodegeneration, the presence of a functional *Hexb* gene only in microglia is sufficient to prevent neurodegeneration. However, microglia probably contribute to pathology by amplifying inflammation. We describe IL-6, TNF, CCL3, CCL4 and CCL17 release from microglia upon GM2 exposure and identified upregulated phagocytic pathways in microglia potentially promoting disease progression. A similar mechanism has been described in Gaucher disease, in which lipid-accumulating microglia phagocytose live neurons^51^, and, more broadly, lipid metabolic dysfunction has been tied to neuroinflammation^52,53^.

Beyond GM2, we identified several lipids accumulating in the brains of *Hexb* mutant mice, including GA2, GalNAc–GM1 36:1–O2 (also known as asialo-GM2) and BMP 44:12. Our spatiotemporal lipid map revealed pathological hotspots, but the direct link between ganglioside buildup and neuron loss remains unclear. A recent study implicated neuron-intrinsic cGAS–STING signalling in *Hexb*-related neurodegeneration^54^, but the specific vulnerability of neuronal subtypes to GM2 stress warrants further investigation.

At present, no curative therapies exist for Sandhoff disease. Besides symptomatic treatment, lysosomal storage disorder therapies available and approved at present—enzyme replacement therapy, substrate reduction therapy or chaperone therapy^55^—are often prohibitively expensive and primarily serve to slow disease progression rather than halt it. Notably, enzyme replacement therapy fails to effectively address the CNS involvement seen in many lysosomal storage disorders, as the blood–brain barrier prevents administered enzymes from reaching the brain parenchyma. Gene therapy, however, holds promise: AAV-based delivery of *Hexb* complementary DNA has shown success in mice^56^ and these findings have been translated to human GM2 gangliosidoses^57^, but achieving full CNS coverage remains challenging. Bone marrow transplantation offers an alternative but has shown limited benefit in GM2 gangliosidosis^58,59^, probably owing to insufficient engraftment of enzyme-competent myeloid cells. Previous studies, including ours, have shown that CNS preconditioning (for example, irradiation) is required for myeloid cell engraftment^60^, which can be significantly augmented in several inflammatory and neurodegenerative models^61,62^. Still, microglia replacement rarely exceeds 30%. However, higher microglia and CAM turnover with circulating blood cells has been described recently in aged patients without CNS diseases^63^. In a mouse model of Sandhoff disease, a study improved the engraftment of *Hexb*^+^ MLCs by first depleting resident microglia pharmacologically, then transplanting cultured mouse microglia into the open niche, which prevented disease symptoms and attenuated neurodegeneration^64^. Building on these findings, we used clinically applicable bone marrow transplantation combined with microglia depletion to efficiently replace microglia and demonstrate that donor cells restored enzymatic activity, reduced GM2 and shifted microglial transcription towards homeostasis. Early intervention further improved outcomes. This treatment approach might also be a new potential therapeutic option for microglia-mediated human gangliosidoses. Similarly, our detailed dissection of the microglia–neuron crosstalk that regulates GM2 turnover could inform strategies for cell replacement therapy in Sandhoff disease and other gangliosidoses. In summary, our findings expand the understanding of microglial functions in the healthy CNS to include the homeostatic regulation of neuronal membrane components.

## Methods

### Mice

*Hexb*^*−/−*^ mice (B6.129S-Hexbtm1Rlp/J) were purchased from The Jackson Laboratory (002914) and backcrossed for five generations with C57BL/6J mice. *Hexb*^*fl/fl*^ mice (*B6.Hexb*^*tm1c(EUCOMM)Hmgu*^*/H*) were generated by crossing the *B6.Hexb*^*tm1a(EUCOMM)Hmgu*^*/H* line from the MRC Harwell Institute with the Flp-FRT line (129S4/SvJaeSor-Gt(ROSA)26Sor^tm1(FLP1)Dym^/J). *Hexb*^*fl/fl*^ mice were further crossed with *Cx3cr1*^*cre*^ (B6J.B6N(Cg)-*Cx3cr1*^*tm1.1(cre)Jung*^/J) or *Nes*^*cre*^ (B6.Cg-Tg(Nes-cre)1Kln/J) mouse lines. *Cx3cr1*^*GFP*^ (B6.129P2(Cg)-*Cx3cr1*^*tm1Litt*^/J) mice served as bone marrow donors. In addition, *Cx3cr1*^*GFP*^, Thy1^*GFP*^ (B6.Tg(Thy1-EGFP)MJrs/J) and Hexb^*tdT*^ (B6N.Hexb^em1Mp^) mice were used for imaging analysis. Littermates were used as controls in all experiments. For all experiments, mice were randomly assigned to experimental groups on the basis of their genotype. No statistical methods were used to predetermine sample sizes. For the analysis of different neurodegenerative and demyelinating disorders, the following mice (and brain regions) were used. SOD1 mice (B6.Cg-Tg(SOD1*G93A)1Gur/J; spinal cord) and R6/2 mice (B6CBA-Tg(HDexon1)62Gpb/3J; striatum) were purchased from The Jackson Laboratory (004435, 006494). In addition, 5xFAD mice (B6.Cg-Tg(APPSwFlLon,PSEN1*M146L*L286V)6799Vas/Mmjax; hippocampus) and APP23 mice (B6.Cg-Tg(Thy1-APP)3Somm/J; cortex) were used. Cuprizone-induced demyelination was achieved by feeding mice for 5 weeks with 0.25% (wt/wt) cuprizone (C9012, Sigma-Aldrich) in the ground breeder chow (corpus callosum). Wild-type female mice on C57BL/6N background were used as controls.

Mice were housed under a 12-h light/12-h dark cycle and at temperatures of 18–23 °C with 40–60% humidity, with food and water provided ad libitum. Diseased mice received wet food placed on the cage ground. All animal experiments were approved by the local administration (Regierungspräsidium Freiburg, approval numbers G-17/34, G-21/020 and G-22/035) and were performed in accordance with the respective national, federal and institutional regulations. Upon weight loss (more than 10% loss) or occurrence of an impaired righting reflex (more than 5 s), diseased mice were euthanized and the age was recorded.

### Behavioural testing

#### Rotarod

Motor coordination was assessed using the rotarod assay. The assay was conducted using a Rota-Rod (Model 47650, Ugo Basile) with accelerating speed (accelerated from 3 to 40 rpm over 300 s). The mice were trained on the accelerating rotarod 1 day before the first recorded testing. The latency to fall off the rotarod was recorded three times a week until the end of the observation period. Only one trial was conducted per day. A full passive rotation or falling off the rotarod was considered a failure and recorded as ‘latency to fall’.

#### Grip strength test

Muscular strength was assessed with the grip strength test using a Grip-Strength-Meter (Mains). Mice were grasped at their tail and placed on a slightly oblique grid with all four limbs. Next, the tail was gently and continuously pulled backwards. The maximum force was automatically recorded when the mouse lost its grip. Each mouse was tested three times; the best trial was recorded.

### Human specimens and ethics

Human tissues were obtained from the National Institutes of Health (NIH) Neurobiobank at the University of Maryland, Baltimore, MD. Samples were shipped on dry ice and stored at −80 °C until used. Detailed information about the human specimens is provided in Supplementary Table 1. The examination of adult autopsy tissues was carried out with supervision from the Research Ethics Committee at the University Freiburg Medical Center, following protocol numbers 10008/09 and 472/15, as well as oversight from local committees affiliated with the NIH biobanks. Written, informed consent was obtained from the patients or their legal guardians before the procedures.

### Nuclei isolation from frozen tissues

Nuclei isolation was performed as previously described^63^. In brief, a small tissue fragment was homogenized and incubated in 500 µl of ice-cold nuclei EZ lysis buffer (NUC101-1KT, Sigma-Aldrich) for 5 min. After filtration through a 70-µm filter (B60160056, Miltenyi) and centrifugation at 500*g* for 6 min at 4 °C, the supernatant was removed. Subsequently, 1 ml of ice-cold EZ lysis buffer was added, followed by incubation on ice for 5 min. After centrifugation, the supernatant was discarded, and the pellet was incubated for 5 min with 0.5 ml of nuclei buffer (1 × DPBS (D8537, Sigma-Aldrich), 1% BSA (130-091-376, Miltenyi), 0.2 U μl^−1^ RNase inhibitor (M0314L, New England Biolabs)). After gentle pipetting and centrifugation, the washing step was repeated with 1 ml of nuclei buffer. Following another centrifugation step, the supernatant was removed, and the nuclei were incubated for 10 min with a staining mix containing DAPI (10 µg ml^−1^), and for human samples Anti-Olig2 Alexa-488 (1:100) (ab225099, abcam), and anti-NeuN Alexa-647 (1:100) (ab190565, abcam) antibodies in a total volume of 200 µl of nuclei buffer. After further centrifugation, the supernatant was discarded, and the pellet was re-suspended in 300 µl of nuclei buffer, filtered through a 40-μm cell strainer (14-100-150, Thermo Fisher) and subjected to FACS nuclei sorting. Nuclei were sorted on a MoFlo Astrios (Beckman Coulter) or BD FACSAria III machine (BD Bioscience).

### 10x Genomics droplet-based single-nucleus library preparation

Per reaction, up to 40.000 DAPI^+^ mouse nuclei or DAPI^+^Olig2^*−*^NeuN^*−*^ human nuclei were sorted into Eppendorf tubes. The gating strategies for FACS, related to the datasets in Fig. 3a–c and Fig. 7b, are shown in Supplementary Fig. 4a. Single nuclei were packaged into droplets and lysed, followed by barcoding through mRNA reverse transcription using the Chromium controller with the Chromium Next GEM Single Cell 3′ Kit v.3.1 (10x Genomics). cDNA amplification and library preparation were conducted following the manufacturer’s instructions. Libraries were sequenced on a NextSeq1000 (Illumina) appropriate to reach 20,000 reads per cell. The resulting fastq files were further processed using the Cell Ranger v.7.1.0 pipeline (10x Genomics) for demultiplexing, read alignment either to the mouse (GRCm38, mm10) or human genome (GRCh38p13, Gencode v.35, hg38) and gene count determination.

### Doublet detection, quality control and analysis of the single-nucleus transcriptomic data

Mouse and human transcriptomic data were analysed in RStudio (Build 421), with R programming language v.4.3.2 (ref. ^65^). Filtered counts matrices were loaded with Seurat v.5.0.3 (ref. ^66^). Doublets were excluded using the scDblFinder package v.1.16.0 (ref. ^67^). Therefore, the Seurat object was transformed into a SingleCellExperiment object using the as.SingleCellExperiment function, the scDblFinder function was run and the original Seurat object was filtered for cells classified as ‘singlet’. The data from different experiments were merged into one Seurat object (merge). For mouse samples, a three-step procedure for strict quality control was applied. First, only nuclei with less than 0.1% mitochondrial transcripts, less than 0.1% haemoglobin transcripts and between 300 and 4,500 genes expressed were retained. Second, after an initial normalization and integration (see below), clusters expressing ‘debris marker’ defined by the FindMarkers function were excluded. Debris markers were previously identified by loading raw counts matrices and, after normalization and integration, identifying marker genes (FindMarkers) of cluster 0 containing empty partitions and low-quality cells (*Tuba1a*, *Hspa8*, *Atp6v0c*, *Tubb2a*, *Ubb*). Third, nuclei were manually inspected using the FeatureScatter and CellSelector functions to visualize known cell-type-specific marker genes. Nuclei expressing more than one cell-type-specific marker were filtered^21,22,68–70^. Because the mouse dataset contained nuclei of all CNS cell types, the amounts of expressed genes differ between cell types and clusters. Thus, a further round of nuclei exclusion was performed on the basis of expressed genes (nFeature_RNA) for annotated cell types as follows: astrocytes (500–2,000 expressed genes), immune cells (300–1,800 expressed genes), vascular leptomeningeal cells (800–2,000 expressed genes), ependymal cells (300–2,000 expressed genes), oligodendrocytes (1,800–3,800 expressed genes), oligodendrocyte precursor cells (600–2,800 expressed genes), committed oligodendrocyte precursors (600–2,800 expressed genes), neurons (1,300–3,800 expressed genes). For human samples, nuclei with at least 300 and fewer than 4,500 detected genes and below 2% mitochondrial transcripts were included. In addition, myeloid cells with more than 2,500 detected genes were discarded. Visualization was achieved using the DimPlot function. After quality control, the data were normalized (NormalizeData) and scaled on the 2,000 most variable features (FindVariableFeatures, ScalaData). Linear dimensional reduction was performed using the RunPCA function. Next, the different experiments within the Seurat object were integrated using the Harmony R package v.1.2.0 (RunHarmony)^71^. Last, UMAP embedding and shared nearest-neighbours graph construction were performed on the top ten principal components (top seven principal components for human data) (RunUMAP, FindNeighbors), and cell clusters were identified with a resolution set to 1.2 (0.5 for human data) (FindClusters).

### Cell annotations, differential gene expression analysis and GO enrichment analysis

For every cluster, DEGs were calculated using the FindAllMarkers function with logfc.threshold and min.pct arguments set to 0.25. DEGs were used for cluster annotation on the basis of published cell-type-specific marker genes (Supplementary Tables 2, 17 (mouse) and 11 (human)). For further analysis of the mouse data, immune cells and subsequently microglia were abstracted to generate a new Seurat object using the subset function. Re-normalization, re-scaling, re-integration and re-clustering were performed as described above. Again, cluster markers were calculated using the FindAllMarkers function with logfc.threshold and min.pct arguments set to 0.25 (Supplementary Tables 3, 4, 18 and 19). For further analysis of the human data, myeloid cells were isolated into a new Seurat object (subset). Re-normalization, re-scaling, re-integration and re-clustering were performed with slight modifications: data were scaled on the 5,000 most variable features and the variables ‘percent.mt’ and ‘percent.rp’ were regressed out. The different participants were integrated using Harmony and UMAP embedding and shared nearest-neighbours graph constructions were performed on the top ten principal components. Then, cluster markers were calculated using the FindAllMarkers function with default settings using the MAST algorithm^72^ (Supplementary Table 12). The significant (adjusted *P* < 0.05) cluster marker genes were subjected to GO enrichment analysis performed with the clusterProfiler v.4.10.0 package^73^. Maker genes were transformed into entrezIDs and the enrichGO function was run on them to identify enriched biological processes. Microglia clusters were re-ordered from cluster size to altered biological function. For direct comparison of clusters, FindMakers function was run with default settings using the MAST algorithm^72^ (Supplementary Tables 5–8, 20 (mouse) and 13–15 (human)). Data were visualized using DimPlot, DotPlot, FeaturePlot and DoHeatmap Seurat functions. In addition, the EnhancedVolcano package (v.1.20.0) was used to generate volcano plots^74^.

### Cross-species analysis

For the comparison of mouse and human disease microglia, DEGs for mouse and human disease microglia were calculated independently by comparing disease-associated clusters with homeostatic clusters (FindMakers). DEGs were filtered for statistical significance (adjusted *P* < 0.05). Mouse gene names were converted to human orthologous gene names using the R package BiomaRt (v.2.58.2)^75^. Only genes with a corresponding orthologous name were retained (Supplementary Table 16). Using the ggplot2 package (v.3.5.0)^76^, average log_2_(FC) values of human and mouse genes were plotted.

### Bulk RNA sequencing

Microglia were FACS sorted from whole brains (see gating strategies used for FACS shown in Supplementary Fig. 4b) into a collection tube and then total RNA was extracted using PicoPure RNA Isolation Kit (KIT0204, Life Technologies), according to the manufacturer’s protocol. The SMARTer Ultra Low Input RNA Kit for Sequencing v.4 (Clontech) was used to generate first-strand cDNA from approximately 1 ng of total RNA. Double-stranded cDNA was amplified by long-distance PCR (ten cycles) and purified by means of magnetic bead clean-up. Library preparation was carried out as described in the Illumina Nextera XT Sample Preparation Guide (Illumina). Thereby, 150 pg of input cDNA was tagmented (tagged and fragmented) by the Nextera XT transposome. The products were purified and amplified through a limited-cycle PCR programme to generate multiplexed sequencing libraries. The libraries were quantified using the KAPA Library Quantification Kit–Illumina/ABI Prism User Guide (Roche Sequencing Solutions). Equimolar amounts of each library were sequenced on an Illumina NextSeq 2000 instrument controlled by the NextSeq 2000 Control Software v.1.4.1.39716, using two 50-cycle P3 Flow Cells with the dual index, single-read run parameters. Image analysis and base calling were done by Real Time Analysis software v.3.9.25. The resulting .cbcl files were converted into .fastq files with bcl2fastq v.2.20 software. Fastq files were quality controlled using FastQC v.0.73 and trimmed with Trim Galore! v.0.6.7. Reads were mapped to the GRCm39 mouse genome using RNA STAR aligner v.2.7.8. Read counts were obtained using featureCounts v.2.0.1. We performed differential gene expression analysis using the limma-trend pipeline v.3.50.1 (Supplementary Table 22). GO enrichment analysis of DEGs was done using goseq v.1.44.0. The mentioned processes were run on the galaxy platform^77^. PCA analysis was conducted with the ggfortify R package (v.0.4.17). Heat maps were generated using the R package pheatmap (v.1.0.12). Venn diagrams were generated using previously published tools (https://bioinformatics.psb.ugent.be/webtools/Venn/). Volcano plots were calculated with the R package EnhancedVolcano.

### Flow cytometry

For blood cell analysis, one drop of blood was collected from the facial vein into FACS buffer (PBS containing 2% BSA (8076.3, Roth) and 10 mM EDTA (15575020, Invitrogen)) to prevent clotting. Blood cells were centrifuged at 300*g* for 5 min at 4 °C. The blood cell pellet was re-suspended in RBC lysis buffer (00-4333-57, Thermo Fisher) and incubated for 2 min at room temperature. Ice-cold FACS buffer was added and cells were centrifuged again before staining. For microglia analysis, mice were anaesthetized and transcardially perfused with ice-cold PBS. Brains were roughly minced and homogenized with a Potter tissue grinder in HBSS (14170-138, Gibco) containing 15 mM HEPES (15630080, Gibco) buffer and 0.54% glucose (G8769, Sigma). Whole-brain homogenate was separated by 37% Percoll (P1644, Sigma) gradient centrifugation at 800*g* for 30 min at 4 °C (no brake). The pellet containing CNS macrophages at the bottom of the tube was then collected and washed once with FACS buffer before staining. Fc receptors were blocked with Fc Block (2.4G2, BD Biosciences) for 15 min at 4 °C before incubation with the primary antibodies. Cells were stained with antibodies directed against CD11b (1:300, M1/70, BioLegend), CD45 (1:200, 30-F11, Invitrogen), Ly6C (1:300, AL-21, BD Biosciences), Ly6G (1:300, 1A8, BD Biosciences), CD115 (1:200, AFS98, Invitrogen), CD64 (1:200, X54-5/7.1, BioLegend), CD11c (1:300, N418, Invitrogen), CD3e (1:300, eBio500A2, Invitrogen), CD19 (1:300, eBio1D3, Invitrogen), B220 (1:300, RA3-6B2, BioLegend) and CD206 (1:200, C068C2, BioLegend) for 45 min at 4 °C. After washing, cells were sorted using a MoFlo Astrios (Beckman Coulter) or analysed using a BD LSRFortessa (Becton Dickinson). Viable cells were gated by staining with DAPI. Data were acquired with FACSDiva or Summit software (Becton Dickinson). We performed post-acquisition analysis using FlowJo software, v.10.5.3. Gating strategies for blood cells and microglia are shown in Supplementary Fig. 4c,d.

### Chromogenic immunohistochemistry and cell quantifications

Mice were anaesthetized and transcardially perfused with ice-cold PBS. Brains were fixed in 4% formalin and embedded in paraffin. Then, 3-μm paraffin sections were initially deparaffinized at 80 °C for 1 h, then deparaffinized in xylene and incubated in EnVision FLEX Target Retrieval Solution pH 6 cooking buffer (S1699, DAKO) for 40 min at 95 °C. Endogenous tissue peroxidase was blocked in 3% hydrogen peroxidase for 10 min. Samples were blocked with PBS containing 5% BSA (8076.3, Roth) and permeabilized with 1% Triton X-100 (T8787, Sigma) for 1 h. Primary antibodies were added overnight at a dilution of 1:1,000 for IBA1 (ab178846, Abcam), 1:200 for Mac-3 (553322, BD Pharmingen), 1:10,000 for GFAP (Z0344, DAKO), 1:3,000 for APP (MAB348, Merck), 1:500 for P2RY12 (AS-55043A, Anaspec), 1:500 for TMEM119 (400 002, Synaptic Systems), 1:1,000 for NeuN (ab104224 or ab177487, abcam) and 1:500 for HEXB (LS-B16803, LSBio) at 4 °C. After three washes with PBS, biotinylated secondary antibodies (Southern Biotech) were added as follows: goat-anti-mouse 1:200 (1031-08), goat-anti-rabbit 1:300 (4050-08) and goat-anti-rat 1:200 (3050-08) for 45 min at room temperature. Three more washing steps were performed before incubating the sections with streptavidin peroxidase (PK-6100, Vector Laboratories) for 45 min at room temperature. After three more washes with PBS, slides were incubated with diaminobenzidine (DAB) solution: 1 drop of EnVision Flex DAB Chromogen (DM827, DAKO) per 1 ml of EnVision Flex Substrate Buffer (DM823, DAKO). For double-immunolabelling, the process was repeated using streptavidin-AP and Permanent Red as chromogen. Finally, the slides were counterstained with Gill’s haematoxylin solution (11769, Morphisto). Coverslips were mounted with xylene-based Vitro-Clud mounting medium (04-0001, Langenbrinck) or Kaisers Glycerin-Gelatine (6474.1, Roth), respectively. For quantification, images were taken using a BZ-X810 microscope with BZ-X8000 Analyzer software (Keyence). To assess density of cells, IBA1^+^, Mac-3^+^ and GFAP^+^ cells or APP^+^ deposits were quantified manually as previously described^78^. To assess HEXB^+^ neurons, only large cells with a visible cytoplasm and prominent nucleolus were analysed. At least three sections of a minimum of three mice were used for each analysis. Representative images were acquired with a Leica DFC450 Digital Microscope Camera. Post-acquisition editing was done with Adobe Photoshop CS4.

Brain tissue obtained at autopsy was fixed in buffered formalin and embedded in paraffin. Next, 3-μm-thick paraffin sections were treated as previously described^79^. Briefly, after deparaffinization in xylene, sections were transferred to 99.5% ethanol and rehydrated to distilled water using decreasing ethanol series. Staining for macrophages/activated microglia (clone KiM1P, 1:50), IBA1 (1:1,000, ab178846, Abcam), APP (1:2,000, MAB348, Sigma), p22phox (1:100, sc-20781, Santa Cruz) and phosphorylated neurofilament (1:5,000, SMI31, Sternberger Monoclonals) required antigen retrieval with citrate buffer (antigen retrieval solution, Dako), whereas for lysozyme (1:200, 18-0038, Zymed) protease pretreatment and for LAMP2 (1:500, SA46-01,Thermo Fisher) TE-buffer was used, each for 50 min in a steaming device (Braun). Neurofilament (SMI35, hypophysphorylated, and SMI312, highly phosphorylated, both 1:1,000, Sternberger Monoclonals) required no antigen retrieval. Sections were blocked in 10% FCS for 10 min and 3% H_2_O_2_ before incubation with primary antibodies for 90 min at room temperature. After washing in PBS, sections were incubated with biotinylated secondary antibodies, then incubated with avidin-coupled horseradish peroxidase and finally developed in DAB. For KiM1P immunohistochemistry, DAB Envision Kit (Dako) was used. Nuclei were visualized with haematoxylin. Light microscopy sections were photographed using an Olympus BX51 microscope and cellSense software (Olympus).

### Fluorescent immunohistochemistry, cell quantifications, analysis and IMARIS-based 3D reconstruction

After transcardial perfusion with ice-cold PBS, brains were fixed for 5 h in 4% formalin, dehydrated in 30% sucrose and embedded in Tissue-Tek O.C.T. compound (Sakura Finetek). Then, 14-µm (for quantification), 30-μm (for representative images) or 50-μm cryosections (for 3D reconstruction) were obtained and blocked with PBS containing 5% BSA and permeabilized with 0.5% Triton X-100 in blocking solution (no Triton X-100 was used for GM2 stainings). Primary antibodies were added overnight at a dilution of 1:1,000 for IBA1 (ab178846, Abcam; 234 308, Synaptic Systems), 1:500 for CD206 (MCA2235, Bio-Rad), 1:200 for collagen IV (AB769, Millipore), 1:500 for SOX9 (AF3075, R&D), 1:500 for NeuN (ab104224, abcam), 1:200 for OLIG2 (ab109186, abcam), 1:100 for GM2 (A2575, TCI), 1:500 for TMEM119 (400 002, Synaptic Systems), 1:500 for P2RY12 (AS-55043A, Anaspec), 1:100 for CD68 (MCA1957, Bio-Rad) and 1:500 for LAMP1 (PA1-654A, Invitrogen) at 4 °C. Secondary antibodies were purchased from Thermo Fisher and added as follows: Alexa Fluor 405 1:500, Alexa Fluor 488 1:500, Alexa Fluor 568 1:500 and Alexa Fluor 647 1:500 for 90 min at 4 °C. For nuclear counterstaining, DAPI was added for 30 min at room temperature. Coverslips were mounted with Mowiol (0713.2, Roth). For quantification, images were taken using the conventional fluorescence microscope BZ-X810 (Keyence). To assess density of cells, numbers of IBA1^+^CD206^−^ (microglia) or CD206^+^ cells (perivascular macrophages and leptomeningeal macrophages) were quantified. Microglia and perivascular macrophages were normalized to the area of the region of interest and expressed as cells per millimetre squared. Leptomeningeal macrophages were normalized to the length of the leptomeninges indicated by collagen IV or laminin immunofluorescence and finally expressed as cells per mm. To assess labelling for tdTomato, IBA1^+^P2RY12^+^ microglia, CD206^+^ perivascular macrophages and leptomeningeal macrophages, SOX9^+^ astrocytes, NeuN^+^ neurons and OLIG2^+^ oligodendrocytes were counted and analysed. At least three sections of a minimum of three mice were used for each analysis. Representative confocal images are taken with the TCS SP8 X (Leica) using a ×20 or ×63 objective, respectively. Post-acquisition editing was done with LAS X software (Leica) and Adobe Photoshop CS4. For quantification of the lysosomal ganglioside burden, lysosomal compartments were segmented on the basis of LAMP1 fluorescence. Within these LAMP1^+^ regions, the mean fluorescence intensity of the GM2 signal was measured. For each cell (defined by IBA1 or NeuN signal), the GM2 signal across all lysosomal regions was summed to obtain the total lysosomal GM2 fluorescence per cell. This total lysosomal GM2 signal was then normalized to the corresponding cell area to allow comparison between cell types. We performed image analysis using ImageJ (v.1.54g). For 3D reconstruction of microglia and lysosomes, sections were co-stained with IBA1 and CD68. IBA1^+^ parenchymal cells were selected and imaged with the TCS SP8 X (Leica) using a ×63 objective with z-stacks of 0.3 µm. 3D reconstructed cell images and statistical read outs were obtained using Imaris software v.9.6.0 (Bitplane).

### Lipid measurement by liquid chromatography–mass spectrometry

For the extraction of lipid for liquid chromatography–mass spectrometry, frozen mouse brains were thawed on ice for 1 min, and 10 mg of cortical brain tissue was mechanically homogenized in 1 ml of 20% methanol (Carl Roth, 8399.1). Cultured NPCs were washed three times with PBS, collected and mechanically homogenized in 0.1 ml of 20% methanol. Next, 500 µl of the homogenate was diluted in 750 µl of ddH_2_O plus 2.5 ml of 1-butanol, vortexed for 1 min and centrifuged for 1 min at 20,000*g* to separate phases. The top layer (butanol) was transferred to a 4-ml glass tube. Then, 1 ml of water-saturated 1-butanol was added to the remaining aqueous phase, vortexed for 1 min and centrifuged for 1 min at 20,000*g* to separate phases. The top layer was combined with the butanol phase obtained in the first extraction. The butanol phase was dried in a speedvac. Just before measurement, the pellets were re-suspended in 50 µl of a 2:1:1 mixture of 2-propanol, acetonitrile and ddH_2_O. Non-targeted measurement of lipids by liquid chromatography–mass spectrometry was carried out as described previously^80^ using an Agilent 1290 Infinity II UHPLC in line with a Bruker Impact II QTOF-MS operating in negative ion mode. Briefly, the scan range was from 50 to 1,600 Da. Mass calibration was performed at the beginning of each run. Liquid chromatography separation was on a Zorbax Eclipse Plus C18 column (100 × 2 mm^2^, 1.8-µm particles) using a solvent gradient of 70% buffer A (10 mM ammonium formiate in 60:40 acetonitrile:water) to 97% buffer B (10 mM ammonium formiate in 90:10 2-propanol:acetonitrile). Flow rate was 400 µl min^−1^, autosampler temperature was 5 °C and injection volume was 2 µl. Data processing including feature detection, feature deconvolution and annotation of lipids and was performed using MetaboScape (v.2023b). Differentially regulated lipids are listed in Supplementary Tables 9, 10 and 21.

### MALDI MSI

Fresh-frozen mouse and human brain tissues were sectioned at 10-μm and 20-μm thickness, respectively, with a Leica CM1950 cryostat (Leica Biosystems) at −18 °C chamber and specimen head temperature. Sections were thaw-mounted onto ITO slides (Bruker Daltonics) and stored at −80 °C. For further use, slides were brought to room temperature and dried for 15 min in a vacuum desiccator. Optical images were acquired using a Tissue Scout slide scanner (Bruker Daltonics). For matrix spray-coating, 2,5-dihydroxyacteophenone (DHAP) was suspended in 7:3 (v/v) acetonitrile:H_2_O at 10 mg ml^−1^. The suspension was vortexed and sonicated until solid DHAP was fully dissolved. Then, 0.1% (v/v) trifluoroacetic acid was added and the mixture was vortexed. The matrix was deposited with an M5 TM-Sprayer (HTX Technologies). Temperatures of the spray nozzle and tray were 75 °C and 35 °C, respectively. The spraying parameters were as follows: Spray Nozzle Velocity: 1,200 mm min^−1^; Flow Rate: 0.1 ml min^−1^; No. of Passes: 10; Track Spacing: 2 mm; Pattern: HH; Pressure: 10 psi; Gas Low Rate: 2 l min^−1^; Nozzle Height: 40 mm; Drying Time: 0 s. Before MSI data acquisition, external mass calibration was achieved using red phosphorus (RedP) clusters P_*n*_ (*n* = 13–61 in intervals of 4) and an enhanced-quadratic calibration model. MALDI MSI was carried out on a timsTOF fleX system (Bruker Daltonics) equipped with a smartbeam 3D 10-kHz laser, TimsControl 5.0(4.1) and flexImaging v.7.4(7.2) software (Bruker Daltonics). Data were acquired in negative ion mode (*m*/*z* range of 300–2,500) with 200 laser shots per pixel, 10-kHz laser frequency and lateral step size 40 µm. The Ion Transfer parameters were as follows: MALDI Plate Offset 50 V, Deflection 1 Delta −70 V, Funnel 1 RF 400 Vpp, isCID Energy −0.0 V, Funnel 2 RF 400 Vpp and Multipole RF 380 Vpp. Collision Cell parameters: Collision Energy 10 eV and Collision RF 2,000 Vpp. Quadrupole parameters: Ion Energy 5 eV and Low Mass *m*/*z* 320. Focus Pre TOF parameters: Transfer Time 105 µs and Pre Pulse Storage 12 µs. For the human brain tissue sections, the internal standard SM4 35:1;O2 (C41H79NO11S, [M-H]-; *m*/*z* 792.530107) was used for internal lock-mass calibration.

### MSI data evaluation and visualization

MSI data (centroided) were imported into SCiLS Lab 2024a Pro (Bruker Daltonics) and root mean square-normalized. Data were then exported as imzML files and uploaded to www.metaspace2020.eu for annotation of putative metabolites using the following settings: *m*/*z* tolerance 5 ppm, Analysis Version v.2.20230517 (META-SPACE ML https://www.biorxiv.org/content/10.1101/2023.05.29.542736v1), databases SwissLipids-2018-02-02 and LipidMaps-2017-12-12. For ganglioside annotation, a further in-house library based on theoretical masses was used. Average peak intensities were exported from SCiLS Lab, *Z*-score transformed and visualized as a heat map using R. Ion images were exported from SCiLS Lab in viridis colour scale, within a mass window of ±12 ppm.

### Western blot

Cells were lysed in lysis buffer (50 mM HEPES pH 7.4, 40 mM NaCl, 2 mM EDTA, 1.5 mM NaVO_4_, 30 mM NaF, 10 mM sodium pyrophosphate, 10 mM sodium beta glycerophosphate and protease inhibitors (Sigma-Aldrich, A32965)) supplemented with 1% Triton X-100. Cell culture supernatant was diluted at 1:1 with lysis buffer. Protein lysates were resolved by 4–12% SDS–PAGE at 80–120 V. Resolved proteins were transferred for 90 min at 100 V to methanol-pretreated PVDF membranes to be further analysed by immunoblotting. Membranes were blocked with 5% non-fat dry milk prepared in TBST (Tris-buffered saline with 0.1% Tween 20) for 1 h at room temperature, then incubated overnight with the following primary antibodies diluted in TBST supplemented with 1% milk at 4 °C on a rotor: anti-TSG101 (ab125011, abcam, 1:2,000), anti-GAPDH (2118, Cell Signaling, 1:1,000), anti-His (66005-1-Ig, Proteintech, 1:5,000). Following incubation, membranes were washed with TBST three times for 5 min each, before incubating with the appropriate secondary antibodies diluted 1:2,000 in 1% BSA containing TBST for 1 h at room temperature. Membranes were then washed three times with TBST before being visualized using SuperSignal West Pico PLUS Chemiluminescent Substrate (34579, Life Technologies) (Supplementary Fig. 5).

### Hex activity assay

For the Hex assay, an established protocol was followed^81,82^. In brief, whole brains were homogenized with a Potter tissue grinder in KPBS (136 mM KCl, 10 mM KH_2_PO_4_, pH 7.25). The homogenate was centrifuged for 2 min, 1,000*g*, 4 °C. The brain homogenate, FACS-sorted microglia or bead-purified neurons were lysed with KPBS containing 1% Triton X-100 for 10 min on ice. In a total volume of 40 ml per reaction, the cell lysates were incubated at 37 °C in a 10 mM sodium citrate buffer (pH 4.2) containing 2 mM 4-methylumbelliferyl-2-acetamido-2-deoxy-b-d-glucopyranoside (69585, Sigma). The reaction was stopped by adding 5 volumes of a 0.2 M glycine/0.2 M Na_2_CO_3_ solution. The amount of liberated 4-methylumbelliferone was determined fluorometrically at an emission wavelength of 440 nm after excitation at 365 nm.

### Primary microglia cell culture

Primary microglia were cultured as previously described^83^. In brief, P0–2 newborn mouse pups were decapitated, and the brain was removed and placed in ice-cold dissection media (HBSS (24020117, Gibco), 10 mM HEPES (15630080, Gibco), 35 mM glucose, 100 U ml^−1^ penicillin–streptomycin (15140122, Gibco)). Meninges were removed and cortices were microdissected and placed in 30 ml of fresh dissection media. Then, 1.5 ml of trypsin (15090046, Gibco) was added and incubated for 15 min at 37 °C. After incubation, 1.2 ml of trypsin inhibitor (T6522, Sigma, 1 mg ml^−1^) was added and incubated for a further 1 min. Then, 750 µl of DNase (DN25, Sigma, 10 mg ml^−1^) was added to digest sticky DNA. Samples were centrifuged at 400*g* for 5 min. The supernatant was discarded, and the pellet was triturated with 5 ml of microglia culture media (DMEM (11995065, Gibco), 10% heat-inactivated FBS (10270106, Gibco), 100 U ml^−1^ penicillin–streptomycin) using a 1-ml pipette tip. Homogenate was centrifuged again at 400*g* for 5 min, the supernatant was aspirated and the pellet was re-suspended in 5 ml of culture media. Cell density was determined using a haemocytometer. Cells were plated in poly-d-lysin-hydrobromid-coated (P6407, Sigma) T-75 flasks at a density of 50,000 cells per mm^2^ (approximately 3–4 million cells per flask). Cells were incubated in a cell culture incubator with 5% CO_2_, 100% humidity and 37 °C. The following day, the cell culture medium was replaced to remove dead cells and debris. Then, the cell culture medium was changed every 5 days. On day 10, microglia were collected through vigorously tapping the flasks and collecting the floating cells in the medium. The resulting cells were more than 95% microglia and were used for downstream experiments.

### In vitro ganglioside stimulation

GM1 (Cay19579, Biomol), GM2 (G8397, Sigma) or GM3 (860058P, Sigma) was dissolved in chloroform:methanol (2:1; 6340.1, 8388.1, Roth) and stored at −80 °C. On the day of the experiment, gangliosides were diluted with 2-propanol (20842.312, VWR) to reach the desired concentrations and added to 96-well plates. Coating was achieved through evaporating. Then, primary microglia were added at a density of 1 × 10^4^ cells per well and incubated for 16 h. The supernatant was subjected to cytokine measurement. Microglia cells were fixed in 4% formalin. For MGL blocking assay, primary microglia were pretreated with anti-MGL antibody (HM1081, 10 µg ml^−1^, Hycult Biotech), isotype control IgG (02-9688, 10 µg ml^−1^, Thermo Fisher), GalNAc (A2795, 50 mM, Sigma) or EGTA (3054.1, 10 mM, Roth) for 30 min at 37 °C before the ganglioside stimulation.

### Cytokine and chemokine measurement

IL-1α, IL-1β, IL-6, IL-10, IL-12p70, IL-17A, IL-23, IL-27, MCP-1, IFNβ, IFNγ and TNF as well as CCL2, CCL3, CCL4, CCL5, CCL11, CCL17, CCL22, CXCL1, CXCL5, CXCL9, CXCL10 and CXCL13 in the supernatants of stimulated primary microglia were quantified using the LEGENDplex Mouse Inflammation Panel (13-plex) (BioLegend, 740446) and the LEGENDplex Mouse Proinflammatory Chemokine Panel (13-plex) (BioLegend, 740451) according to the manufacturer’s instructions. Data were acquired using a BD LSRFortessa (Becton Dickinson) and analysed with LEGENDplex Data Analysis Software Suite (BioLegend).

### In vitro exocytosis inhibitor treatment

Primary microglia were generated as described above, plated at a density of 1 × 10^4^ cells per well into a 96-well plate and incubated for 24 h in a cell culture incubator. Then, medium was removed and replaced with golgicide A (from 10 mM stock in DMSO, HY-100540, MedChemExpress), vacuolin-1 (from 10 mM stock in DMSO, HY-118630, MedChemExpress), ionomycin (from 5 mM stock in DMSO, I24333, Invitrogen), brefeldin A (from 50 mM stock in DMSO, HY-16592, MedChemExpress), thapsigargin (from 50 mM stock in DMSO, HY-13433, MedChemExpress), BAPTA-AM (from 50 mM stock in DMSO, HY-100545, MedChemExpress) or EGTA (from 0.5 M stock in ddH_2_0, pH 7.5, 3054.1, Roth) diluted in microglia medium, and incubated for 4 h. After incubation, supernatant was removed and Hex activity assay was performed. Baseline values from a medium-only control were subtracted from all other values and then normalized to the untreated, DMSO-only condition. Cell viability was tested FACS-based for all conditions and viability greater than 90% was confirmed.

### Primary NPC culture and Hex uptake assay

A single-cell suspension from P2 newborn mouse pups was obtained as described above. Cells were plated at a density of 0.5–1 × 10^4^ cells per well in a poly-l-lysine (A-005-C, Merck)- and laminin (11243217001, Merck)-coated 96-well plate in neuron medium^84^ (Neurobasal medium (21103049, Gibco), 1x B-27 supplement (17504044, Gibco), 2 mM glutamine (G7513, Sigma), 100 U ml^−1^ penicillin–streptomycin). Cells were incubated at 5% CO_2_, 100% humidity and 37 °C. The following day, half of the cell culture medium was replaced. Then, the cells were fed every 2–3 days through a half-medium change.

For Hex uptake assays, half of the medium was removed and replaced with neuron medium containing compounds at 2× final concentrations: EIPA (final 25 µM; from 25 mM stock in DMSO), Wortmannin (final 1 µM; from 10 mM stock in DMSO) or M6P (final 10 mM; prepared in neuron medium). After 1 h of preincubation, recombinant His-tagged HEXB (HY-P75808, MedChemExpress) was added to a final concentration of 100 nM and incubated for 6 h. Following incubation, supernatant was removed and cells were washed four times with PBS and subjected to immunoblotting or Hex activity assay as described above.

For conditioned media experiments, fresh medium from primary wild-type microglia cultures was collected and Hex activity was quantified. Where indicated, heat inactivation was performed by incubating the medium at 95 °C for 5 min. Conditioned medium was diluted 1:1 with fresh neuron medium, and a half-medium change was performed. Cells were incubated for the indicated durations, then washed, lysed and subjected to Hex activity assay.

For Transwell assays, NPCs were plated at a density of 0.1 × 10^6^ cells and maintained for 4 days. A half-medium change was then performed, a Transwell insert with 0.4-µm pore size (3470, Corning) was added and 1 × 10^4^ primary microglia were seeded into the insert in neuron medium. Microglia were preincubated for 4 h with either DMSO-containing neuron medium or 15 µM brefeldin A (from 50 mM stock in DMSO) before transfer. After 24 h of co-culture, the insert was removed, and neurons were washed four times with PBS, lysed and subjected to Hex activity assay.

For lipidomic analysis of cultured NPCs, cells were plated at 0.5 × 10^6^ in a six-well plate. After 4 days, conditioned media was added and the cells were incubated for 48 h without further medium change before cell lysis and lipid extraction (see above).

### Primary fibroblast culture and Hex uptake assay

Primary fibroblast cultures from *Hexb*^*−/−*^ mice were established as previously described^85^. In brief, mice were anaesthetized and transcardially perfused with ice-cold PBS, and ears and approximately 5 cm of the tail were cut and placed in ethanol (20821.310, VWR) for 5 min. Ears and tails were cut into small pieces and each was incubated in 2 ml of digestion mix (4 ml 2.5 mg ml^−1^ collagenase D (11088858001, Merck) plus 0.25 ml of 20 mg ml^−1^ pronase (10165921001, Merck)) for 90 min at 37 °C on a shaker at 200 rpm. After incubation, ears and tails were placed in a 70-µm cell strainer and into a 10-cm dish filled with 10 ml of media (RPMI1640 GlutaMAX supplement (61870036, Gibco), 10% FBS, 50 µM 2-mercaptoethanol (31350010, Gibco), 1x MEM Non-Essential Amino Acids Solution (M7145, Gibco), 100 U ml^−1^ penicillin–streptomycin). Tissue was ground using a 10-ml syringe plunger. The cell suspension was centrifuged and washed twice with media. Pellets from ears and tails were re-suspended in 10 ml of media each, 10 µl of amphotericin B (15290018, Gibco) was added and cells were plated into 10-cm cell culture dishes and incubated in a cell culture incubator at 5% CO_2_, 100% humidity and 37 °C. On the third day, medium was replaced to remove debris. Every consecutive 3 d, fibroblasts were split: Plates were washed once with PBS and incubated for 5 min at 37 °C with 2 ml of trypsin-EDTA solution (25300054, Gibco). Plates were gently tapped, 10 ml of fresh medium was added and the cell suspension was centrifuged at 450*g*. Pellets were re-suspended in fresh medium, counted and seeded at a density of 2 × 10^5^ cells in new 10-cm dishes. For HEXB uptake assays, fibroblasts were seeded in a 96-well plate at a density of 1 × 10^4^ cells per well and incubated for 24 h in a cell culture incubator. Then, medium was removed and medium supplemented with recombinant His-tagged HEXB (HY-P75808, MedChemExpress) was added and incubated for 6 h. Following incubation, supernatant was removed, and cells were gently washed four times with PBS and subjected to immunoblotting or Hex activity assay as described above. To inhibit pinocytosis, cells were preincubated for 1 h with one of the following compounds added to the culture medium: EIPA (25 µM; from 25 mM stock in DMSO), Wortmannin (1 µM; from 10 mM stock in DMSO), M6P (10 mM; prepared in neuron medium), IGF2R-blocking antibody (20 µg ml^−1^; AF2447, R&D Systems) or isotype control IgG (20 µg ml^−1^; AB-108-C, R&D Systems). Following preincubation, recombinant His-tagged HEXB was added to a final concentration of 100 nM, and cells were incubated for a further 6 h. Cell viability was tested FACS-based for all conditions and viability greater than 90% was confirmed.

### Organotypic hippocampal slice culture

Organotypic hippocampal slice cultures (OHSCs) were prepared from newborn P2–3 mice as previously described^86^. In brief, mice were decapitated, the brains were removed and placed into ice-cold cutting solution (HBSS (24020117, Gibco), 10 mM HEPES (15630080, Gibco), 35 mM glucose, 100 U ml^−1^ penicillin–streptomycin (15140122, Gibco)) and the hippocampi from both hemispheres were isolated. Isolated hippocampi were cut into 350-μm-thick slices using a tissue chopper (McIlwain) and transferred to 0.4-μm culture plate inserts (PICM03050, Millipore), and placed in six-well plates containing 1 ml of culture medium (0.5 × minimum essential medium (21090022, Gibco), 25% heat-inactivated horse serum (26050088, Gibco), 25% BME basal medium (21010046, Gibco), 2 mM GlutaMAX (35050061, Gibco), 0.65% glucose (G8769, Sigma) and 100 U ml^−1^ penicillin–streptomycin (15140122, Gibco)) per well. Slices were incubated in a cell culture incubator at 5% CO_2_, 100% humidity and 35 °C. The culture medium was changed on the first day after preparation and every 2 consecutive days.

### Microglia replacement in OHSCs

Microglia were depleted and replenished as described before^87^. Briefly, microglia were depleted using the macrophage toxin clodronate (233183, Merck-Millipore). Clodronate was solved in autoclaved H_2_O with a concentration of 1 mg ml^−1^. Freshly prepared OHSCs were incubated with 100 μg of clodronate per ml of OHSC culture medium for 24 h. Subsequently, clodronate was replaced with fresh culture medium. Microglia-depleted OHSCs were kept for 10 days before microglia replenishment. Medium was changed every 2 consecutive days. Primary microglia were isolated as described above. After collection of primary microglia, they were re-suspended in OHSC culture medium to a final density of 1,000 cells per μl. Then, 2,000 cells were added on top of each microglia-free hippocampal slice. Cells were allowed to engraft for 7 days before further analysis.

### Immunocytochemistry

Primary cells were fixed with 4% formalin for 15 min at room temperature, blocked and permeabilized with 5% BSA in PBS supplemented with 0.1% Triton X-100 for 1 h at room temperature. Then, the cells were incubated overnight at 4 °C with anti-TuJ1 (1:500, 302 306; Synaptic Systems), and/or anti-His (1:1,000, MA1-21315, Invitrogen), and/or anti-LAMP1 (1:500, PA1-654A, Invitrogen), and/or anti-Vimentin (1:100, 5741, Cell Signaling) diluted with 5% BSA in PBS, followed by incubation with secondary antibodies goat-anti-chicken IgY (H+L), Alexa Fluor 488 (1:500, A11039, Invitrogen), and/or donkey anti-rabbit IgG (H+L), Alexa Fluor 488 (1:500, A21206, Invitrogen), and/or donkey anti-rabbit IgG (H+L), Alexa Fluor 568 (A10042, Invitrogen), and/or donkey anti-mouse IgG (H+L), Alexa Fluor 647 (1:500, A31571, Invitrogen) diluted in 5% BSA in PBS for 2 h at 4 °C. For nuclear counterstaining, DAPI was added for 30 min at room temperature. Finally, the cells were visualized with the TCS SP8 X (Leica) confocal microscope using a ×63 objective.

### Isolation of adult neurons from mouse brain cortices

To isolate cortical neurons from adult mice, mice were anaesthetized and transcardially perfused with ice-cold PBS. The brain was taken out and brain cells isolated as described previously^88^. A small piece of cortical tissue (grain of rice) was dissected and finely minced. Tissue was incubated in 10 ml of enzyme digestion solution (75 μl of Papain suspension (LS003126, Worthington) diluted in enzyme stock solution (10 ml of 10 × EBSS (E7510, Sigma-Aldrich), 2.4 ml of 45% glucose (G8769, Sigma-Aldrich), 5.2 ml of 1 M NaHCO_3_ (AAJ62495-AP, Fisher Scientific), 200 μl of 0.5 M EDTA (15575020, Invitrogen) and 168.2 ml of ddH_2_O, filter-sterilized through a 0.22-μm filter)) and equilibrated to 37 °C. Samples were shaken for 30–40 min in a water bath at 37 °C. Enzymatic digestion was stopped with 1 ml of 10 × hi ovomucoid inhibitor solution (300 mg of BSA (8076.3, Roth), 300 mg of ovomucoid trypsin inhibitor (LS003086, Worthington) diluted in 10 ml of PBS and filter-sterilized using a 0.22-μm filter) and 20 μl of 0.4% DNase (LS002007, Worthington) diluted in 10 ml of inhibitor stock solution (50 ml of 10 × EBSS (E7510, Sigma-Aldrich), 6 ml of 45% glucose (G8769, Sigma-Aldrich), 13 ml of 1 M NaHCO_3_ (AAJ62495-AP, Fisher Scientific) diluted in 170.4 ml of ddH_2_O and filter-sterilized through a 0.22-μm filter). Cells were centrifuged at 500*g*, 5 min, 4 °C. Supernatant was discarded and the pellet was subjected to neuron isolation using the Adult Neuron Isolation Kit, mouse (130-126-602, Miltenyi Biotec) following the manufacturer’s instructions.

### Isolation of exosomes from cell culture supernatant

To remove cells, cell debris and larger vesicles, cell culture supernatant was serially centrifuged at 300*g* for 10 min, 2,000*g* for 30 min and 10,000*g* for 45 min. From the remaining supernatant, exosomes were isolated using the Pan EV isolation kit (130-117-039, Miltenyi Biotec) following the manufacturer’s recommendations.

### BLZ945 treatment

BLZ945 hydrochloride (HY-12768A, MedChemExpress) was dissolved in 20% (2-hydroxypropyl)-β-cyclodextrin (H107, Sigma-Aldrich). In adult mice, a dose of 200 mg per kg body weight was applied by oral gavage for 7 consecutive days. Neonates received intraperitoneal injections at P7, P9, P11 and P13.

### Bone marrow transplantation and microglia replacement

To deplete endogenous microglia, mice received BLZ945 for 7 consecutive days. On the day of the transplantation, the last dose was applied. In parallel, mice were treated with neomycin (1.1 g l^−1^; N6386, Sigma) acid water (pH 2.5) to reduce the risk of infection. Recipient mice were lethally irradiated with 9 Gy using a RS2000 X-ray irradiator (Rad Source Technologies). *Cx3cr1*^*GFP*^ mice served as bone marrow donors. Bone marrow was isolated from the tibias and femurs by flushing with PBS. After red blood cell removal, cells were washed, counted and re-suspended in an appropriate volume of PBS (1 × 10^7^ cells per 100 µl). Within 2 h after irradiation, adult mice received donor bone marrow (1 × 10^7^ cells) through tail vein injection. Neonates were intraperitoneally injected with the same amount of donor bone marrow cells. Following the injection, treatment with neomycin acid water was continued for another 2 weeks. At 4 weeks after transplantation, mice were subjected to blood withdrawal from the facial vein to control for a proper reconstitution with donor-derived peripheral blood cells.

### Intracerebroventricular injections

Mice were anaesthetized and 2 µl of antibody solution (100 µg ml^−1^; anti-MGL antibody (HM1081, Hycult Biotech) or isotype control IgG (02-9688, Thermo Fisher)) was injected into the lateral ventricle twice weekly for 3 weeks in total, starting at P10. At 3 days after the last injection, mice were anaesthetized and transcardially perfused with ice-cold PBS. The brain was taken out, microglia were isolated as described above and an equal amount of brain tissue was homogenized using a tissue homogenizer in lysis buffer (50 mM HEPES pH 7.4, 40 mM NaCl, 2 mM EDTA, 1.5 mM NaVO_4_, 30 mM NaF, 10 mM sodium pyrophosphate, 10 mM sodium beta glycerophosphate and protease inhibitors (Sigma-Aldrich, A32965)) supplemented with 1% Triton X-100.

### Patch-clamp electrophysiology in acute brain slices

P28-42 mice were deeply anaesthetized with isoflurane (5%) in oxygen-enriched air (Oxymat 3, Weinmann), and decapitated into carbonated, ice-cold slicing solution. A Leica VT 1200S vibratome was used to obtain 350-µm-thick coronal slices from motor cortex. Slices were directly transferred to carbogenated slicing solution at 33 °C for 10 min, and then further transferred to carbogenated standard artificial cerebrospinal fluid at room temperature. After 30–60 min of recovery time, slices were used in whole-cell patch-clamp experiments. Slicing solution contained (in mM) 93 NMDG, 93 HCl, 2.5 KCl, 1.2 NaH_2_PO_4_, 30 NaHCO_3_, 20 HEPES, 25 glucose, 5 sodium ascorbate, 2 thiourea, 3 sodium pyruvate, 10 MgSO_4_ and 0.5 CaCl_2_ and was calibrated to a pH of 7.3–7.4 and an osmolarity of 315 mOsm. Standard ACSF contained (in mM) 125 NaCl, 3 KCl, 1.25 NaH_2_PO_4_, 26 NaHCO_3_, 10 glucose, 1 MgCl_2_ and 2 CaCl_2_ and was calibrated to an osmolarity of 315 mOsm. For recordings, slices were held in a chamber at 33 °C and perfused with ACSF (2–4 ml min^−1^). Cells were visualized for patching using differential interference contrast microscopy (Scientifica) with a water immersion objective (Olympus LUMPlanFLN40xW) and a CCD camera (Scientifica SciCam Pro). Cells were recorded in whole-cell patch-clamp mode using pipettes pulled from standard-wall borosilicate capillaries (3.5–6 MOhm, DMZ Zeitz-Puller). Intracellular solution contained (in mM) 140 K-gluconate, 10 KCl, 10 HEPES, 4 Naphosphocreatine, 4 ATP-Mg, 0.4 GTP and biocytin (4 mg ml^−1^) and was calibrated to pH 7.3 with KOH and an osmolality of 290–300 mOsm. A Multiclamp 700B amplifier (Axon Instruments) was used for whole-cell voltage-clamp or current-clamp recordings, together with a Digidata1550 (Molecular Devices) for digitization. Recordings were low-pass filtered at 10 kHz using a Bessel filter and digitized at 50 kHz. Series resistance was routinely compensated in voltage-clamp, and recordings were excluded if access resistance exceeded 30 MOhm.

#### Intrinsic electrophysiological properties

L2/3 pyramidal cells were identified morphologically in *Hexb*^*+/−*^ and *Hexb*^*−/−*^ transgenic mice. Input resistance was obtained from current traces evoked by a hyperpolarizing step (10 mV, 100 ms) and resting potential was determined in current-clamp mode. Spiking profiles were recorded in current-clamp configuration (membrane potential was kept at −70 mV by passing a holding current) and the threshold current for spiking was assessed by successive current steps (starting at −150 pA and increased by 20 pA every sweep of 1-s duration). For characterizing action potential parameters, we used a previously developed pipeline^89^.

#### Connectivity

Spontaneous excitatory postsynaptic currents (EPSCs) were sampled for 5 min, digitally filtered at 1 kHz and detected offline. For analysis, a low-pass Butterworth filter was applied with a cut-off frequency of 500 Hz. The amplitude and area thresholds for detection were 4 pA and 50 pA x ms. All events were manually validated, and artifacts were discarded by visual inspection.

#### Statistical analysis

Data are presented as mean ± s.e.m. Normality was assessed using Shapiro–Wilk test, D’Agostino & Pearson omnibus test and Kolmogorov–Smirnov test, at a significance level of 0.05. A distribution was considered normal if all tests were passed. When a dataset did not satisfy normality criteria, non-parametric statistics were applied. Two-tailed Mann–Whitney test was used for single comparisons. For normal distributions, homoscedasticity was assessed using *F* test, at a significance level of 0.05. For homogeneous variances, two-tailed *t*-test was used for single comparisons.

### Gene expression analysis

RNA was isolated with the Arcturus PicoPure RNA Isolation Kit (KIT0204, Life Technologies) according to the manufacturer’s protocol. Reverse transcription and real-time quantitative PCR analysis were performed using high capacity RNA-to-cDNA-Kit and Gene Expression Master Mix reagents (4387406, 4369510, Applied Biosystems) according to the manufacturer’s recommendations. The following TaqMan Gene Expression Assays were used: Actb (Mm01205647_g1), Gfap (Mm01253033_m1), Itgam (Mm00434455_m1), Syt1 (Mm00436858_m1), Plp1 (Mm01297210_m1), Hexb (Mm01282432_m1), Ccl5 (Mm01302427_m1), Cx3cl1 (Mm00436454_m1), Il1b (Mm00434228_m1) and Il18 (Mm004344226_m1). Quantitative PCRs were run on a LightCycler 480 (Roche).

### Serum analysis

Serum (100–200 μl) was analysed for liver and renal parameters at SYNLAB Vet, Augsburg. TNF and IL-6 were analysed using the TNF and IL-6 ProQuantum Immunoassay Kits (A43656, A43658, Invitrogen) following the manufacturer’s instructions.

### Statistics and reproducibility

Statistical significance was determined using GraphPad Prism v.10.2.2 software. *P* < 0.05 was considered statistically significant. All quantification experiments were performed in a blinded manner by assignment of unidentifiable numbers to mice, tissues and images for data acquisition and processing. Data labels and groups were reinstated only for statistical analysis. Quantification and imaging were not repeated following statistical analysis.

Ganglioside stimulation and slice culture experiments (Fig. 4f–h and Extended Data Figs. 5a–c and 7q–u) were independently repeated twice with consistent results; representative data from one experiment are shown.

Experiments involving treatment of primary microglia with secretion inhibitors and Hex uptake assays in neuron and fibroblast cultures (Fig. 5i,k,n and Extended Data Fig. 7d–f,j,m–o) were also independently repeated twice with similar outcomes; data from both replicates were pooled and presented in the respective graphs.

Representative immunofluorescence and immunohistochemical micrographs are shown from multiple replicates (Fig. 4g: four technical replicates; Fig. 7a: two biological replicates; Extended Data Fig. 7p: four technical replicates).

### Reporting summary

Further information on research design is available in the Nature Portfolio Reporting Summary linked to this article.

## Online content

Any methods, additional references, Nature Portfolio reporting summaries, source data, extended data, supplementary information, acknowledgements, peer review information; details of author contributions and competing interests; and statements of data and code availability are available at 10.1038/s41586-025-09477-y.

## Supplementary information

Supplementary Information This file contains Supplementary Figs. 1–5 and legends for Supplementary Tables 1–22.

Reporting Summary

Supplementary Tables This file contains Supplementary Tables 1–22.

## Source data

Source Data Fig. 1, 2, 4, 5 and 6 and Extended Data Figs. 2, 4, 5, 6, 7, 8 and 9.

## Data Availability

All data of this manuscript are openly available. The raw and processed sequencing data for this project are available under GSE300762 (related to Fig. 1 and Extended Data Fig. 3g–k), GSE300359 (related to Fig. 3), GSE300356 (related to Fig. 7), GSE300357 (related to Extended Data Fig. 8) and GSE300355 (related to Supplementary Fig. 1). These include raw fastq files, filtered feature-barcode matrices and analysed Seurat objects. The underlying data supporting the sequencing results (for example, volcano plots, heat maps) are provided in Supplementary Tables 2–22. Lipidomic datasets are also included in the Supplementary Tables. Raw western blot images are provided in the Supplementary Information. Source data are provided with this paper.

## Code availability

No custom code was used within the study.
