# Supplementary material for: Microglia–neuron crosstalk through Hex–GM2–MGL2 maintains brain homeostasis
Source: Nature. 2025 Aug 6;646(8086):913–24. doi: 10.1038/s41586-025-09477-y (PMC12545202; doi:10.1038/s41586-025-09477-y)
Supplement: Supplementary file 1 — This file contains Supplementary Figs. 1–5 and legends for Supplementary Tables 1–22. [file 41586_2025_9477_MOESM1_ESM.pdf]

nature portfolio

https://doi.org/10.1038/s41586-025-09477-y

Supplementary information

Microglia–neuron crosstalk through Hex–GM2–MGL2 maintains brain homeostasis

In the format provided by the
authors and unedited

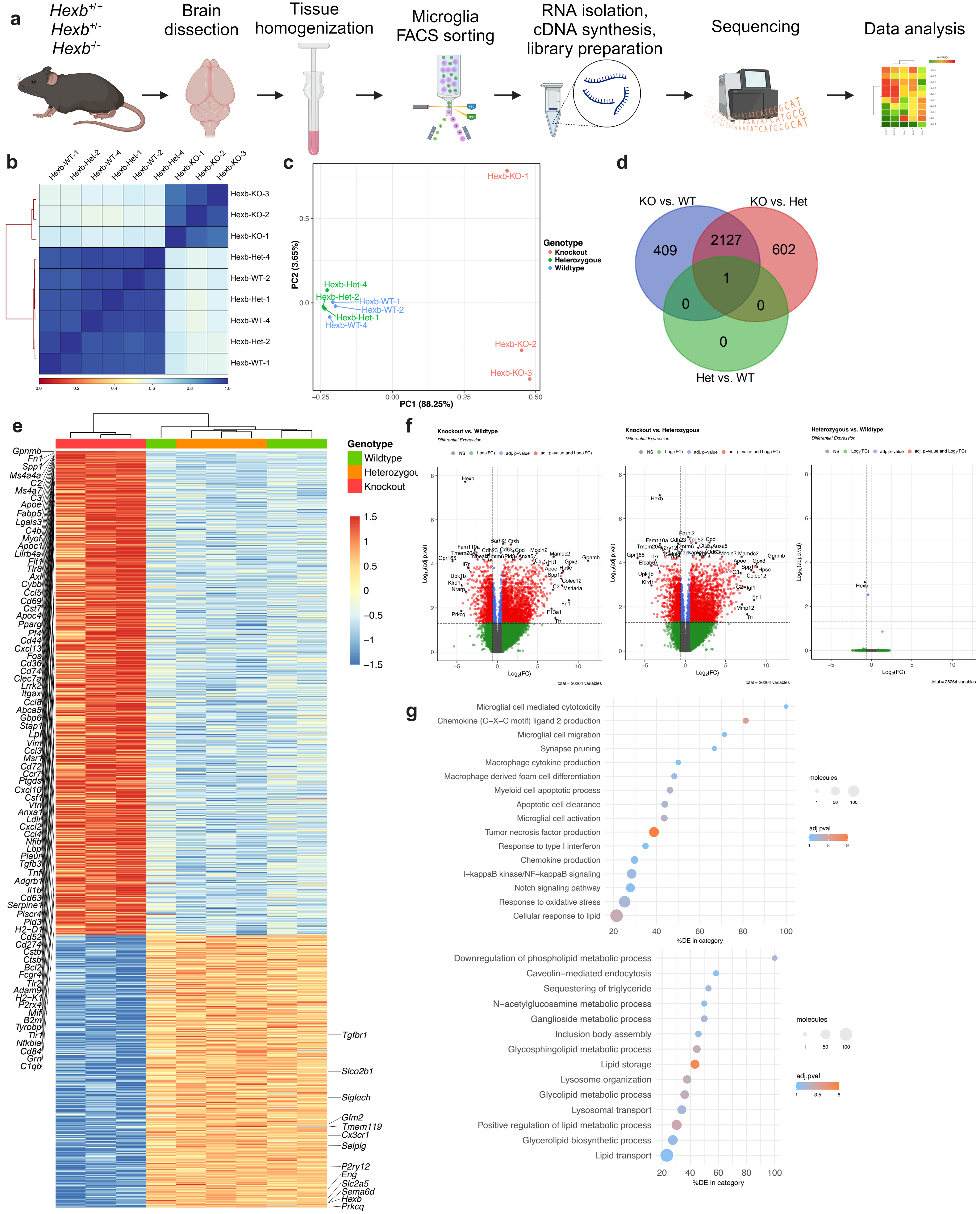

Supplementary Figure 1: bulkRNA-seq analysis of *Hexb*-deficient microglia.
a, Experimental workflow. **b-d**, Spearman correlation plot, PCA plot, and venn diagram emphasizing distinct gene signature in *Hexb*$^{-/-}$ mice. **e**, Heat map presenting differentially expressed genes (DEGs). Colors in the heat map correspond to normalized expression values. **f**, Volcano plots comparing different genotypes. **g**, GO term enrichment analysis of DEGs between *Hexb*$^{-/-}$ and *Hexb*$^{+/+}$

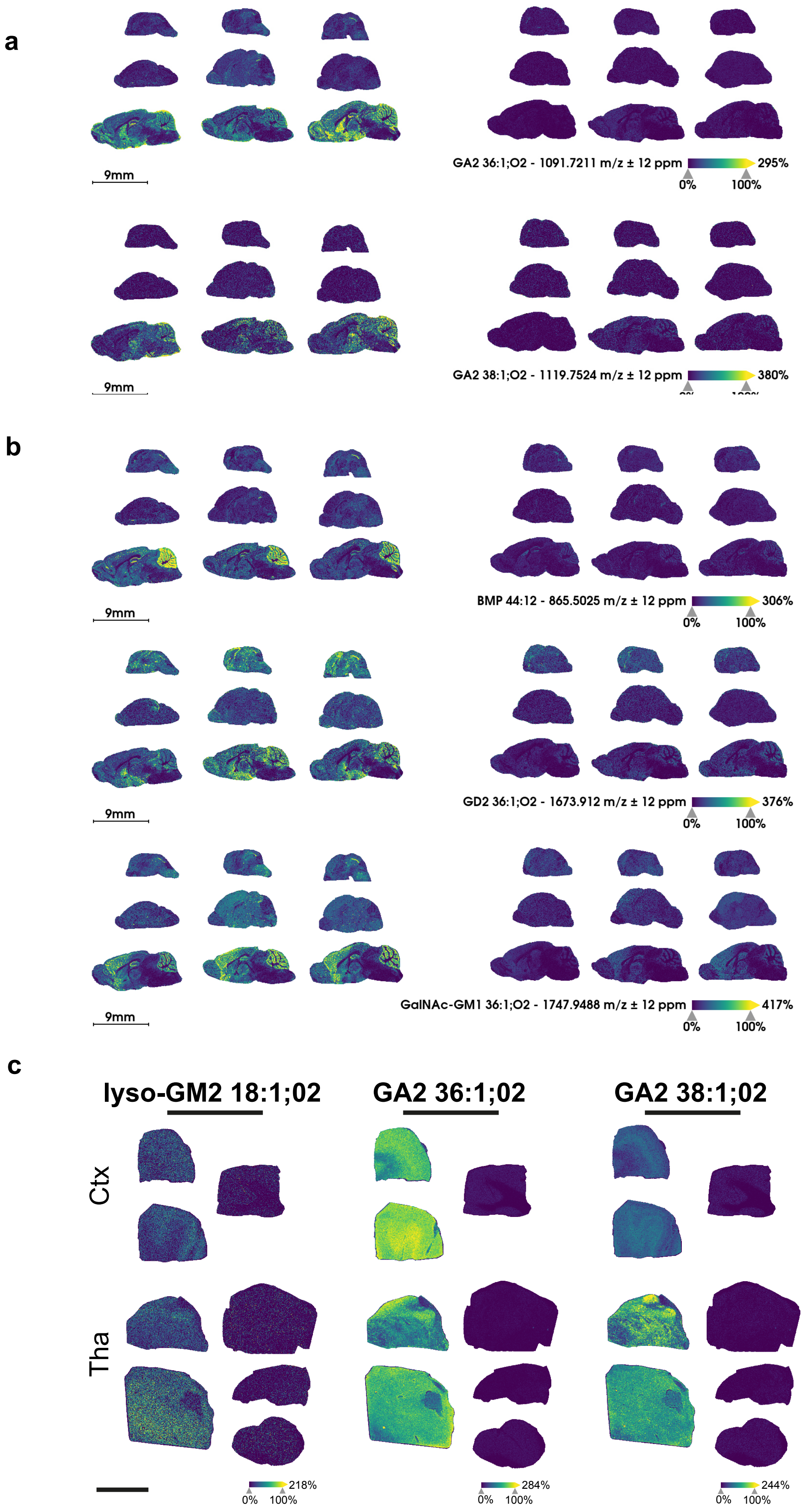

Supplementary Figure 2: Analysis of ganglioside storage in a temporospatial manner.
a-b, MALDI MSI on brains of *Hexb*$^{-/-}$ and *Hexb*$^{+/-}$ controls at P0 (upper row), P7 (mid row), and P120 (bottom row). For each indicated ganglioside, ion images from brains of *Hexb*$^{-/-}$ (left) and *Hexb*$^{+/-}$(right) controls are shown. Color scale represents a visual map of the intensities (in arbitrary units) of the ion images. Scale bar 9 mm. **c**, MALDI MSI on brains (cortex and thalamus) of Sandhoff disease patients and unaffected controls. For each indicated ganglioside, ion images from Sandhoff disease patients (left) and unaffected controls (right) are shown. Color scale represents a visual map of the intensities (in arbitrary units) of the ion images. Scale bar 9 mm.

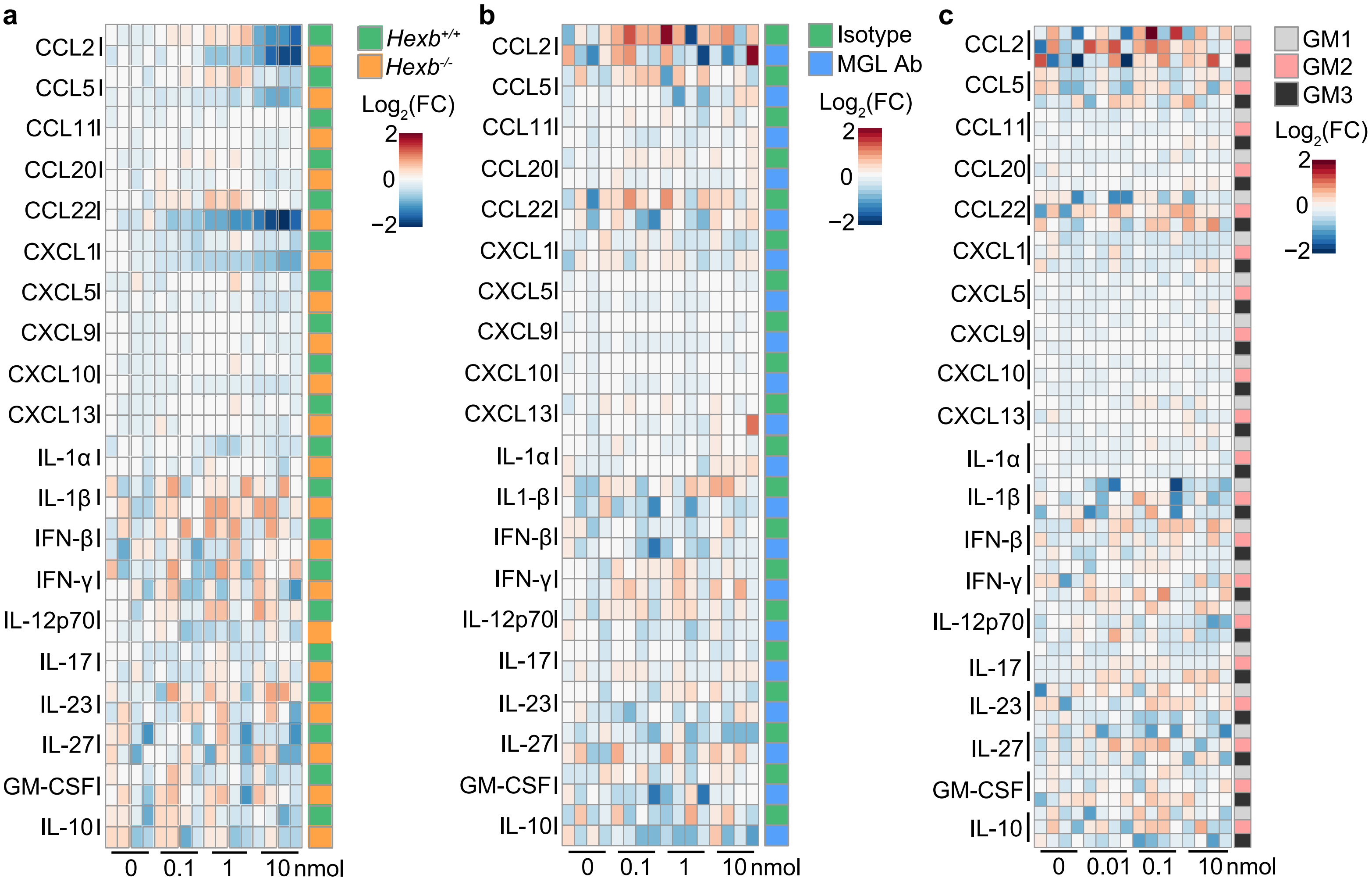

Supplementary Figure 3: Microglial cytokine and chemokine secretion upon ganglioside stimulation.

a-c, Fold changes of indicated cytokines normalized to the unstimulated condition. Data referring to **Figure 4f-g**. One-way ANOVA followed by Dunnett's test for correcting multiple comparisons was used to test the indicated conditions versus the unstimulated condition.

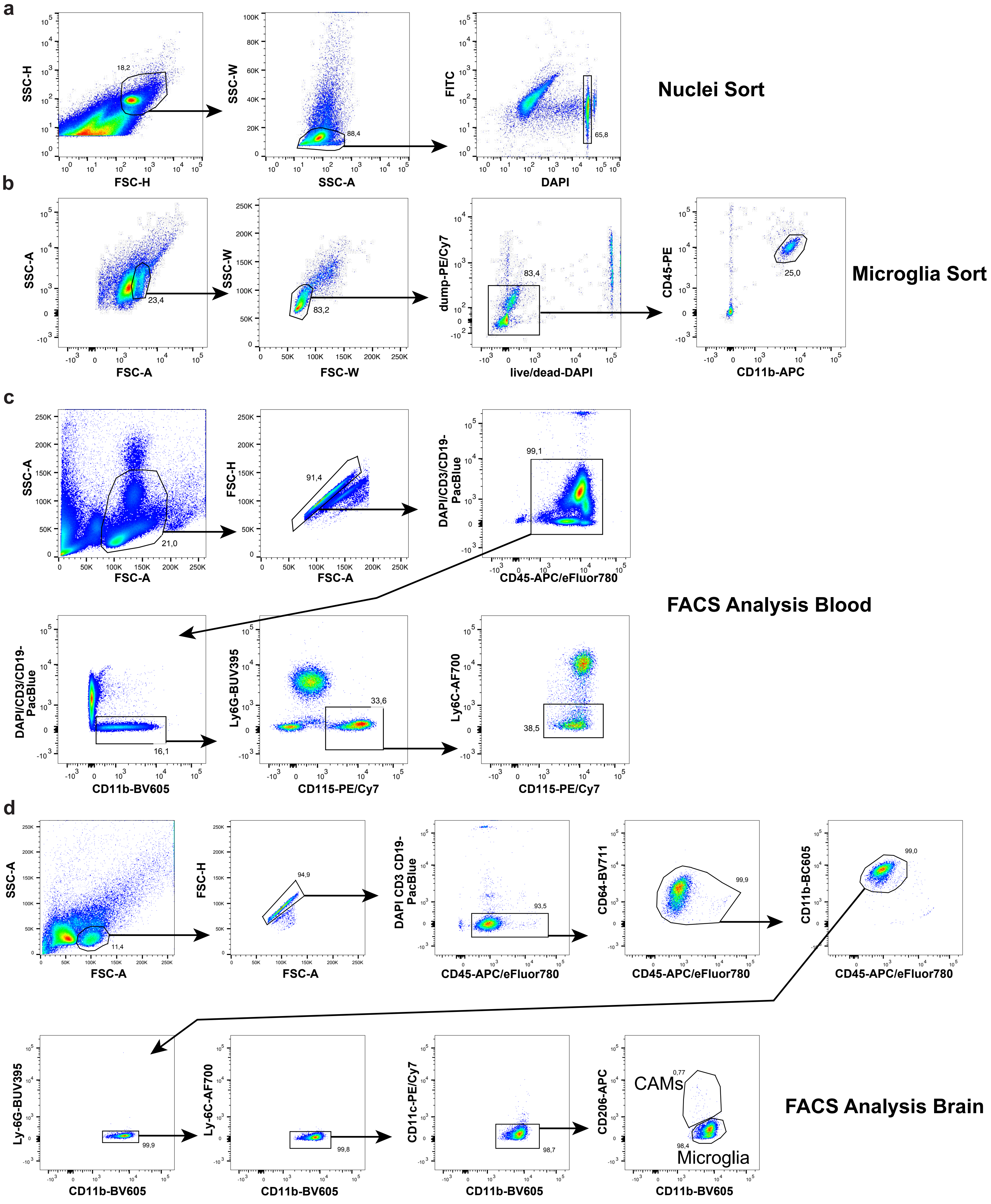

Supplementary Figure 4: FACS gating strategies.

a, Gating for single DAPI+ nuclei for snRNA-seq. **b**, Gating for single viable microglia (Lin$^-$CD45^{int}CD11b$^+$) for bulkRNA-seq. Lineage marker include Ly6C, Ly6G, B220, and CD3e. **c**, Gating for single viable blood monocytes (CD3$^-$CD19$^-$CD45$^+$CD11b$^+$CD115$^+$). Ly6C^{lo} monocytes were analyzed for GFP expression. **d**, Gating for single viable microglia (CD3$^-$CD19$^-$Ly6G$^-$Ly6C$^-$CD11c$^-$CD206$^-$CD45$^+$CD64$^+$CD11b$^+$) to analyze GFP expression.

a

Brightfiled (ladder) **TSG101**

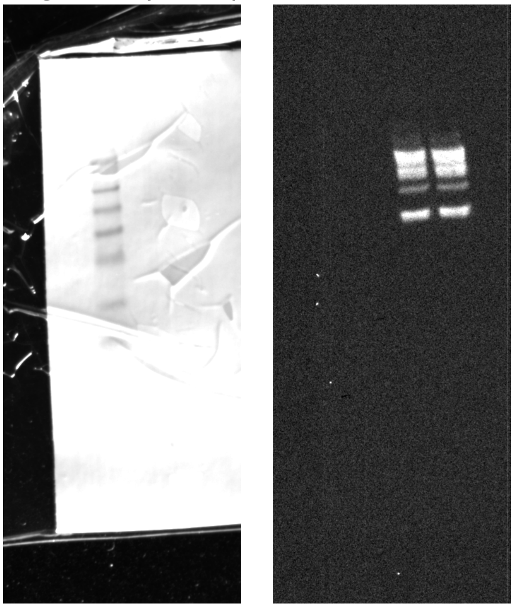

Gel from ED Figure 7b

Lane 1-2-3:
whole supernatant - exosomes - exosome-depleted supernatant

b

Brightfiled (ladder) **His and GAPDH**

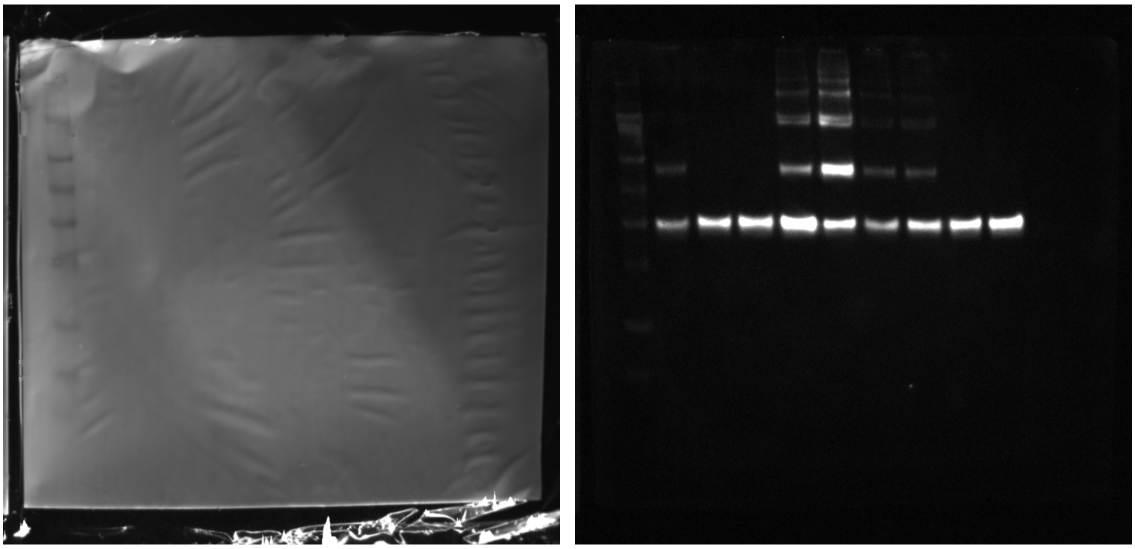

Gel from ED Figure 7j

His

GAPDH

Lane 2-3-4-5-6-7-8-9:
untreated-untreated-250nM-250nM-100nM-100nM-1nM-1nM

Lane 1: not shown in ED Figure 7

c

Brightfiled (ladder) **His and GAPDH**

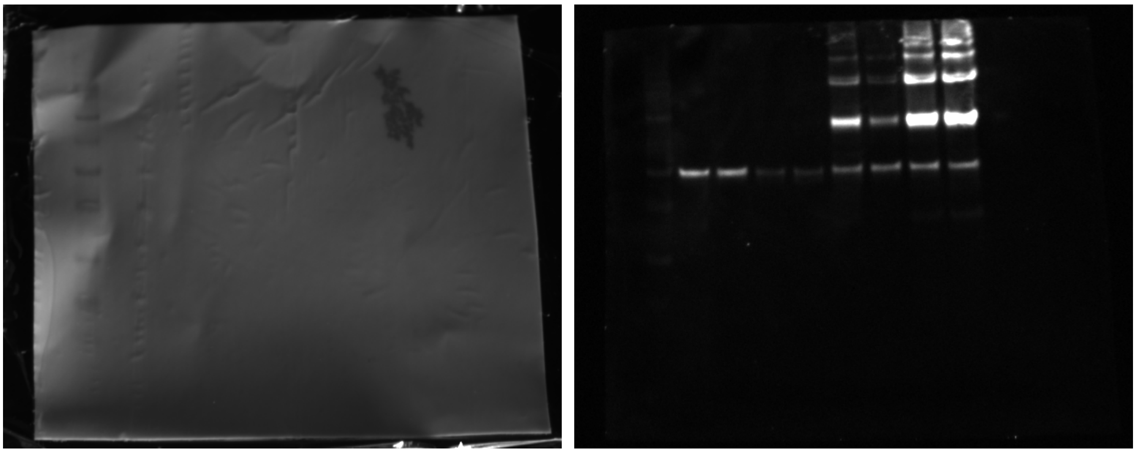

Gel from ED Figure 7m

His

GAPDH

Lane 1-2-3-4-5-6-7-8:
untreated-untreated-1nM-1nM-100nM-100nM-250nM-250nM

Supplementary Figure 5: Full gel and blot scans.
a-c, Full scans of western blot images referring to **Extended Data Figures 7b,j,m**.

SUPPLEMENTARY TABLES

Supplementary Table 1: Characteristics of the patient samples analyzed in the present study.
Supplementary Table 2: Marker genes for the annotated UMAP in **Figure 3a**.
Supplementary Table 3: Marker genes for the immune cell populations in **Figure 3b**.
Supplementary Table 4: Marker genes for the microglia cell cluster in **Figure 3c**.
Supplementary Table 5: Top 10 microglial marker genes per cluster used for the heatmap in **Figure 3d**.
Supplementary Table 6-8: Differentially expressed genes between the indicated microglia cluster as shown in the volcano plots in **Figure 3e**.
Supplementary Table 9: Differentially regulated lipids between the indicated genotypes and conditions as depicted in Figure **4a** and **7g,h** and **Extended Data Figure 4a** and **8g**.
Supplementary Table 10: Differentially regulated lipids between the indicated conditions as depicted in **Figure 5m** and **Extended Data Figure 7k**.
Supplementary Table 11: Marker genes for the annotated UMAP in **Figure 7b**.
Supplementary Table 12: Marker genes for the myeloid cell population in **Figure 7c.**
Supplementary Table 13: Top 10 microglial marker genes per cluster used for the heatmap in **Figure 7d**.
Supplementary Table 14-15: Differentially expressed genes between the indicated microglia cluster as shown in the volcano plots in **Figure 7e**.
Supplementary Table 16: Genes contained in the species comparison between human and mouse cells in **Figure 7f**.
Supplementary Table 17: Differentially expressed genes in the bulkRNA dataset as analyzed in **Suppl. Figure 4**.
Supplementary Table 18: Marker genes for the annotated UMAP in **Extended Data Figure 8m**.
Supplementary Table 19: Marker genes for the annotated UMAP in in **Extended Data Figure 8n**.
Supplementary Table 20: Marker genes for the annotated UMAP in **Extended Data Figure 8o** and the heatmap in **Extended Data Figure 8s**.
Supplementary Table 21: Differentially expressed genes between the indicated microglia cluster as shown in the volcano plot in **Extended Data Figure 8t**.
Supplementary Table 22: Differentially regulated lipids between the indicated genotypes and conditions as depicted in **Extended Data Figure 9k**.
