# Supplementary material for: Microglia–neuron crosstalk through Hex–GM2–MGL2 maintains brain homeostasis
Source: Nature. 2025 Aug 6;646(8086):913–24. doi: 10.1038/s41586-025-09477-y (PMC12545202; doi:10.1038/s41586-025-09477-y)
Supplement: Supplementary file 2 — Reporting Summary [file 41586_2025_9477_MOESM2_ESM.pdf]

nature portfolio

Corresponding author(s): Marco Prinz

Last updated by author(s): Jun 20, 2025

Reporting Summary

Nature Portfolio wishes to improve the reproducibility of the work that we publish. This form provides structure for consistency and transparency in reporting. For further information on Nature Portfolio policies, see our Editorial Policies and the Editorial Policy Checklist.

Statistics

For all statistical analyses, confirm that the following items are present in the figure legend, table legend, main text, or Methods section.

n/a	Confirmed	
☐	☒	The exact sample size (*n*) for each experimental group/condition, given as a discrete number and unit of measurement
☐	☒	A statement on whether measurements were taken from distinct samples or whether the same sample was measured repeatedly
☐	☒	The statistical test(s) used AND whether they are one- or two-sided<br>*Only common tests should be described solely by name; describe more complex techniques in the Methods section.*
☒	☐	A description of all covariates tested
☐	☒	A description of any assumptions or corrections, such as tests of normality and adjustment for multiple comparisons
☐	☒	A full description of the statistical parameters including central tendency (e.g. means) or other basic estimates (e.g. regression coefficient) AND variation (e.g. standard deviation) or associated estimates of uncertainty (e.g. confidence intervals)
☐	☒	For null hypothesis testing, the test statistic (e.g. *F*, *t*, *r*) with confidence intervals, effect sizes, degrees of freedom and *P* value noted<br>*Give P values as exact values whenever suitable.*
☒	☐	For Bayesian analysis, information on the choice of priors and Markov chain Monte Carlo settings
☒	☐	For hierarchical and complex designs, identification of the appropriate level for tests and full reporting of outcomes
☐	☒	Estimates of effect sizes (e.g. Cohen's *d*, Pearson's *r*), indicating how they were calculated

Our web collection on statistics for biologists contains articles on many of the points above.

Software and code

Policy information about availability of computer code

Data collection

Microscopy:
LAS X software v3.5.7.23225 was used for confocal imaging with the Leica TCS SP8 X
BZ-X8000 Analyzer software was used for the Keyence BZ-X810 inverted fluorescence microscope.

FACS:
Summit v6.3.1 software was used for the sorting on the MoFlo Astrios EQ.
FACSDiva software v9.0 (Becton Dickinson) was used for data acquisition on the BD LSR Fortessa.

Sequencing:
NextSeq 1000/2000 Control Software (NCS) v1.4.1.39716 for the Illumina NextSeq 1000/2000 instrument
Real Time Analysis Software (RTA) v3.9.25 for image analysis and base calling
bcl2fastq v2.20 software for conversion of .bcl files into .fastq files
DRAGEN v3.8.4 software for sequencing and conversion of .bcl files into .fastq files
RNA STAR aligner v2.7.8 for transcriptome alignment (bulkRNA-seq)
CellRanger 7.1.0 for alignment, demultiplexing and count determination

Lipid measurement by LC-MS:
Agilent 1290 Infinity II UHPLC in line with a Bruker Impact II QTOF-MS
MetaboScape (version 2023b) for data processing including feature detection, feature deconvolution and annotation of lipids

MALDI Mass Spectrometry Imaging (MSI):

	timsTOF fleX system (Bruker Daltonics) equipped with a smartbeam 3D 10 kHz laser, TimsControl 5.0(4.1), and flexImaging v7.4(7.2) software (Bruker Daltonics)<br>SCiLS Lab 2024a Pro for root mean square-normalization
Data analysis	Analysis of scRNA-seq data:<br>RStudio (build 421)<br>R version 4.3.2 (2023-10-31)<br>R packages: Seurat 5.0.3, scDblFinder 1.16.0, harmony v 1.2.0, clusterProfiler 4.10.0, EnhancedVolcano 1.20.0, BiomaRt 2.58.2, ggplot2 3.5.0, pheatmap (v1.0.12)<br><br>FastQC 0.73 and TrimGalore! 0.6.7 for processing of bulkRNA fastq files.<br><br>Microscopy:<br>Leica LAS X software v3.5.7.23225<br>Adobe Photoshop CS4<br><br>Statistical analysis:<br>GraphPad Prism v10 was used for graph design and statistical analysis

For manuscripts utilizing custom algorithms or software that are central to the research but not yet described in published literature, software must be made available to editors and reviewers. We strongly encourage code deposition in a community repository (e.g. GitHub). See the Nature Portfolio guidelines for submitting code & software for further information.

Data

Policy information about availability of data

All manuscripts must include a data availability statement. This statement should provide the following information, where applicable:

- Accession codes, unique identifiers, or web links for publicly available datasets
- A description of any restrictions on data availability
- For clinical datasets or third party data, please ensure that the statement adheres to our policy

The raw sequencing data (fastq files), processed sequencing data (filtered feature-barcode matrices), and analyzed Seurat objects for this project are available under xxx. All other data that support the findings of this study are available from the corresponding authors upon reasonable request.

Research involving human participants, their data, or biological material

Policy information about studies with human participants or human data. See also policy information about sex, gender (identity/presentation), and sexual orientation and race, ethnicity and racism.

Reporting on sex and gender	We only used male specimen it the present study.<br>Sex and gender were not disaggregated.
Reporting on race, ethnicity, or other socially relevant groupings	Race, ethnicity, or socially relevant groupings were not considered in the present study.
Population characteristics	Limited information was available of patients. The age ranges from 1-2 years. We included specimen from 2 Sandhoff Disease patients and used age- and sex-matched controls.
Recruitment	The patient samples were provided by the NIH NeuroBioBank upon request.
Ethics oversight	The study protocol was approved by the Ethics committee of the University of Freiburg Medical Center and local ethics committees (associated with the National Institutes of Health biobanks: IRB of the University of Maryland School of Medicine and the IRB of the Department of Health and Mental Hygiene of the State of Maryland)

Note that full information on the approval of the study protocol must also be provided in the manuscript.

Field-specific reporting

Please select the one below that is the best fit for your research. If you are not sure, read the appropriate sections before making your selection.

☒ Life sciences ☐ Behavioural & social sciences ☐ Ecological, evolutionary & environmental sciences

For a reference copy of the document with all sections, see nature.com/documents/nr-reporting-summary-flat.pdf

Life sciences study design

All studies must disclose on these points even when the disclosure is negative.

Sample size	No statistical methods were used to predetermine sample sizes. Sample sizes were chosen empirically based on prior experience with similar

Sample size	experimental designs and effect sizes typically reported in the literature for comparable studies in order to achieve reliable detection of biologically meaningful differences while minimizing unnecessary use of animals.
Data exclusions	scRNAseq: duplets, low-quality cells, and cell expressing multiple cell-type specific marker genes were excluded (see material&methods)
Replication	To be sure of the reproducibility of the experimental findings, all experiments performed with multiple biological replicates were replicated twice successfully.
Randomization	For all experiments, mice were randomly allocated to each experimental group. Human patients were allocated based on their genetic disorder (thus, no randomization is applicable).
Blinding	All quantification experiments were performed in a blinded manner by assignment of unidentifiable numbers to mice, tissues and images for data acquisition and processing. Data labels and groups were only reinstated for statistical analysis. Quantification and imaging was not repeated following statistical analysis.

Reporting for specific materials, systems and methods

We require information from authors about some types of materials, experimental systems and methods used in many studies. Here, indicate whether each material, system or method listed is relevant to your study. If you are not sure if a list item applies to your research, read the appropriate section before selecting a response.

Materials & experimental systems

n/a	Involved in the study
☐	☒ Antibodies
☒	☐ Eukaryotic cell lines
☒	☐ Palaeontology and archaeology
☐	☒ Animals and other organisms
☒	☐ Clinical data
☒	☐ Dual use research of concern
☒	☐ Plants

Methods

n/a	Involved in the study
☒	☐ ChIP-seq
☐	☒ Flow cytometry
☒	☐ MRI-based neuroimaging

Antibodies

Antibodies used

FACS (catalog, clone, supplier, lot):
Fc Block (553141, 2.4G2, BD Biosciences, 3101344): https://www.bdbiosciences.com/en-de/products/reagents/flow-cytometry-reagents/research-reagents/single-color-antibodies-ruo/purified-rat-anti-mouse-cd16-cd32-mouse-bd-fc-block.553141
CD11b (101257, M1/70, BioLegend, B363316): https://www.biolegend.com/de-de/products/brilliant-violet-605-anti-mouse-human-cd11b-antibody-7637
CD45 (47-0451-82, 30-F11, Invitrogen, 2375407): https://www.thermofisher.com/antibody/product/CD45-Antibody-clone-30-F11-Monoclonal/47-0451-82
Ly6C (560593, AL-21, BD Biosciences, 1250627): https://www.bdbiosciences.com/en-nl/products/reagents/flow-cytometry-reagents/research-reagents/single-color-antibodies-ruo/pe-cy-7-rat-anti-mouse-ly-6c.560593
Ly6G (560601, 1A8, BD Biosciences, 2076827): https://www.bdbiosciences.com/en-se/products/reagents/flow-cytometry-reagents/research-reagents/single-color-antibodies-ruo/pe-cy-7-rat-anti-mouse-ly-6g.560601
CD115 (25-1152-82, AFS98, Invitrogen, 2403233): https://www.thermofisher.com/antibody/product/CD115-c-fms-Antibody-clone-AFS98-Monoclonal/25-1152-82
CD64 (139311, X54-5/7.1, BioLegend, B358294): https://www.biolegend.com/de-de/products/brilliant-violet-711-anti-mouse-cd64-fcgammari-antibody-9920
CD11c (25-0114-82, N418, Invitrogen, 2685518): https://www.fishersci.de/shop/products/cd11c-monoclonal-antibody-n418-pe-cyanine7-ebioscience-invitrogen/p-7090310
CD3e (48-0033-82, eBio500A2, Invitrogen, 2527448): https://www.thermofisher.com/antibody/product/CD3e-Antibody-clone-eBio500A2-500A2-Monoclonal/48-0033-82
CD19 (48-0193-82, eBio1D3, Invitrogen, 2686548): https://www.fishersci.de/shop/products/cd19-monoclonal-antibody-ebio1d3-1d3-efluor-450-ebioscience-invitrogen/p-7091076
B220 (103222, RA3-6B2, BioLegend, B357253): https://www.biolegend.com/de-de/products/pe-cyanine7-anti-mouse-human-cd45r-b220-antibody-1930
CD206 (141708, C068C2, BioLegend, B354282): https://www.biolegend.com/de-de/products/apc-anti-mouse-cd206-mmr-antibody-7425
Olig2 (ab225099, EPR2673, abcam, 1079868-1): https://www.abcam.com/en-us/products/primary-antibodies/alexa-fluor-488-olig2-antibody-epr2673-ab225099
NeuN (ab190565, EPR12763, abcam, 1071553-4): https://www.abcam.com/en-us/products/primary-antibodies/alexa-fluor-647-neun-antibody-epr12763-neuronal-marker-ab190565

Histology (catalog, clone, supplier, lot):
IBA1 (ab178846, EPR16588, abcam, 1002201-50): https://www.abcam.com/en-us/products/primary-antibodies/iba1-antibody-epr16588-ab178846
IBA1 (234 308, Gp311H9, Synaptic Systems, 1-22): https://sysy.com/product/234308
Mac-3 (553322, M3/84, BD Pharmingen, 2038820): https://www.bdbiosciences.com/en-us/products/reagents/western-blotting-and-molecular-reagents/purified-rat-anti-mouse-cd107b.553322

GFAP (Z0344, polyclonal, DAKO, 41526076): https://www.agilent.com/en/product/immunohistochemistry/antibodies-controls/primary-antibodies/glial-fibrillary-acidic-protein-(concentrate)-76683
APP (MAB348, 22C11, Merck, 4000311): https://www.merckmillipore.com/DE/de/product/Anti-APP-A4-Antibody-a.a.-66-81-of-APP-NT-clone-22C11,MM_NF-MAB348
P2RY12 (AS-55043A, polyclonal, Anaspec, UB-1701): product has been discontinued. https://doi.org/10.1038/s41586-022-04596-2
TMEM119 (400 002, polyclonal, Synaptic Systems, 1-9): https://sysy.com/product/400002
NeuN (ab104224, 1B7, abcam, GR3408621-1): https://www.abcam.com/en-us/products/primary-antibodies/neun-antibody-1b7-neuronal-marker-ab104224
NeuN (ab177487, EPR12763, abcam, 1001571-6): https://www.abcam.com/en-us/products/primary-antibodies/neun-antibody-epr12763-neuronal-marker-ab177487
HEXB (LS-B16803, polyclonal, LSBio, 225153): https://www.lsbio.com/pathplus-antibodies/pathplus-hexb-antibody-aa294-435-ihc-wb-western-ls-b16803/856183
CD206 (MCA2235, MR5D3, Bio-Rad, 161736): https://www.bio-rad-antibodies.com/monoclonal/mouse-cd206-antibody-mr5d3-mca2235.html
Collagen IV (AB769, polyclonal, Millipore, 3597990): https://www.merckmillipore.com/DE/de/product/Anti-Collagen-Type-IV-Antibody,MM_NF-AB769
SOX9 (AF3075, polyclonal, R&D): https://www.rndsystems.com/products/human-sox9-antibody_af3075
OLIG2 (ab109186, EPR2673, abcam, GR3251505-4): https://www.abcam.com/en-us/products/primary-antibodies/olig2-antibody-epr2673-ab109186
GM2 (A2575, MK1-16, TCI, IBR8B): https://www.tcichemicals.com/DE/de/p/A2576
CD68 (MCA1957, FA-11, Bio-Rad, 155083): https://www.bio-rad-antibodies.com/monoclonal/mouse-cd68-antibody-fa-11-mca1957.html
TuJ1 (302 306, polyclonal, Synaptic Systems, 1.8): https://sysy.com/product/302306
His (MA1-21315, HIS.H8, Invitrogen, YF372500): https://www.thermofisher.com/antibody/product/6x-His-Tag-Antibody-clone-HIS-H8-Monoclonal/MA1-21315
Vimentin (5741, D21H3, Cell Signaling, 8): https://www.cellsignal.com/products/primary-antibodies/vimentin-d21h3-xp-rabbit-mab/5741
LAMP1 (PA1-654A, polyclonal, Invitrogen, YG377034): https://www.thermofisher.com/antibody/product/LAMP1-Antibody-Polyclonal/PA1-654A
KiM1P (self-produced): https://pubmed.ncbi.nlm.nih.gov/1890811/
p22phox (sc-20781, polyclonal , Santa Cruz, FL-195): https://www.scbt.com/de/p/p22-phox-antibody-fl-195
Lysozyme (A0099, polyclonal, DAKO): https://www.agilent.com/store/de_DE/Prod-A009902-2/A009902-2
LAMP2 (MA5-31970, SA46-01, ThermoFisher): https://www.thermofisher.com/antibody/product/CD107b-LAMP-2-Antibody-clone-SA46-01-Recombinant-Monoclonal/MA5-31970
SMI31 (801601, SMI31, Biolegend): https://www.biolegend.com/de-de/products/purified-anti-neurofilament-h-nf-h-phosphorylated-antibody-11476
SMI35 (835604, SMI35, Biolegend): https://www.biolegend.com/de-de/products/purified-anti-neurofilament-h-m-nf-h-nf-m-hypophosphorylated-antibody-12722
SMI312 (837904, SMI312, Biolegend): https://www.biolegend.com/de-de/products/purified-anti-neurofilament-marker-pan-axonal-cocktail-12811

Histology - secondary antibodies (catalog, supplier, lot):
AF488 donkey anti-rabbit IgG (H+L) (A21206, Invitrogen, 2873188)
AF568 donkey anti-rabbit IgG (H+L) (A10042, Invitrogen, 2941306)
AF647 donkey anti-rabbit IgG (H+L) (A31573, Invitrogen, 2752586)
AF488 donkey anti-mouse IgG (H+L) (A21202, Invitrogen, 2563848)
AF568 donkey anti-mouse IgG (H+L) (A10037, Invitrogen, 2555709)
AF647 donkey anti-mouse IgG (H+L) (A31571, Invitrogen, 2555690)
AF488 donkey anti-rat IgG (H+L) (A21208, Invitrogen, 2310102)
AF568 goat anti-rat IgG (H+L) (A11077, Invitrogen, 2379471)
AF488 goat anti-chicken IgY (H+L) (A11039, Invitrogen, 2566343)
AF488 goat anti-guinea pig IgG (H+L) (A11073, Invitrogen, 2674373)
AF568 goat anti-guinea pig IgG (H+L) (A11075, Invitrogen, 2720368)
AF647 goat anti-guinea pig IgG (H+L) (A21450, Invitrogen, 2633525)
AF405 donkey anti-goat IgG (H+L) (A48259, Invitrogen, YB368866)
AF488 F(ab')2 frag. of goat anti-mouse IgG, IgM (H+L) (A10684, Invitrogen, 2892423)
Goat anti-rabbit IgG (H+L) ads-BIOT (4050-08, SouthernBiotech, I5114-N395AD)
Goat anti-rat IgG (H+L) ads-BIOT (3050-08, SouthernBiotech, J4813-MI59B)
Goat anti-mouse IgG (H+L) ads-BIOT (1031-08, SouthernBiotech, I2717-R708D)

Western Blot (catalog, clone, supplier, lot):
TSG101 (ab125011, EPR7130(B), abcam, 1043948-4): https://www.abcam.com/en-us/products/primary-antibodies/tsg101-antibody-epr7130b-ab125011
GAPDH (2118, 14C10, Cell Signaling, 16): https://www.cellsignal.com/products/primary-antibodies/gapdh-14c10-rabbit-mab/2118
His (66005-1-Ig, 1B7G5, Proteintech, 10027680): https://www.ptglab.com/de/products/His-Tag-Antibody-66005-1-Ig.htm

Cell Culture (catalog, clone, supplier, lot):
MGL1/2 (HM1081, ER-MP23, Hycultec, 35686M0323): https://www.hycultbiotech.com/product/mgl-mouse-mab-er-mp23/
IGF2R (AF2447, polyclonal, Biotechne, VSI0322071): https://www.rndsystems.com/products/human-igf-ii-r-igf2r-antibody_af2447
Normal goat IgG Control (AB-108-C, polyclonal, Biotechne, ES4524081): https://www.rndsystems.com/products/normal-goat-igg-control_ab-108-c

Validation

All primary anti-mouse antibodies used in the flow cytometry, western blot or immunostainings have been validated for this application by the supplier and have been used in previous studies. For validation methods and references, please see online information on the product websites by the manufacturer/supplier following the provided links above.

Positive and negative control stainings have been included wherever possible to ensure proper antibody function.

Fluorescence minus one stains or isotype controls were used to define cell-population gates for FACS.

Animals and other research organisms

Policy information about studies involving animals; ARRIVE guidelines recommended for reporting animal research, and Sex and Gender in Research

Laboratory animals	B6.129S-Hexbtm1Rlp/J<br>B6.Hexbtm1c(EUCOMM)Hmgu/H<br>129S4/SvJaeSor-Gt(ROSA)26Sortm1(FLP1)Dym/J<br>B6J.B6N(Cg)-Cx3cr1tm1.1(cre)Jung/J<br>B6.Cg-Tg(Nes-cre)1Kln/J<br>B6.129P2(Cg)-Cx3cr1tm1Litt/J<br>B6.Tg(Thy1-EGFP)MJrs/J<br>B6N.Hexbem1Mp<br>B6.Cg-Tg(SOD1*G93A)1Gur/J<br>B6CBA-Tg(HDexon1)62Gpb/3J<br>B6.Cg-Tg(APPSwFlLon,PSEN1*M146L*L286V)6799Vas/Mmjax<br>B6.Cg-Tg(Thy1-APP)3Somm/J<br><br>Mice from embryonic day (E)14.5 to old adult age (1 year) were used in this study. The exact age is indicated in the respective experimental schemes, figure legends, or method section.<br><br>Mice were housed under a 12-h light/12-h dark cycle and at temperatures of 18–23°C with 40–60% humidity, with food and water provided ad libitum.
Wild animals	None.
Reporting on sex	Mice from both sexes were used in the present study. Sex-specific analysis has not been performed.
Field-collected samples	No field-collected samples were used in this study.
Ethics oversight	Animal studies were approved by the Regional Councils of Freiburg, Germany and performed in accordance to the respective national, federal and institutional regulations.

Note that full information on the approval of the study protocol must also be provided in the manuscript.

Plants

Seed stocks	*Report on the source of all seed stocks or other plant material used. If applicable, state the seed stock centre and catalogue number. If plant specimens were collected from the field, describe the collection location, date and sampling procedures.*
Novel plant genotypes	*Describe the methods by which all novel plant genotypes were produced. This includes those generated by transgenic approaches, gene editing, chemical/radiation-based mutagenesis and hybridization. For transgenic lines, describe the transformation method, the number of independent lines analyzed and the generation upon which experiments were performed. For gene-edited lines, describe the editor used, the endogenous sequence targeted for editing, the targeting guide RNA sequence (if applicable) and how the editor was applied.*
Authentication	*Describe any authentication procedures for each seed stock used or novel genotype generated. Describe any experiments used to assess the effect of a mutation and, where applicable, how potential secondary effects (e.g. second site T-DNA insertions, mosiacism, off-target gene editing) were examined.*

Flow Cytometry

Plots

Confirm that:

☒ The axis labels state the marker and fluorochrome used (e.g. CD4-FITC).

☒ The axis scales are clearly visible. Include numbers along axes only for bottom left plot of group (a 'group' is an analysis of identical markers).

☒ All plots are contour plots with outliers or pseudocolor plots.

☒ A numerical value for number of cells or percentage (with statistics) is provided.

Methodology

Sample preparation	For blood cell analysis, one drop of blood was collected from the facial vein into FACS buffer (PBS containing 2% BSA (8076.3, Roth) and 10mM EDTA (15575020, Invitrogen)) to prevent clotting. Blood cells were centrifuged at 300g for 5 min at 4°C.

	Blood cell pellet was resuspended in RBC lysis buffer (00-4333-57, ThermoFisher) and incubated for 2 min at RT. Ice cold FACS buffer was added and cells were centrifuged again before staining. For microglia analysis, mice were anesthetized and transcardially perfused with ice cold PBS. Brains were roughly minced and homogenized with a potter tissue grinder in HBSS (14170-138, gibco) containing 15 mM HEPES (15630080, gibco) buffer and 0.54% glucose (G8769, Sigma). Whole brain homogenate was separated by 37% Percoll (P1644, Sigma) gradient centrifugation at 800g for 30 min at 4 °C (no brake). The pellet containing CNS macrophages at the bottom of the tube was then collected and washed once with FACS buffer before staining.
Instrument	Cells were sorted using a MoFlo Astrios EQ (Beckman Coulter) or analyzed using a BD LSRFortessa (Becton Dickinson).
Software	Data were acquired with FACSDiva software (Becton Dickinson). Post-acquisition analysis was performed using FlowJo software, version 10.7
Cell population abundance	The cell population abundances are provided in the plots depicting the representative gating strategies.
Gating strategy	In all experiments, small debris was removed with the preliminary FSC/SSC gate. Single, living cells were obtained by doublet exclusion followed by the exclusion of dead cells using DAPI. Representative gating strategies are provided in the respective figures.

☒ Tick this box to confirm that a figure exemplifying the gating strategy is provided in the Supplementary Information.
